# Animal Board Invited Review: Comparing conventional and organic livestock production systems on different aspects of sustainability

**DOI:** 10.1017/S175173111700115X

**Published:** 2017-05-31

**Authors:** C. P. A. van Wagenberg, Y. de Haas, H. Hogeveen, M. M. van Krimpen, M. P. M. Meuwissen, C. E. van Middelaar, T. B. Rodenburg

**Affiliations:** 1 Wageningen Economic Research, PO-box 29703, 2502LS Den Haag, The Netherlands; 2 Animal Breeding and Genomics Centre, Wageningen Livestock Research, PO-box 338, 6700AH Wageningen, The Netherlands; 3 Business Economics Group, Wageningen University, PO-box 8130, 6700EW Wageningen, The Netherlands; 4 Department of Animal Nutrition, Wageningen Livestock Research, PO-box 338, 6700AH Wageningen, The Netherlands; 5 Animal Production Systems group, Wageningen University, PO-box 338, 6700AH Wageningen, The Netherlands; 6 Behavioural Ecology Group, Wageningen University, PO-box 338, 6700AH Wageningen, The Netherlands

**Keywords:** livestock production system, sustainability, literature review, conventional, organic

## Abstract

To sustainably contribute to food security of a growing and richer world population, livestock production systems are challenged to increase production levels while reducing environmental impact, being economically viable, and socially responsible. Knowledge about the sustainability performance of current livestock production systems may help to formulate strategies for future systems. Our study provides a systematic overview of differences between conventional and organic livestock production systems on a broad range of sustainability aspects and animal species available in peer-reviewed literature. Systems were compared on economy, productivity, environmental impact, animal welfare and public health. The review was limited to dairy cattle, beef cattle, pigs, broilers and laying hens, and to Europe, North America and New Zealand. Results per indicators are presented as in the articles without performing additional calculations. Out of 4171 initial search hits, 179 articles were analysed. Studies varied widely in indicators, research design, sample size and location and context. Quite some studies used small samples. No study analysed all aspects of sustainability simultaneously. Conventional systems had lower labour requirements per unit product, lower income risk per animal, higher production per animal per time unit, higher reproduction numbers, lower feed conversion ratio, lower land use, generally lower acidification and eutrophication potential per unit product, equal or better udder health for cows and equal or lower microbiological contamination. Organic systems had higher income per animal or full time employee, lower impact on biodiversity, lower eutrophication and acidification potential per unit land, equal or lower likelihood of antibiotic resistance in bacteria and higher beneficial fatty acid levels in cow milk. For most sustainability aspects, sometimes conventional and sometimes organic systems performed better, except for productivity, which was consistently higher in conventional systems. For many aspects and animal species, more data are needed to conclude on a difference between organic and conventional livestock production systems.

## Implications

This study analysed peer-reviewed literature that compared the sustainability performance of conventional and organic livestock production systems on economy, productivity, environmental performance, animal welfare and public health. No study analysed all aspects simultaneously. For most sustainability aspects, sometimes conventional and sometimes organic systems performed better, except for productivity, which was consistently higher in conventional systems. For many sustainability aspects and animal species, more data are needed to conclude on a difference between the systems.

## Introduction

Global demand for animal source food is expected to be more than 50% higher in 2030 compared with 2000, because of growth of the world population, increased incomes and urbanization, mostly in developing regions (Alexandratos and Bruinsma, 2012). Current livestock production already causes severe pressure on the environment through the use of scarce resources and emission of pollutants. For example, it uses about 70% of the total agricultural land and contributes about 15% to the global anthropogenic greenhouse gas emissions (Steinfeld *et al.*, 2006; Gerber *et al.*, 2013). To sustainably contribute to food security, livestock production systems are challenged to increase production levels reducing their environmental impact, whereas being economically viable and socially responsible. Actions that need to be implemented for sustainable livestock production in and across different systems remain subject to debate. A systematic overview of advantages and disadvantages of existing livestock production systems could provide valuable insights to aid this debate. Although a wide variety of livestock production systems exists, a common, and more studied classification is organic *v*. conventional systems. Conventional livestock production focuses on technologies for increased productivity, such as high-yielding breeds, modern feeding techniques and veterinary health products, and (synthetic) fertilizers and pesticides. In contrast, organic livestock production focuses on cultural, biological and mechanical methods to ensure environmentally safe and chemical residue-free foods, along with high animal welfare standards (Codex Alimentarius Commission, 2007). Reviews have compared conventional and organic livestock production systems on environmental impacts (De Vries *et al.*, 2015), animal welfare (Hovi *et al.*, 2003) and public health (Smith-Spangler *et al.*, 2012). A systematic overview including a broad range of sustainability aspects is lacking. Our study aims to provide this overview. We analysed peer-reviewed articles that compared conventional and organic livestock production systems on economy, productivity, environmental impact, animal welfare and public health. We focused on dairy cattle, beef cattle, pigs, broilers and laying hens, and on regions with production systems comparable with those in North-western Europe. After demarcating sustainability and describing the literature search strategy, we present results of dairy cattle, for which most studies were found, followed by results of the other animal species.

## Demarcation of sustainability

Conventional and organic livestock production systems were compared based on the three pillars economic, environmental and social sustainability (Lebacq *et al.*, 2013). Per pillar, different sustainability aspects and indicators were identified. Results per indicator are presented as in the articles, without additional calculations. Performance per indicator was defined to be significantly different between the systems, if an article reported a *P*-value⩽0.05.

### Economic sustainability

For economic sustainability, indicators related to the aspects *economy* and *productivity* were selected. Indicators related to economy were farm income, costs incurred (variable, fixed, total), farm gate price premium achieved in the market, risk and employability. Indicators related to productivity were not selected before the literature search, but included only if they were considered in an article selected for another sustainability aspect. Productivity indicators considered in one or more articles were the amount of product produced per animal, body weight (BW) gain, protein and fat content, numbers of offspring and feed conversion ratio.

### Environmental sustainability

For environmental sustainability, we used indicators that quantify the impact of livestock production on climate change, eutrophication, acidification, energy use, land use and biodiversity. Environmental sustainability was assessed based on a life-cycle approach, considering the environmental impact of the production chain from extraction of raw materials to produce farm inputs (e.g. feed, fertilizers), manufacturing of these inputs, to all on-farm processes. The impact on climate change, for example, is determined by summing the different greenhouse gases produced along the production chain based on their global warming potential (GWP) in terms of CO_2_ equivalents, expressed per unit product.

### Social sustainability

For social sustainability, indicators related to the aspects *animal welfare* and *public health* were selected. For animal welfare, we used indicators quantifying production system impact on behavioural problems, such as aggression, damaging behaviour and stress sensitivity, and on animal health, such as animal diseases, reproduction and mortality. Indicators on public health were zoonotic microbiological hazards, antimicrobial resistance, chemical hazards and potentially beneficial aspects of food.

## Literature search strategy

The literature search strategy consisted of the general search terms ‘conventional AND organic’ and ‘cattle OR cow OR calf OR calves OR veal OR chicken* OR broiler* OR laying hen* OR pig* OR hog* OR sow OR swine*’, combined with aspect-specific search terms (Table 1). Articles published from 1995 until March 2015 in English that compared organic and conventional livestock production systems were selected. Studies had to be performed in Europe, North America or New Zealand. Only peer-reviewed articles were selected; books, book sections, conference proceedings were excluded. Review articles were excluded, because we focused on original sources of data. Articles without quantitative data were excluded. The databases included in the study were Biological abstracts, CAB abstracts, EconLit, Medline, Scopus and Web of Science. For economy only, the AgEcon database was searched additionally. All 60 initial results in AgEcon were excluded, because none were peer-reviewed articles.

**Table 1:** Aspect-specific search terms

Aspects	Search term
Economy	‘economic performance OR people-planet-profit OR 3-P OR economic and social impacts OR integrated sustainability assessment OR economic feasibility OR economic evaluation OR economic assessment OR risk assessment OR multi-criteria assessment OR employability OR cost price OR profitability’
Environment^1^	‘LCA OR life cycle assessment OR life cycle analysis’
Animal welfare	‘welfare’ and ‘health OR disease* OR mastitis OR lameness OR ketosis OR metabolic disorder* OR reproduction OR fertility’
Public health	‘zoono* OR food safety OR resistance OR human health OR public health OR toxic* OR contamination* OR residue* OR hazard*’

1 Including ‘acidification’, ‘eutrophication’, ‘climate change’, ‘energy use’, ‘ammonia’, ‘nitrate’, ‘methane’, ‘sulphur dioxide’, ‘deforestation’ or ‘land use change’ did not influence search results. Only studies using a life cycle approach were included to ensure that the environmental impact related to production of feed, fertilizers and energy sources, either purchased by the farmer or produced on the farm itself, were included.

## Selected articles

Of the 4171 initial results that were retrieved with the search strategy, 179 articles were finally used to compare organic with conventional livestock production (Table 2). Seven articles addressed indicators in more than one aspect, apart from productivity.

**Table 2:** Initial hits and analysed articles

			Animal welfare^1^			
	Economy	Environment	Welfare	Health	Public health	Total
Number of articles after initial search						
Web of Science	285	29	96	459	162	1031
CAB abstracts	175	31	151	533	199	1089
Biological abstracts	246	18	65	13	176	518
Medline	71	4	33	210	40	358
Scopus	164	1	159	381	764	1469
EconLit	44	1	1	5	0	51
Extra^2^	2	0	0	0	0	2
Total	987	84	505	1258	1337	4171
Number of analysed articles^3^	17	29	52		88	179^4^

1 Two aspect-specific search terms were used for animal welfare, one related to welfare and one to health. Number of articles after initial search of these two aspect-specific search terms could both include the same articles.

2 Retrieved from literature search of other issues.

3 Excluded articles did not comply with the literature search strategy or had a different subject (e.g. bio-energy production, crop production, other animal species, waste and water treatment).

4 This is lower than the sum over articles per aspect (i.e. 186), because seven articles were analysed in more than one aspect.

## Comparison conventional and organic dairy cattle production

### Economy

For economy, eight articles were found on dairy cattle (Table 3 and Supplementary Table S1). Five addressed Europe and three North America. Two were modelling studies, four used panel data and two were case studies. Some articles were very detailed on all indicators, whereas one article only provided aggregate farm income. Articles comprehensively addressing economic issues widely varied in context, research design, definitions and implicit amount of farm labour used. Price premium (six articles), variable costs, total costs and farm income per cow (two) and employability (two) were covered most frequently. Units applied per indicator differed across articles, for example farm income was expressed at farm level with varying farm scales, or using number of hectares. Most studies used more than 10 observations for both conventional and organic systems.

**Table 3:** Minimum and maximum levels of economic indicators in organic livestock expressed relative to those of conventional livestock within the same article

					Value organic relative to conventional (conventional=100)	
Animal types	Economic indicator	Unit	Number of articles	Number of articles with data from <10 conventional (organic) farms^1^	Minimum	Maximum
Dairy cattle	Variable costs	€/cow	2	0 (0)	70	98
		€/ha	1	1 (1)	28	28
	Fixed costs	€/cow	1	0 (0)	82	103
	Total costs	€/cow	2	0 (0)	81	99
		€/cwt^2^	1	0 (0)	166	166
	Price premium	%	6	1 (2)	100	184
	Gross margin	€/cow	1	0 (0)	111	111
		€/farm	1	1 (1)	45	45
		€/ha	1	1 (1)	135	135
	Farm income	€/cow	2	0 (1)	110	534
		€/farm	2	1 (1)	76	166
		€/ha	1	0 (0)	165	165
	Employability	%/cow	1	0 (0)	200	200
		%/cwt	1	0 (0)	104	104
	Risk	Milk price^3^	1	0 (0)	230	230
		Feed price^3^	1	0 (0)	214	214
		Milk yield^3^	1	0 (0)	130	130
Beef cattle	Variable costs	€/head	1	1 (1)	152	152
	Fixed costs	€/head	1	1 (1)	113	113
	Total costs	€/head	1	1 (1)	187	187
	Price premium	%	2	2 (2)	112	125
	Gross margin	€/pen	1	1 (1)	37	37
	Farm income	€/100 head	1	1 (1)	270	270
Broilers	Variable costs	€/kg	2	2 (2)	118	176
	Fixed costs	€/kg	2	2 (2)	166	760
	Total costs	€/kg	2	2 (2)	120	187
	Price premium	%	2	2 (2)	200	207
	Farm income	€/kg	1	1 (1)	1300	1300
		€/fte	1	1 (1)	1043	1043
		€/farm	1	1 (1)	224	224
	Employability	%	1	1 (1)	175	175
Laying hens	Variable costs	€/hen	1	1 (1)	165	165
	Price premium	%	1	1 (1)	239	239
	Gross margin	€/kg	1	1 (1)	123	123
	Farm income	€/fte	1	1 (1)	256	256

1 Number of farms in panel data or case studies. An experiment or model are both considered as one farm.

2 CWT: equivalent milk production.

3 Measured as average detrended within-farm standard deviation.

Consistent findings across articles reflected that organic compared with conventional dairy production had farm gate price premiums (up to 84% above conventional prices), had lower variable (up to 30%) and total (up to 19%) costs per cow, and realised a higher farm income per cow. In contrast, price and yield risk were found to be significantly higher on organic dairy farms. Articles showed ambiguous results with regard to income at farm level.

### Productivity

For productivity, 12 articles were retrieved on dairy cattle (Table 4 and Supplementary Table S2), of which nine addressed Europe and three the USA. In all, 11 studies used data collected on farms, whereas one study used national statistics. Of the 11 farm data studies, three included data of five to 10 conventional and organic farms, five included 11 to 50 comparable farms and three included 50 to 325 comparable farms. In seven articles, organic cows produced significantly less milk per day or per year (range 4.7% to 32.0%) compared with conventional cows, whereas three articles observed no significant difference. Two articles did not statistically test milk yield differences. The lower milk yield of organic cows might have originated from the generally longer and more regulated pasture season (Alvasen *et al.*, 2012), less use of high-yielding breeds (Bennedsgaard *et al.*, 2010) and low levels of concentrate supplementation or conserved forage (Butler *et al.*, 2009). Milk fat content in organic milk was similar in three and significantly lower in one study. Milk protein content in organic milk was similar in one and significantly lower in three studies.

**Table 4:** Minimum and maximum value of performance indicators in organic livestock expressed relative to those of conventional livestock within the same article

				Value organic relative to conventional (conventional=100)	
Animal types	Performance indicator	Number of articles	Number of articles with data from <10 conventional (organic) farms	Minimum	Maximum
Dairy cattle	Milk yield	12	3 (4)	68	95
	Milk fat content	4	0 (0)	96	110
	Milk protein content	4	4 (4)	96	106
Beef cattle	BW gain	2	2 (2)	78	88
Sows	Feed intake	3	2 (2)	120	129
	Number of piglets weaned	4	3 (3)	70	98
Fattening pigs	Feed conversion ratio	3	2 (2)	98	111
Broilers	BW gain	3	3 (3)	76	84
	Feed conversion ratio	3	3 (3)	140	153
Laying hens	Egg production	4	3 (3)	87	99
	Feed conversion ratio	3	2 (2)	106	128

### Environment

For environment, 15 studies were retrieved on dairy production (Table 5 and Supplementary Table S3). Two studies (Halberg *et al.*, 2005; Chen and Corson, 2014) used data from other included studies and were excluded from further analysis. A total of 14 studies addressed Europe and one the USA.

**Table 5:** Average environmental impact (range) per unit product of organic systems relative to conventional systems (conventional=100), and number of articles (*n*)

Animal species	GWP	*n*	AP	*n*	EP	*n*	Land use	*n*	Energy use	*n*	Biodiversity loss	*n*
Dairy cattle	100 (83 to 120)	12	109 (87 to 160)	6	103 (64 to 160)	6	149 (108 to 190)	10	71 (60 to 93)	5	54 (24 to 95)	3
Beef cattle	86 (68 to 97)	3	164	1	146	1	116 (107 to 122)	2	56	1	–	–
Pigs	129 (90 to 172)	4	82 (30 to 130)	3	117 (30 to 130)	4	220 (170 to 311)	4	114 (90 to 140)	3	–	–
Broilers	104 (72 to 150)	5	166 (150 to 196)	3	205 (200 to 240)	4	230 (189 to 315)	4	118 (86 to 159)	4	–	–
Laying hens	95 (56 to 130)	4	132 (110 to 154)	3	162 (130 to 185)	2	189 (166 to 220)	2	109 (87 to 140)	3	–	–

GWP=global warming potential; AP=acidification potential; EP=eutrophication potential.

In all, 12 studies assessed the impact on climate change. On average, GWP per unit milk was the same (0% difference) for organic and conventional systems (range −17% to 20%; Table 5). Generally, organic systems had a higher enteric methane emission per unit milk because of a lower milk yield per cow and an increased use of roughage. In contrast, emissions of CO_2_ and nitrous oxide were lower in organic systems due to the absence of synthetic fertilizer, lower nitrogen application levels and a relatively low use of concentrates, resulting in a similar overall GWP. Differences between studies mainly related to methodological differences and differences in assumptions on production data. For example, in their simulation model, Del Prado *et al.* (2011) assumed that milk yield per cow was the same for both systems, resulting in a 17% lower GWP per unit milk in organic systems. In contrast, Capper *et al.* (2008) found a lower milk yield per cow (−25%) in organic systems, resulting in a 13% higher GWP per unit milk in organic systems. Capper *et al.* (2008) emphasised the importance of dilution of maintenance in reducing the environmental impact of animal production.

Six studies assessed the impact on acidification, which is mainly related to ammonia emission from manure in stables, in storage, during grazing and after fertilizer application. On average, acidification potential (AP) was higher (9%) for organic than for conventional systems (range −13% to 60%). The average was highly influenced by a 60% higher AP for organic systems reported by Williams *et al.* (2006). Excluding this study, AP of both systems was comparable (−1%). Williams *et al.* (2006) do not provide an explanation for the higher AP in organic systems. Thomassen *et al.* (2008) and Capper *et al.* (2008) explained the higher AP per unit milk in organic systems by a lower milk yield per cow. The other studies did not provide a clear explanation.

The studies assessing the impact on acidification also assessed the impact on eutrophication, which is mainly related to leaching of nitrate and phosphate and to emissions of ammonia from manure and synthetic fertilizers. Eutrophication potential (EP) per unit milk was on average 3% higher in organic systems (range −36% to 60%). Excluding the 60% higher EP for organic systems reported by Williams *et al.* (2006), EP of organic systems was 9% lower. Generally, organic systems resulted in a lower EP per unit milk due to the absence of synthetic fertilizer and lower nitrogen and phosphate fertilization levels. The lower EP per unit milk in organic systems (−36%) found by Thomassen *et al.* (2008) was explained by the conventional farms being located on sandy soils with a higher net nitrogen leaching factor and the organic farms on clay and peat soils with a lower factor. The higher EP per unit milk in organic systems in the other studies was explained by the accumulation of phosphate in the soil limiting the possibility to reduce leaching of phosphate, the use of feed products with a high EP (peas) and a lower milk yield per cow.

A total of 10 studies assessed the impact on land use, which includes on- and off-farm land for animal feed production. Land use per unit milk was consistently higher (49%) in organic compared with conventional systems (range 8% to 90%). This was explained by lower crop (grass) yields per ha and lower milk yield per cow. Variation between studies was large, mainly due to differences in diet composition, grass yields and milk yields.

Five studies assessed the impact on fossil energy use. Fossil energy use per unit milk was consistently lower in organic (−29%) compared with conventional systems (range −40% to −7%). This was explained by the absence of synthetic fertilizers and a relatively low use of concentrates. Both production and transport of concentrates are important contributors to energy use.

Three studies assessed the impact on biodiversity. All found the impact per unit milk to be lower in organic compared with conventional systems, despite the larger areas of land that organic systems required (range −76% to −5%). This was explained by the absence of pesticides and synthetic fertilizer, a lower stocking rate per hectare, and a better balance between cutting, grazing and the level of external inputs in organic systems.

In addition to the environmental impact per unit milk, some studies also assessed the impact per hectare land. Although product-based indicators are a measure for production efficiency, area-based indicators provide insight into the potential local impact. Three studies provided results on the nitrogen and phosphorus surplus per hectare (Cederberg and Mattsson, 2000; Thomassen *et al.*, 2008; Van der Werf *et al.*, 2009). In all three studies, impacts were significantly lower for organic systems (results not shown). This was related mainly to the absence of synthetic fertilizers and lower fertilization levels.

### Animal welfare

For animal welfare, 47 articles addressed dairy cattle (Table 6 and Supplementary Table S4). Of these articles, 37 were from Europe including 24 articles from Scandinavian countries, nine from the USA and one from New Zealand. Two articles described experiments, both based on one analysis in which one herd was split in two parts that were either organically or conventionally managed. All other studies were observational. Two observational studies followed herds before and after transition to organic. These were descriptive in nature and no statistical analyses were performed. The other observational studies compared conventional with (matched) organic herds. In all, 12 studies explored existing databases. Other studies collected on-farm data, such as milk samples (nine), blood samples (six) and faecal samples (five). Six studies performed animal observations. In some studies, on-farm or routine health data were combined with data from questionnaires about farm management.

**Table 6:** Summary of differences in animal welfare indicators between conventional and organic livestock production

		Number of articles			
Animal species	Welfare indicator	Total	Significant difference (*P*<0.05)	Data from <10 conventional (organic) herds/flocks	Value organic relative to conventional of significant differences (conventional=100)
Dairy cattle	Somatic cell count (×1000 cells/ml)	8	3	1 (1)	106, 107, 133^1^
	Clinical mastitis (incidence/year %)	4	2	0 (0)	48, 55
	Mastitis (prevalence bacteriology after parturition)	1	1	1 (1)	149
	Mastitis (prevalence positive California Mastitis Test)	1	1	0 (0)	122
	Ketosis (incidence/year %)	4	2	0 (0)	36, 54
	Milk fever (incidence/year %)	3	2	0 (0)	52, 59
	Endometritis (incidence/year %)	1	1	0 (0)	56
	Retained placenta (incidence/year %)	2	1	0 (0)	64
	Helminths *Ostertagia ostertagi* (optical density ratio)	1	1	0 (0)	124
	Calving interval (days)	4	3	0 (0)	99, 102, 102
	Longevity (days of productive life)	1	1	0 (0)	106
	Culling (% of cows per year)	2	2	0 (0)	75, 86
	Mortality (rate)	2	1	0 (0)	0^2^
	Lying time (% of total time)	1	1	0 (0)	88
	Hock lesions (prevalence %)	1	1	0 (0)	32
	Activity (prevalence %)	1	1	0 (0)	121
	Aggression feeding gate (frequency)	1	1	0 (0)	124
Beef cattle	Reproductive disorders (prevalence %)	1	1	0 (0)	950
Laying hens	Worm infections (prevalence %)	1	1	0 (0)	275
Pigs	Worm infections (prevalence %)	1	1	0 (0)	165
	Leg problems (prevalence %)	1	1	0 (0)	21
	Haptoglobin (blood concentration)	1	0	1(1)	–
	Lactate (concentration)	1	1	1(1)	67
Broilers	Latency to lie (s)	1	1	1 (1)	222
	Hock lesions (lesion score)	1	1	1 (1)	33
	Footpad lesions (prevalence %)	1	0	1(1)	6
	Acute phase proteins (blood concentration)	1	1	1 (1)	114
	Newcastle disease (mean antibody titers)	1	1	0 (1)	60
	Infectious Bursitis (mean antibody titers)	1	1	0 (1)	232
	Infectious Bronchitis (mean antibody titers)	1	1	0 (1)	200

1 Article provided a higher somatic cell count of 50 000 cells/ml on organic farms, given a population average of 150 000 cells/ml.

2 On organic farms mortality was 0 and on conventional farms incidence of mortality was 16% per year.

A total of 15 welfare indicators were studied, with 14 articles on mastitis, six each on metabolic status and production diseases incidence, five on reproduction and three each on longevity/mortality, *Salmonella,* and claw and leg health. Only Langford *et al.* (2011) described a behavioural study on lying behaviour and aggression. Sample seizes differed considerably over the articles, from blood samples of 22 organic and 18 conventional cows to data on 5335 conventional and 402 organic herds from national databases. Almost all studies used decent multivariable regression models, correcting for possible confounders, to evaluate differences between conventional and organic farms. In some studies at herd level, the number of farms was too small to correct for possible confounders.

Of the eight articles on somatic cell counts, three showed significantly higher counts on organic compared with conventional farms. Also a study on bacteriology after parturition and a study using the California mastitis test showing significantly lower level of udder health on organic farms. In contrast, two out of four articles on clinical mastitis levels showed lower levels on organic farms (Hardeng and Edge, 2001; Valle *et al.*, 2007). These two studies also found lower levels of clinical ketosis on organic farms. However, three other studies on blood metabolites showed hardly any differences. Often, studies on clinical diseases are based upon farmer reported disease incidences or veterinary reported treatments. The farmer’s disease definition and decisions regarding antibiotic treatment are important factors in such studies. One study (Richert *et al.*, 2013) corrected results of farmers’ reported disease incidences for their disease definition. After correction, differences between farm systems disappeared, indicating the importance of the farmers’ disease definition in these types of studies. Thus, care should be taken when drawing conclusions in studies using farmer reported disease data. Of the five studies on reproduction (all on large databases), three found better reproductive results on conventional farms. The three studies on *Salmonella* were based upon one large data analysis of over 100 farms and did not show any differences. The three studies on foot and leg health differed too much to draw conclusions.

### Public health

For microbiological hazards (Supplementary Table S5), 15 articles addressed dairy cattle of which nine addressed Europe and seven the USA (one addressed both) (Table 7). All studies compared samples taken at conventional and organic farms or retail locations. Samples originated from less than 10 organic farms in five articles and from less than 10 conventional farms in three articles. Two articles did not mention numbers. Garcia and Teixeira (2017) stated that a variety of factors, such as farm location, season, time before processing or method used for isolation and detection could influence the microbial quality in livestock and food products. Most articles did not consider all possible confounders. Many hazards were addressed, but most in only a few studies. *Escherichia coli* (seven) and *Staphylococcus* (seven) were addressed most often, followed by total bacteria counts (three), *Streptococcus* (three) and coliform bacteria count (two). The studies showed one hazard with significantly higher and one hazard with significantly lower contamination in conventional compared with organic systems, and no difference for 23 hazards (Table 7). This is in line with Wilhelm *et al.* (2009), who could not conclude on a difference, due to contradictory findings across studies.

**Table 7:** Summary of reviewed articles comparing microbiological hazards between organic and conventional livestock production

Animal species	Number of articles	Number of articles with data from <10 conventional (organic) sampling locations	Hazards addressed^**1**^	Sampling location hazards
Dairy cattle	15	3 (5) Not mentioned 1 (1)	Bacteria negative (1, nd 1), *Campylobacter* spp. (1, nd 1,), coliform bacteria count (1, nd 1), coliform organisms (1, np 1), *Cryptosporidium* spp. (2, nd 2,), *Enterococcus* spp. (1, nd 1), *Escherichia coli* (3, nd 3), *E. coli* O157 (1, nd 1), *Listeria monocytogenes* (1, nd 1), *Salmonella* spp. (1, nd 1), shiga Toxin-encoding bacteria (2, nd 2), shiga Toxigenic *E. coli* (2, nd 1, np 1), spore forming bacteria (*Bacillus*) (1, c>o 1), *STEC* O157:H7 (1, np 1), *Staphylococcus* (1, nd 1), *Staphylococcus aureus* (6, nd 4, np 2), *Streptococcus dysgalactiae* (1, nd 1), *Streptococcus uberis* (1, nd 1), *Streptococcus* other (1, nd 1), total bacteria count (1, np 1), total mesophilic bacteria count (1, o>c 1) Total hazards addressed 31, with c>o 1, o>c 1, nd 23, np 6	Farm 27, retail 4
Beef cattle	3	1 (2) Not mentioned 1 (1)	Condemnations of digestive tract (1, o>c 1), heart (1, nd 1), kidney (1, c>o 1), leg (1, o>c 1), liver (1, c>o 1) and lung (1, c>o 1), Enterobacteriaceae (1, nd 1), *E. coli* (1, nd 1), *L. monocytogenes* (1, nd 1), mesophilic aerobic bacteria (1, o>c 1), *Salmonella* spp. (1, np 1), *S. aureus* (1, nd 1) Total hazards addressed 12, with c>o 3, o>c 3, nd 5, np 1	Slaughterhouse 6, retail 6
Pigs	9	1 (6) Not mentioned 0 (0)	*Campylobacter* (1, np 1), Enterobacteriaceae (1, nd 1), *E. coli* (2, o>c 2), Hepatitis E virus (3, o>c 1, np 2), *L. monocytogenes* (6, o>c 5, np 1), mesophilic aerobic bacteria (1, nd 1), *Salmonella* (4, nd 3, np 1),*Yersinia pseudotuberculosis* (7, o>c 1, np 6), *Yersinia enterocolitica* (5, c>o 3, nd 1, np 1) Total hazards addressed 28, with c>o 3, o>c 9, nd 6, np 12	Farm 12, slaughterhouse 12, retail 4
Broilers	19	5 (14) Not mentioned 2 (2)	Aerobic bacteria (1, np 1), *Campylobacter coli* (1, np 1), *Campylobacter jejuni* (1, np 1), *Campylobacter* spp. (15, o>c 4, nd 2, np 9), enterobacteriaceae (2, c>o 1, o>c 1), *Enterococcus* spp. (2, o>c 1, np 1), *E. coli* (2, o>c 2), faecal coliforms (1, c>o 1), *L. monocytogenes* (2, nd 2), mesophilic aerobic bacteria (1, nd 1), psychotrophs (1, o>c 1), *Salmonella* spp. (14, c>o 2, o>c 2, nd 3, np 7), *S. aureus* (2, nd 1, np 1), *Staphylococcus* spp. (1, np 1) Total hazards addressed 46, with c>o 4, o>c 11, nd 9, np 22	Farm 14, slaughterhouse 9, retail 23
Laying hens	6	3 (3) Not mentioned 1 (1)	Aerobic bacteria (2, o>c 1, nd 1), *Campylobacter* spp. (1, np 1), *Citrobacter* (1, np 1), coliforms (1, nd 1), *Enterobacter* (1, np 1), Enterobacteriaceae (1, nd 1), *Enterococcus* spp. (4, nd 1, np 3), *E. coli* spp. (1, np 1), gram-negative bacteria (1, c>o 1), *Listeria* spp. (3, np 3), moulds and yeasts (1, nd 1), Pantoea (1, np 1), *Pseudomonas* spp. (1, nd 1), psychotrophs (1, nd 1), *Salmonella enterica* (1, np 1), *Salmonella enteritidis* (1, np 1), *Salmonella* spp. (2, np 2), *Staphylococcus* spp. (1, c>o 1), total microorganisms (3, np 3) Total hazards addressed 28, with c>o 2, o>c 1, nd 7, np 18	Farm 16, retail 12

Number of times in all articles: c>o=number of times conventional higher than organic; o>c=number of times organic higher than conventional; nd=number of times no difference; np=number of times no quantitative *P*-value.

1 Only non-zero values mentioned.

For antimicrobial resistance (Supplementary Table S6), 20 articles concerned dairy cattle of which six articles addressed Europe and 15 the USA (one addressed both) (Table 8). All studies compared samples between conventional and organic farms or products. Samples were taken at farms (20 bacteria) and in retail outlets (one). Samples originated from less than 10 farms or retail locations in seven articles for organic and four articles for conventional systems. Three articles did not mention numbers. In all, 12 studies used milk samples (from teats, bulk milk tank, milk filters, milk line), 12 studies manure samples (rectal swap, manure lagoon, manure storage, on floor) and two to three studies samples from the water source, feed bunks or housing. Attribution of differences in antimicrobial resistance to the production system was complicated, because most studies lack correction for other potential sources of contamination, such as animals, people, vehicles or wildlife at farms (Ray *et al.*, 2006) and the environment or people during processing (Miranda *et al.*, 2009). Antimicrobial resistance in many individual bacteria was analysed, but most in only a few studies. *Staphylococcus* (11) and *E. coli* (seven) were analysed most, followed by *Campylobacter* (three), *Streptococcus* (two) and all other hazards (each one). Resistance to many different antibiotics was measured, complicating comparison across studies. Over all bacteria–antibiotic combinations analysed in the articles, 26 bacteria showed higher resistance to an antibiotic in conventional systems, whereas only five bacteria showed higher resistance to an antibiotic in organic systems (Table 8). Bacteria more often showed significantly higher multidrug resistance in conventional (two) compared with organic systems (zero) than vice versa, although the number of studies was limited (Table 8). This is consistent with Wilhelm *et al.* (2009), who concluded that antimicrobial resistance was lower in organic dairy production. The main explanation for higher levels of antimicrobial resistance in conventional systems was higher use of antimicrobials.

**Table 8:** Summary of reviewed articles comparing antimicrobial resistance between organic and conventional livestock production

Animal species	Number of articles	Number of articles with data from <10 conventional (organic) sampling locations	Number of times bacteria addressed in all articles	Sampling location bacteria	Higher single drug resistance (number of bacteria–drug combinations over all studies)^1^	Higher multidrug resistance (number of multidrug-resistant bacteria over all studies)^2^
Dairy cattle	20	4 (7) Not mentioned 3 (3)	Bacteria 1, *Campylobacter* 1, *Campylobacter* spp. 2, coagulase-negative staphylococci 2, ESBL-producing *Escherichia coli* 1, *E. coli* 4, *E. coli* O157 1, genes (tet(O), tet(W), sul (I), sul(II)) 1, nonaureus *staphylococcus* spp. 1, *Salmonella* spp. 1, Shiga Toxigenic *E. coli* 1, *Staphylococcus* 1, *Staphylococcus aureus* 7, *Streptococcus uberis* 1, *Streptococcus dysgalactiae* 1	Farm 20, retail 1	O>C 5 C>O 26	O>C 0 C>O 2
Beef cattle	2	0 (0) Not mentioned 2 (2)	Enterobacteriaceae 1, *E. coli* 1, *Listeria monocytogenes* 1, mesophillic aerobic bacteria 1, *S. aureus* 1	Retail 4	O>C 2 C>O 6	O>C 0 C>O 0
Pigs	5	2 (2) Not mentioned 2 (3)	*Campylobacter* spp. 1, Enterobacteriaceae 1, *Enterococcus faecium* 1, *Enterococcus* spp. 1, *E. coli* 3, mesophillic aerobic bacteria 1, vancomycin-resistant *E. faecium* 1	Farm 4, retail 2	O>C 0 C>O 4	O>C 0 C>O 1
Broilers	20	10 (7) Not mentioned 8 (9)	*Campylobacter* spp. 6, Enterobacteriaceae 2, *E. faecium* 2, *Enterococcus faecalis* 1, *Enterococcus* spp. 1, ESBL 1, ESBL-producing bacteria 1, *E. coli* 4, *E. coli* carrying bla(CMY-2) 1, *E. coli* carrying bla(CTX-M) 1, *L. monocytogenes* 1, mesophilic aerobic bacteria 1, quinolone-resistant determining regions in *E. coli* 1, *Salmonella* 1, *Salmonella* spp. carrying bla(CMY-2) 1, *Salmonella Kentucky* 1, *Salmonella* spp. 4, *S. aureus* 1, vancomycin-resistant *Enterococci* 1, vancomycin-resistant *E. faecium* 1	Farm 5, processing 3, retail 20	O>C 5 C>O 50	O>C 0 C>O 8
Laying hens	3	0 (0) Not mentioned 1 (1)	*Campylobacter coli* 1, *Campylobacter jejuni* 1, *Enterococcus* spp. 1, *E. coli* 2, *Listeria* spp. 1	Farm 5, retail 1	O>C 3 C>O 13	O>C 0 C>O 2

1 O>C: over all bacteria–drug combinations analysed in the articles, number of times bacteria in organic livestock system were more resistant to a specific drug than bacteria in conventional livestock system. C>O vice versa.

2 O>C: number of times bacteria in organic livestock system showed more multidrug resistance than in conventional livestock system. C>O vice versa.

For chemical hazards (Supplementary Table S7), nine articles on dairy cattle in Europe were reviewed. Three studies took samples at farms, one at a slaughter plant and five at retail locations. Samples analysed differed between articles, for example two studies at farm level analysed milk, and one study hair and blood. Hazards analysed differed widely: four articles analysed heavy metals, two organochlorine pesticides and ochratoxin A and one DDT. Too few articles were found per chemical hazard to conclude on differences between the systems.

For potentially beneficial aspects (Supplementary Table S8), nine articles on dairy cattle were reviewed of which eight concerned Europe and one the USA. Seven studies took samples at farms, one at a slaughter plant, and one at retail locations. Four farm level studies used samples from less than 10 organic and conventional farms, and one study did not mention the number of farms. The slaughter plant study compared organic with conventional cows originating from one research station. The retail level study mentioned total number of samples, but not number of locations or brands. The studies did not correct for all potentially confounding variables, such as breed and animals’ energy status. Indicators analysed varied widely, with fatty acids including CLA (seven) and essential elements (four) being analysed most, followed by vitamins (two). Four studies indicated a better beneficial fatty acid composition in organic dairy milk. Two studies showed a mixed picture with better composition in organic for some fatty acids and better composition in conventional for other fatty acids. One study did not find a difference. O’Donnell *et al.* (2010) indicated that detected differences were not of physiological importance. Generally, the studies indicated a better beneficial fatty acid composition in organic dairy milk. The studies related this to a higher amount of grazing and fresh forage of organic cows. This is consistent with the conclusions of Rembiałkowska and Średnicka (2009). For essential elements, studies are inconclusive.

## Comparison conventional and organic production for beef cattle, pigs, broilers and laying hens

### Economy

For economy, findings for other species’ systems (Table 3 and Supplementary Table S1) were consistent with those of dairy farming systems with regard to farm gate prices and farm income. Organic prices were up to 25%, 107% and 139% above conventional prices for beef cattle, broilers and laying hens, respectively. Organic farm income was up to 170%, 124% and 156% higher for beef cattle per head, broilers per farm, and laying hens per full time equivalent, respectively. No data were found on pigs. In contrast to dairy farming, variable costs were higher for organic compared with conventional systems.

### Productivity

Productivity of pigs was mostly lower in organic systems, consistent with the dairy cattle studies. Feed intake level of organic sows was 20% to 29% higher, and number of piglets weaned per sow was 2% to 30% lower (Table 4 and Supplementary Table S2). Feed intake and feed conversion ratio of organic fattening pigs was similar or higher than of conventional fattening pigs. Too few studies were found on differences in productivity between organic and conventional beef cattle, broilers and laying hens systems to extrapolate to entire sectors.

### Environment

For environment, three studies were found on beef cattle, nine on pigs, four on laying hens and five on broilers (Table 5 and Supplementary Table S3). Due to the limited number of studies per environmental impact category and livestock species, extrapolation to entire sectors is difficult. Climate change differences between organic and conventional livestock production varied across species. On average, organic systems had a lower GWP per unit product for beef cattle, a similar GWP for boilers and laying hens and a higher GWP for pigs. The lower productivity levels (crops and animals) in organic systems resulted in higher impact, but lower fertilization levels and absence of synthetic fertilizer in a lower impact. In case of acidification and eutrophication, impacts per unit product were higher in organic systems across all species, except for the AP of beef cattle, which was lower. Lower productivity levels in organic systems were the main cause. Land use per unit product was consistently higher in organic systems for all species. Energy use was lower in organic systems for beef cattle, but higher for laying hens, broilers and pigs. Differences in energy use between livestock species related to differences in diet and the ability of ruminants to use grass and other roughage products that can be produced with little energy.

### Animal welfare

For animal welfare, eight studies were found on the other species than dairy cattle in Europe (Table 6 and Supplementary Table S4). Six studies were observational and two experimental. Studies focused on different welfare indicators, making a sound comparison of these studies and extrapolation to entire sectors impossible. Across species, three topics were identified with more than one study: leg health (three), general health and resistance (three) and worm infections (two). In both sows (Knage-Rasmussen *et al.*, 2014) and broilers (Tuyttens *et al.*, 2008), a higher incidence of leg problems was found on conventional farms. In broilers, this was mainly related to the use of slower growing or more robust genotypes, and in sows, to increased activity due to outdoor access. Three studies showed improved stress resistance in organic broilers and pigs, explained by different genetics, increased space per animal, and outdoor access. Finally, two studies showed more worm infections in organic pigs and in laying hens housed in non-cage systems (either conventional or organic), which the studies explained by increased contact with manure and free range access.

### Public health

Quite some articles addressed microbiological hazards in broilers (19), whereas less addressed pigs (nine), laying hens (six), or beef cattle (three) (Table 7 and Supplementary Table S5). Hazards differed between animal species, because studies generally focused on the most important microbial hazards for each animal species, which differ between animal species. *Campylobacter* (17) and *Salmonella* (14) were addressed most in broiler articles, and *Yersinia* (12) and *Listeria monocytogenes* (six) in pig articles. In contrast to dairy cattle studies, more broiler and pig studies showed significantly lower microbial contamination in conventional compared with organic systems than significantly higher (broiler 11 lower *v*. four higher, pigs nine lower *v*. three higher). Van Loo *et al.* (2012) also concluded that organically produced meat is more often contaminated with foodborne pathogens than conventionally produced meat. The few studies on beef cattle and laying hens showed no differences. Most studies lacked correction for all confounders. For example, studies mentioned hygiene during manufacturing and processing to be important for microbiological contamination at retail level in beef (Miranda *et al.*, 2009), chicken meat (e.g. Mazengia *et al.*, 2014) and eggs (e.g. Álvarez-Fernández *et al.*, 2012), but did not correct for this. These studies could not be used to conclude about contamination at farm level. This could explain why Smith-Spangler *et al.* (2012) concluded that bacterial contamination in retail chicken and pig meat was unrelated to the farming method. Additional studies published after publication of Smith-Spangler *et al.* (2012) do not solve this, because they also lack of correction for all confounders.

Quite some studies addressed antimicrobial resistance in broilers (20), and only few pigs (five), laying hens (three) or beef cattle (two) (Table 8 and Supplementary Table S6). Antimicrobial resistance in many individual bacteria was analysed, bacteria analysed in the studies differed between animal species, and individual bacteria were addressed in few studies maximally. Results for the other species were comparable to dairy cattle: over all bacteria–antibiotic combinations analysed in the articles, more often bacteria in conventional systems showed a significantly higher resistance to a single antibiotic or a significantly higher multidrug resistance compared with bacteria in organic systems than vice versa. Smith-Spangler *et al.* (2012) suggested higher resistance among bacteria isolated from conventional chicken and pig meat, although differences were not statistically significant. Additional studies published as Smith-Spangler *et al.* (2012) further strengthen this suggestion, especially on chicken production. Van Loo *et al.* (2012) also concluded that bacteria isolated from conventionally produced livestock or meats may have a higher likelihood of antimicrobial resistance. Our findings of higher multidrug resistance in conventional chicken and pig production are in line with those of Smith-Spangler *et al.* (2012).

Few articles were found on chemical hazards for pigs (two), broilers (two), laying hens (two) and beef cattle (one) addressing different hazards (Supplementary Table S7), and only one article on laying hens in Europe addressing potentially beneficial aspects (Supplementary Table S8). Therefore, it was not possible to generalize on differences in chemical hazards and beneficial aspects between the systems.

## General discussion

Conventional and organic livestock production systems were compared on different aspects of sustainability, including economy, productivity, environmental impact, animal welfare and public health. For many sustainability aspects and animal species, insufficient data were found to conclude on differences between the systems. But, some differences were identified. Conventional systems had lower labour requirements per unit product, lower income risk per animal, higher production per animal per time unit, higher reproduction numbers, lower feed conversion ratio, lower land use, generally lower AP and EP per unit product, equal or better udder health and equal or lower microbiological contamination. Organic systems had higher income per animal or full time employee, lower AP and EP per unit land, lower impact on biodiversity per unit product, equal or lower likelihood of antibiotic resistance in bacteria, and higher beneficial fatty acid levels in cow milk. Overall, this comparison indicates both systems have strong and weak points. Combining the strong points of both systems into a hybrid system could contribute to increase the sustainability performance of livestock production.

For many sustainability aspects and animal species, extrapolation of results to conclude on a difference between organic and conventional livestock production systems was hampered by four reasons. First, for most sustainability indicators only a limited number of studies was available. Second, large differences existed between studies in design, sampling location, sample size and measurement methods. Harmonization of designs, sampling strategies and measurement methods to assess sustainability performance of farming systems, therefore, could improve the interpretation of results over studies. Third, quite a few studies used samples from a few farms, processing or retail locations from each production system. And fourth, both organic and conventional livestock producers have to comply with system-specific legal requirements and standards that can vary across regions and countries. Therefore, both organic and conventional farming practices can differ between studies, even though they were categorized in the same production system group.

Improving production efficiency of crops and livestock has been a major focus of livestock production in the last decades. To sustain the improved feed efficiency, the amount of human-edible plant products, such as cereal grains, in livestock diets has increased. To achieve future food security, it is important to recognize that direct consumption by humans of such products is more efficient than consumption of animal source food produced by livestock fed with these cereals (Godfray *et al.*, 2010; Foley *et al.*, 2011). However, livestock production can play an important role in food security by transforming products that humans cannot or do not want to eat, into high-quality food products. Sustainable livestock production, therefore, also implies feeding livestock by-products and waste-stream from arable production or the food processing industry, and grazing of livestock on marginal land (Eisler *et al.*, 2014; Van Zanten *et al.*, 2016). Accounting for the competition between feed and food, including the suitability of land to produce food crops, is important when assessing sustainability of livestock production systems.

The data retrieved in our study are only a part of all data needed to indicate which livestock production system is better. To compare sustainability performance between such systems, sustainability indicators must be weighed relative to each other. This could be done by policy makers assigning a weight to each indicator (Van Asselt *et al.*, 2014). Policy makers with different viewpoints are likely to assign different weighing factors to a specific indicator, resulting in a different sustainability outcome. Establishing broadly accepted weighing factors could facilitate decision making for sustainable livestock production.

The sustainability performance of a livestock production system on an aspect of sustainability can be influenced by the selected indicators. For example, conventional systems were found to have a lower AP and EP per *product unit*, but a higher AP and EP per *land area* compared with organic systems. Thus, indicator selection can have relevant consequences for results. To prevent misunderstanding the meaning of a selected indicator should be clearly communicated and explained when discussing sustainability performance of livestock production systems.

## Conclusions

We reviewed 179 articles that compared the sustainability performance of conventional and organic livestock production systems. Studies varied widely in indicators, research design, sample size and location and context. Quite some studies used small samples. Most articles were found for dairy cattle. No study was found that simultaneously analysed aspects of sustainability for economy, productivity, environmental impact, animal welfare and public health. For most sustainability aspects, sometimes conventional and sometimes organic systems performed better. For productivity, conventional systems outperformed organic systems on all indicators. For many sustainability aspects and animal species, more data are needed to conclude on a difference between organic and conventional livestock production systems.

## Supplementary material

For supplementary material accompanying this paper visit http://dx.doi.org/10.1017/S175173111700115X. click here to view supplementary material

## References

[ref1] Alexandratos N and Bruinsma J 2012 World agriculture towards 2030/2050: the 2012 revision. FAO, ESA working paper No. 12-03. Rome, Italy. Retrieved on 13 December 2016 from http://www.fao.org/docrep/016/ap106e/ap106e.pdf.

[ref2] Álvarez-Fernández E, Domínguez-Rodríguez J, Capita R and Alonso-Calleja C 2012 Influence of housing systems on microbial load and antimicrobial resistance patterns of *Escherichia coli* isolates from eggs produced for human consumption. Journal of Food Protection 75, 847–853. 22564932 10.4315/0362-028X.JFP-11-182

[ref3] Alvasen K, Mork MJ, Sandgren CH, Thomsen PT and Emanuelson U 2012 Herd-level risk factors associated with cow mortality in Swedish dairy herds. Journal of Dairy Science 95, 4352–4362. 22818448 10.3168/jds.2011-5085 PMC7127405

[ref4] Bennedsgaard TW, Klaas IC and Vaarst M 2010 Reducing use of antimicrobials – experiences from an intervention study in organic dairy herds in Denmark. Livestock Science 131, 183–192.

[ref5] Butler G, Collomb M, Rehberger B, Sanderson R, Eyre M and Leifert C 2009 Conjugated linoleic acid isomer concentrations in milk from high- and low-input management dairy systems. Journal of the Science of Food and Agriculture 89, 697–705.

[ref6] Capper JL, Castañeda-Gutiérrez E, Cady RA and Bauman DE 2008 The environmental impact of recombinant bovine somatotropin (rbST) use in dairy production. Proceedings of the National Academy of Sciences of the United States of America 105, 9668–9673. 18591660 10.1073/pnas.0802446105 PMC2442129

[ref7] Cederberg C and Mattsson B 2000 Life cycle assessment of milk production – a comparison of conventional and organic farming. Journal of Cleaner Production 8, 49–60.

[ref8] Chen X and Corson MS 2014 Influence of emission-factor uncertainty and farm-characteristic variability in LCA estimates of environmental impacts of French dairy farms. Journal of Cleaner Production 81, 150–157.

[ref9] Codex Alimentarius Commission 2007 Organically produced foods. World Health Organization and Food and Agriculture Organization of the United Nations, Rome, Italy.

[ref10] De Vries M, Van Middelaar CE and De Boer IJM 2015 Comparing environmental impacts of beef production systems: a review of life cycle assessments. Livestock Science 178, 279–288.

[ref11] Del Prado A, Misselbrook T, Chadwick D, Hopkins A, Dewhurst RJ, Davison P, Butlerd A, Schröder J and Scholefield D 2011 SIMS DAIRY: a modelling framework to identify sustainable dairy farms in the UK. Framework description and test for organic systems and N fertiliser optimisation. Science of the Total Environment 409, 3993–4009. 21703662 10.1016/j.scitotenv.2011.05.050

[ref12] Eisler MC, Lee MRF, Tarlton JF, Martin GB, Beddington J, Dungait JAJ, Greathead H, Liu J, Mathew S, Miller H, Misselbrook T, Murray P, Vinod VK, Robert VS and Michael W 2014 Steps to sustainable livestock. Nature 507, 32–34. 24605375 10.1038/507032a

[ref13] Foley JA, Ramankutty N, Brauman KA, Cassidy ES, Gerber JS, Johnston M, Mueller ND, O/‘Connell C, Ray DK, West PC, Balzer C, Bennett EM, Carpenter SR, Hill J, Monfreda C, Polasky S, Rockstrom J, Sheehan J, Siebert S, Tilman D and Zaks DPM 2011 Solutions for a cultivated planet. Nature 478, 337–342. 21993620 10.1038/nature10452

[ref14] Garcia JM and Teixeira P 2017 Organic versus conventional food: a comparison regarding food safety. Food Reviews International 33, 424–446.

[ref15] Gerber PJ, Steinfeld H, Henderson B, Mottet A, Opio C, Dijkman J, Falcucci A and Tempio G 2013 Tackling climate change through livestock – A global assessment of emissions and mitigation opportunities. Food and Agriculture Organization of the United Nations, Rome, Italy Retrieved on 13 December 2016 from http://www.fao.org/3/a-i3437e/index.html.

[ref16] Godfray HCJ, Beddington JR, Crute IR, Haddad L, Lawrence D, Muir JF, Pretty J, Robinson S, Thomas SM and Toulmin C 2010 Food security: the challenge of feeding 9 billion people. Science 327, 812–818. 20110467 10.1126/science.1185383

[ref17] Halberg N, Van Der Werf HMG, Basset-Mens C, Dalgaard R and De Boer IJM 2005 Environmental assessment tools for the evaluation and improvement of European livestock production systems. Livestock Production Science 96, 33–50.

[ref18] Hardeng F and Edge VL 2001 Mastitis, ketosis, and milk fever in 31 organic and 93 conventional Norwegian dairy herds. Journal of Dairy Science 84, 2673–2679. 11814023 10.3168/jds.S0022-0302(01)74721-2

[ref19] Hovi M, Sundrum A and Thamsborg SM 2003 Animal health and welfare in organic livestock production in Europe: current state and future challenges. Livestock Production Science 80, 41–53.

[ref20] Knage-Rasmussen KM, Houe H, Rousing T and Sorensen JT 2014 Herd- and sow-related risk factors for lameness in organic and conventional sow herds. Animal 8, 121–127. 24168821 10.1017/S1751731113001900

[ref21] Langford FM, Rutherford KMD, Sherwood L, Jack MC, Lawrence AB and Haskell MJ 2011 Behavior of cows during and after peak feeding time on organic and conventional dairy farms in the United Kingdom. Journal of Dairy Science 94, 746–753. 21257042 10.3168/jds.2010-3309

[ref22] Lebacq T, Baret P and Stilmant D 2013 Sustainability indicators for livestock farming. A review. Agronomy for Sustainable Development 33, 311–327.

[ref23] Mazengia E, Samadpour M, Hill HW, Greeson K, Tenney K, Liao G, Huang X and Meschke JS 2014 Prevalence, concentrations, and antibiotic sensitivities of Salmonella serovars in poultry from retail establishments in Seattle, Washington. Journal of Food Protection 77, 885–893. 24853509 10.4315/0362-028X.JFP-13-394

[ref24] Miranda JM, Mondragon A, Vázquez BI, Fente CA, Cepeda A and Franco CM 2009 Microbiological quality and antimicrobial resistance of *Escherichia coli* and *Staphylococcus* aureus isolated from conventional and organic ‘Arzua-Ulloa’ cheese. Cyta – Journal of Food 7, 103–110.

[ref25] O’Donnell AM, Spatny KP, Vicini JL and Bauman DE 2010 Survey of the fatty acid composition of retail milk differing in label claims based on production management practices. Journal of Dairy Science 93, 1918–1925. 20412905 10.3168/jds.2009-2799

[ref26] Ray KA, Warnick LD, Mitchell RM, Kaneene JB, Ruegg PL, Wells SJ, Fossler CP, Halbert LW and May K 2006 Antimicrobial susceptibility of Salmonella from organic and conventional dairy farms. Journal of Dairy Science 89, 2038–2050. 16702267 10.3168/jds.S0022-0302(06)72271-8

[ref27] Rembiałkowska E and Średnicka D 2009 Organic food quality and impact on human health. Agronomy Research 7, 719–727.

[ref28] Richert RM, Cicconi KM, Gamroth MJ, Schukken YH, Stiglbauer KE and Ruegg PL 2013 Risk factors for clinical mastitis, ketosis, and pneumonia in dairy cattle on organic and small conventional farms in the United States. Journal of Dairy Science 96, 4269–4285. 23684015 10.3168/jds.2012-5980

[ref29] Smith-Spangler C, Brandeau ML, Hunter GE, Bavinger JC, Pearson M, Eschbach PJ, Sundaram V, Liu H, Schirmer P, Stave C, Olkin I and Bravata DM 2012 Are organic foods safer or healthier than conventional alternatives? A systematic review. Annals of Internal Medicine 157, 348–366. 22944875 10.7326/0003-4819-157-5-201209040-00007

[ref30] Steinfeld H, Gerber P, Wassenaar T, Castel V, Rosales M and de Haan C 2006 Livestock’s long shadow: environmental issues and options. Food and Agriculture Organization, Rome, Italy Retrieved on 13 December 2016 from http://www.fao.org/docrep/010/a0701e/a0701e00.HTM.

[ref31] Thomassen MA, Van Calker KJ, Smits MCJ, Iepema GL and De Boer IJM 2008 Life cycle assessment of conventional and organic milk production in the Netherlands. Agricultural Systems 96, 95–107.

[ref32] Tuyttens F, Heyndrickx M, De Boeck M, Moreels A, Van Nuffel A, Van Poucke E, Van Coillie E, Van Dongen S and Lens L 2008 Broiler chicken health, welfare and fluctuating asymmetry in organic versus conventional production systems. Livestock Science 113, 123–132.

[ref33] Valle PS, Lien G, Flaten O, Koesling M and Ebbesvik M 2007 Herd health and health management in organic versus conventional dairy herds in Norway. Livestock Science 112, 123–132.

[ref34] Van Asselt ED, Van Bussela LGJ, Van der Voet H, Van der Heijden GWAM, Tromp SO, Rijgersberg H, Van Evertb F, Van Wagenberg CPA and Van der Fels-Klerx HJ 2014 A protocol for evaluating the sustainability of agri-food production systems – a case study on potato production in peri-urban agriculture in The Netherlands. Ecological Indicators 43, 315–321.

[ref35] Van der Werf HMG, Kanyarushoki C and Corson MS 2009 An operational method for the evaluation of resource use and environmental impacts of dairy farms by life cycle assessment. Journal of Environmental Management 90, 3643–3652. 19664872 10.1016/j.jenvman.2009.07.003

[ref36] Van Loo EJ, Alali W and Ricke SC 2012 Food safety and organic meats. Annual Review of Food Science and Technology 3, 203–225. 10.1146/annurev-food-022811-101158 22385165

[ref37] Van Zanten HHE, Mollenhorst H, Klootwijk CW, Van Middelaar CE and De Boer IJM 2016 Global food supply: land use efficiency of livestock systems. The International Journal of Life Cycle Assessment 21, 747–758.

[ref38] Wilhelm B, Rajić A, Waddell L, Parker S, Harris J, Roberts KC, Kydd R, Greig J and Baynton A 2009 Prevalence of zoonotic or potentially zoonotic bacteria, antimicrobial resistance, and somatic cell counts in organic dairy production: current knowledge and research gaps. Foodborne Pathogens and Disease 6, 525–539. 19422303 10.1089/fpd.2008.0181

[ref39] Williams AG, Audsley E and Sandars DL 2006 Energy and environmental burdens of organic and non-organic agriculture and horticulture. Aspects of Applied Biology 79, 19–23.

