# Supplementary material for: Animal Board Invited Review: Comparing conventional and organic livestock production systems on different aspects of sustainability
Source: Animal. 2017 May 31;11(10):1839–51. doi: 10.1017/S175173111700115X (PMC5607874; doi:10.1017/S175173111700115X)
Supplement: Supplementary file 1 [file S175173111700115Xsup.zip › S175173111700115Xsup001/S175173111700115Xsup001.docx]

**Animal Board Invited review: Comparing conventional and organic livestock production systems on different aspects of sustainability**

C.P.A. van Wagenberg, Y. de Haas, H. Hogeveen, M.M. van Krimpen, M.P.M. Meuwissen, C.E. van Middelaar, T.B. Rodenburg

***Supplementary Table S1:*** *Reviewed studies comparing economic performance of organic and conventional livestock farms (significant numbers in bold)*

| **Reference** | **Country** | **Sample^1^** | **Costs** | | | | **Price Premium (%)** | **Farm income**  **(%)^2^** | **Employ- ability (%)** | **Risk** | **Explanation of difference** |
| --- | --- | --- | --- | --- | --- | --- | --- | --- | --- | --- | --- |
|  |  |  | **Unit** | **Variable**  **(%)** | **Fixed**  **(%)** | **Total**  **(%)** |  |  |  |  |  |
| *Dairy cattle* |  |  |  |  |  |  |  |  |  |  |  |
| Berentsen *et al.* (2012) | Netherlands | Panel, 2001/7, 46(O), 302(C) | Cow^-1^ | **-30**^3^ |  |  | +15 | **+11**^a^ cow^-1^  **-24**^b^ farm^-1^ |  | **+** | Lower costs due to more stable health situation. Higher risk due to higher variability of milk price, concentrate feed price, milk yields per cow, and gross margin per cow. |
| Jaklič *et al.* (2014) | Slovenia | Case, 1(O), 1(C) | Ha^-1^ | -72 |  |  |  | +35^a^ ha^-1^ |  |  | Higher market price, lower production costs of grazing-based systems, higher public payments. |
| Kiefer *et al.* (2014) | Germany | Model |  |  |  |  |  | +66^b^ farm^-1^ |  |  | n.a. |
| McBride and Greene (2009) | USA | Panel, 2005, 325(O), 1435(C) | Cwt^-1^ |  |  | **+66** | **+44** | **-**^b^ cwt^-1^ | **+100** |  | Less competitive due to smaller scale. |
| O’Hara and Parsons (2013) | USA | Panel, 2008/10, 33 (O), 129 (C) | Cow^-1^ | -19 | -18 | -19 | +57 | +374^b^ cow^-1^ | +4^4^ |  | Lower feed expenses due to farm feed. Lower veterinary and interest costs. Higher labour costs. |
|  | Canada | Panel, 2009/11, 32 (O), 379 (C) | Cow^-1^ | -2 | +3 | -1 | +84 | +434^b^ cow^-1^ | +4^4^ |  | Higher costs of labour. Lower freight and trucking costs. |
| Pazek and Rozman (2008) | Slovenia | Model |  |  |  |  | +27 | -55^a^ farm^-1^ |  |  | n.a. |
| Stonehouse *et al.* (2001) | Canada | Panel, 92/4, 7(O), 111(C) | Cow^-1^ |  |  | -13^5^ | 0 | +10^b^ cow^-1^ |  |  | Lower material input costs including feed and dairy herd replacements. |
| Tranter *et al.* (2007) | United Kingdom | Case, 27 farms considering conversion |  |  |  |  | +20 | +65^b^ ha^-1^ |  |  | Highly dependent on price premium. |
|  |  |  |  |  |  |  |  |  |  |  |  |
| *Beef cattle* |  |  |  |  |  |  |  |  |  |  |  |
| Bjorklund *et al.* (2014) | USA | Experiment, 16(O), 16(C) | Pen^-1^ |  |  | +87 | +12 | -63^a^ pen^-1^ |  |  | Lower veterinary costs. Extremely high corn and soybean prices. |
| Fernández and Woodward (1999) | USA | Experiment, 18(O), 12(C) | Head^-1^ | +52 | +13 | + |  |  |  |  | Higher feed and yardage (labour, equipment, bedding) costs. |
|  |  |  |  |  |  |  |  |  |  |  |  |
| Greer *et al.* (2008) | New Zealand | Panel, 2002/6, 36(O), 36(C) | Ha^-1^ | - |  |  |  | 0 ha^-1^ |  |  | Lower veterinary and fertiliser costs. (Potential to expand benefits in case of improved farm management.) |
|  |  |  |  |  |  |  |  |  |  |  |  |
| Salevid and Kumm (2011) | Sweden | Model | 100 head^-1^ | + | + |  | +25^7^ | +170^b^  100 head^-1^ |  |  | n.a. |
|  |  |  |  |  |  |  |  |  |  |  |  |
| *Broilers* |  |  |  |  |  |  |  |  |  |  |  |
| Bokkers and De Boer (2009) | Netherlands | Model^8^ | Bird^-1^ | + | + | + | +107 | +943^b^ fte^-1^ | +75 |  | Higher costs of labour, health, feed, bedding. Lower costs of buildings and equipment. Very dependent on market prices and feed costs. |
| Castellini *et al.* (2012) | Italy | Case, 2(O), 2(C) | Kg^-1^ | +18 | +66 | +20 |  | +1 200^b^ kg^-1^ |  |  | Higher feed costs due to lower feed efficiency. |
| Cobanoglu *et al.* (2014) | Turkey | Experiment, 400(O), 400(C) | Kg^-1^ | +76 | +660 | +87 | +100 | +124^b^ farm^-1^ | + |  | Higher costs of feed (mainly due to 50-100% higher prices), labor (slow growing birds, less animals per fte due to organic regulations, less mechanisation), outdoor maintenance, certification. |
|  |  |  |  |  |  |  |  |  |  |  |  |
| *Laying hens* |  |  |  |  |  |  |  |  |  |  |  |
| Dekker *et al.* (2011) | Netherlands | Model^9^ | Hen^-1^ | +65 | - |  | +139 | +156^b^ fte^-1^ |  |  | Much higher egg price, higher feed price, lower # hens per fte. Lower building costs. Higher land investments. |
| Leenstra *et al.* (2014) | Netherlands, Switzerland, France | Model |  |  |  |  |  | +23^a^ kg^-1^ |  |  | n.a. |

^1^ For panel data numbers refer to period covered and # farms. For case studies and experiments, numbers indicate number of farms and number of animals involved respectively.

^2^ Reflected as gross margin (a) or whole farm income (b). Cwt: equivalent milk production.

^3^ Veterinary costs.

^4^ Direct jobs and indirectly induced jobs in the state.

^5^ Excluding costs of land.

^6^ Conventional system is suckler cow based requiring a relatively larger animal stock per kg meat.

^7^ Including government aid for organic production.

^8^ Whole chicken is assumed to be sold as organic.

^9^ Conventional system is a cage system.

n.a.: not available

**References**

Berentsen PBM, Kovacs K and Van Asseldonk MAPM 2012. Comparing risk in conventional and organic dairy farming in the Netherlands: an empirical analysis. Journal of Dairy Science 95, 3803 - 3811.

Bjorklund EA, Heins BJ, DiCostanzo A and Chester-Jones H 2014. Growth, carcass characteristics, and profitability of organic versus conventional dairy beefsteers. Journal of Dairy Science 97, 1817 - 1827.

Bokkers EAM and De Boer IJM 2009. Economic, ecological, and social performance of conventional and organic broiler production in the Netherlands. British Poultry Science 50, 546 - 557.

Castellini C, Boggia A, Cortina C, Dal Bosco A, Paolottib L, Novelli E and Mugnai C 2012. A multicriteria approach for measuring the sustainability of different poultry production systems. Journal of Cleaner Production 37, 192-201.

Cobanoglu FI, Kucukyilmaz K, Cinar M, Bozkurt M, Catli AU and Bintas E 2014. Comparing the Profitability of Organic and Conventional Broiler Production. Brazilian Journal of Poultry Science 16, 89 - 96.

Dekker SEM, De Boer IJM, Vermeij I, Aarnink AJA and Groot Koerkamp PWG 2011. Ecological and economic evaluation of Dutch egg production systems. Livestock Science 139, 109-121.

Fernández MI and Woodward BW 1999. Comparison of conventional and organic beef production systems I. Feedlot performance and production costs. Livestock Production Science 61, 213-223.

Greer G, Kaye-Blake W, Zellman E and Parsonson-Ensor C 2008. Comparison of the financial performance of organic and conventional farms. Jouranl of Organic System 3, 17-27.

Jaklič, Tina, Juvančič, Luka, Kavčič, Stane, Debeljak and Marko 2014. Multiple-perspective performance analysis of dairy production systems in Slovenia*.* Paper presented at EAAE Congress, Ljubljana, Slovenia.

Kiefer L, Menzel F and Bahrs E 2014. The effect of feed demand on greenhouse gas emissions and farm profitability for organic and conventional dairy farms Journal of Dairy Science 97, 7564 – 7574.

Leenstra FR, Maurer V, Galea F, Bestman MWP, Amsler Z, Visscher J, Vermeij I and Van Krimpen MM 2014. Laying hen performance in different production systems; why do they differ and how to close the gap? Results of discussions with groups of farmers in The Netherlands, Switzerland and France, benchmarking and model calculations. European Poultry Science 78, 1 - 10.

McBride WD and Greene C 2009. Costs of Organic Milk Production on U.S. Dairy Farms. Applied Economic Perspectives And Policy 31, 793 - 813.

O’Hara JK and Parsons RL 2013. The economic value of organic dairy farms in Vermont and Minnesota. Journal of Dairy Science 96, 6117 – 6126.

Pazek K and Rozman Č 2008. The economic feasibility of conventional and organic farm production in Slovenia. Agriculturae Conspectus Scientificus 73, 37 - 41.

Salevid P and Kumm K-I 2011. Searching for economically sustainable Swedish beef production systems based on suckler cows after decoupling EU income support. Outlook on Agriculture 40, 131 - 138.

Stonehouse DP, Clark EA and Ogini YA 2001. Organic and Conventional Dairy Farm Comparisons in Ontario, Canada. Biological Agriculture & Horticulture: An International Journal for Sustainable Production Systems 19, 115 - 125.

Tranter RB, Holta GC and Greya PT 2007. Budgetary implications of, and motives for, converting to organic farming: Case study farm business evidence from Great Britain. Biological Agriculture & Horticulture: An International Journal for Sustainable Production Systems 25, 133 - 151.
