# Supplementary material for: Animal Board Invited Review: Comparing conventional and organic livestock production systems on different aspects of sustainability
Source: Animal. 2017 May 31;11(10):1839–51. doi: 10.1017/S175173111700115X (PMC5607874; doi:10.1017/S175173111700115X)
Supplement: Supplementary file 1 [file S175173111700115Xsup.zip › S175173111700115Xsup001/S175173111700115Xsup002.docx]

**Animal Board Invited review: Comparing conventional and organic livestock production systems on different aspects of sustainability**

C.P.A. van Wagenberg, Y. de Haas, H. Hogeveen, M.M. van Krimpen, M.P.M. Meuwissen, C.E. van Middelaar, T.B. Rodenburg

***Supplementary Table S2:*** *Reviewed articles comparing productivity aspects in organic and conventional livestock production*

| Reference | Study country | Sample point | # units/samples: conventional (organic) | Significant effects | Explanation |
| --- | --- | --- | --- | --- | --- |
| *Dairy cattle* |  |  |  |  |  |
| Adler *et al.* (2013) | Norway | farm | 14 (14) paired farms | Organic farms had lower concentrate intake and tended to have lower milk yield. Milk fat and protein contents were not affected. |  |
| Bennedsgaard *et al.* (2003) | Denmark | farm | 99 (82) paired farms | Milk yield on organic farms was significantly reduced in most of the years included in the study. |  |
| Bennedsgaard *et al.* (2010) | Denmark | farm | 35 (21) neighbouring farms | Milk yield was only numerical but not significant different between both systems. | Organic diets had higher forage proportion, lower crude protein content in forage, and lower levels of concentrate. Interaction with breed. Limited percentage of HF-blood, but more often the use of Jersey breeds in organic flocks. |
| Berentsen *et al.* (2012) | Netherlands | farm panel data | 302 (46) farms | Milk yield and milk fat content were not significantly different between organic and conventional farms. Organic farms had significant lower milk protein contents. |  |
| Bloksma *et al.* (2008) | Netherlands | farm | 5 (5) neighbouring farms | Differences in productivity were not statistically tested. |  |
| Butler *et al.* (2009) | Wales | farm | 5 (5) farms | Organic farms had significant lower values for milk yield, milk fat and milk protein contents, compared to conventional farms. | Organic diets had higher forage proportion, lower crude protein content in forage, and lower levels of concentrate. |
| Cho *et al.* (2006) | USA (Minnesota) | farm | 20 (8) farms | Organic farms had significant lower milk yield, compared to conventional farms. |  |
| Cicconi-Hogan *et al.* (2013) | USA (New York, Wisconsin, Oregon) | farm | 36 (192) size matched farms | Organic farms had significant lower milk yield, compared to conventional farms. |  |
| Fall and Emanuelson (2009) | Sweden | farm | 20 (20) farms | Organic farms had significant lower milk yield, compared to conventional farms. |  |
| McBride and Greene (2009) | USA | farm panel data | 1 435 (325) farms | Organic farms had significant lower milk yield, compared to conventional farms. |  |
| Roesch *et al.* (2006) | Switzerland | farm | 60 (60) size matched neighbouring farms | Organic farms had significant lower values for milk yield, and milk protein contents, compared to conventional farms, whereas milk fat was comparable between both systems. |  |
| van Calker *et al.* (2007) | Netherlands | farm | Data of 1 (1) experimental farm, representing the Dutch conventional and organic milk sector | Differences in productivity were not statistically tested. |  |
|  |  |  |  |  |  |
| *Beef cattle* |  |  |  |  |  |
| Bjorklund *et al.* (2014) | USA (Minnesota) | calves | 16 (16) calves | Body weight gain of conventional beef cattle was significant higher compared to organic beef cattle. | Poorer forage quality and pasture drought conditions in organic husbandry. |
| Casey and Holden (2006) | Ireland | farm | 5 (5) farms | Differences in productivity were not statistically tested. | The used organic breed is selected for meat quality rather than for daily gain. |
|  |  |  |  |  |  |
| *Pigs* |  |  |  |  |  |
| Basset-Mens *et al.* (2006) | France | farm panel data | farm | Differences in productivity were not statistically tested. |  |
| Dourmad *et al.* (2014) | Denmark, Netherlands, Spain, France, Germany | farm | 25 (25) farms | Organic pig reproduction farms weaned significantly lower number of piglets per sow, compared to conventional farms, whereas feed intake per sow was numerically but not significantly higher. Feed conversion ratio of organic growing – finishing pigs was significantly higher compared to conventional pigs. | Longer nursery period in organic systems. |
| Lindgren *et al.* (2013) | Sweden | farm | 5 (5) neighbouring farms | Organic pig reproduction farms weaned significantly lower number of piglets per sow, compared to conventional farms. | Longer nursery period in organic systems. |
| Millet *et al.* (2004) | Belgium | Pig | 32 (32) pigs | Conventional pigs had a significant better feed conversion compared to organic pigs. |  |
| van der Werf *et al.* (2007) | France | farm panel data | farm | Differences in productivity were not statistically tested. |  |
|  |  |  |  |  |  |
| *Broilers* |  |  |  |  |  |
| Boggia *et al.* (2010) | Italy | farm panel data | farm | Differences in productivity were not statistically tested. |  |
| Bokkers and De Boer (2009) | Netherlands | farm panel data | farm | Differences in productivity were not statistically tested. |  |
| Castellini *et al.* (2012) | Italy | farm | 2 (2) farms | Differences in productivity were not statistically tested. | The use of slow-growing strains in organic broiler husbandry is primarily responsible for the difference in productive performance. It is more difficult to meet the nutritional requirements in organic husbandry. |
|  |  |  |  |  |  |
| *Laying hens* |  |  |  |  |  |
| Dekker *et al.* (2011) | Netherlands | farm panel data | farm | Differences in productivity were not statistically tested. |  |
| Englmaierová *et al.* (2014) |  | bird | 72 (72) birds | Significant reduced levels of egg production and feed conversion in the organic system compared to the system with conventional cages. |  |
| Leenstra *et al.* (2012) | Switzerland, France, Netherlands | farm | 114 (159) farms | Both systems performed similar in Switzerland and France, whereas organic layers in the Netherlands produced significant less eggs compared to conventional layers. |  |
| Leinonen and Kyriazakis (2013) | UK | farm panel data | farm | Differences in productivity were not statistically tested. |  |

**References**

Adler SA, Jensen SK, Govasmark E and Steinshamn H 2013. Effect of short-term versus long-term grassland management and seasonal variation in organic and conventional dairy farming on the composition of bulk tank milk. Journal of Dairy Science 96, 5793-5810.

Basset-Mens C, van der Werf HMG, Durand P and Leterme P 2006. Implications of uncertainty and variability in the life cycle assessment of pig production systems. International Journal of Life Cycle Assessment 11, 298-304.

Bennedsgaard TW, Klaas IC and Vaarst M 2010. Reducing use of antimicrobials - Experiences from an intervention study in organic dairy herds in Denmark. Livestock Science 131, 183-192.

Bennedsgaard TW, Thamsborg SM, Vaarst M and Enevoldsen C 2003. Eleven years of organic dairy production in Denmark: herd health and production related to time of conversion and compared to conventional production. Livestock Production Science 80, 121-131.

Berentsen PBM, Kovacs K and Van Asseldonk MAPM 2012. Comparing risk in conventional and organic dairy farming in the Netherlands: an empirical analysis. Journal of Dairy Science 95, 3803 - 3811.

Bjorklund EA, Heins BJ, DiCostanzo A and Chester-Jones H 2014. Growth, carcass characteristics, and profitability of organic versus conventional dairy beefsteers. Journal of Dairy Science 97, 1817 - 1827.

Bloksma J, Adriaansen-Tennekes R, Huber M, Van de Vijver LPL, Baars T and De Wit J 2008. Comparison of organic and conventional raw milk quality in the Netherlands. Biological Agriculture & Horticulture 26, 69-83.

Boggia A, Paolotti L and Castellini C 2010. Environmental impact evaluation of conventional, organic and organic-plus poultry production systems using life cycle assessment. World’s Poultry Science Journal 66, 95-114.

Bokkers EAM and De Boer IJM 2009. Economic, ecological, and social performance of conventional and organic broiler production in the Netherlands. British Poultry Science 50, 546 - 557.

Butler G, Collomb M, Rehberger B, Sanderson R, Eyre M and Leifert C 2009. Conjugated linoleic acid isomer concentrations in milk from high- and low-input management dairy systems. Journal of the Science of Food and Agriculture 89, 697-705.

Casey JW and Holden NM 2006. Greenhouse gas emissions from conventional, agri-environmental scheme, and organic Irish suckler-beef units. Journal of Environmental Quality 35, 231-239.

Castellini C, Boggia A, Cortina C, Dal Bosco A, Paolottib L, Novelli E and Mugnai C 2012. A multicriteria approach for measuring the sustainability of different poultry production systems. Journal of Cleaner Production 37, 192-201.

Cho S, Diez-Gonzalez F, Fossler CP, Wells SJ, Hedberg CW, Kaneene JB, Ruegg PL, Warnick LD and Bender JB 2006. Prevalence of shiga toxin-encoding bacteria and shiga toxin-producing Escherichia coli isolates from dairy farms and county fairs. Veterinary Microbiology 118, 289-298.

Cicconi-Hogan KM, Gamroth M, Richert R, Ruegg PL, Stiglbauer KE and Schukken YH 2013. Risk factors associated with bulk tank standard plate count, bulk tank coliform count, and the presence of Staphylococcus aureus on organic and conventional dairy farms in the United States. Journal of Dairy Science 96, 7578-7590.

Dekker SEM, Aarnink AJA, De Boer IJM and Groot Koerkamp PWG 2011. Emissions of ammonia, nitrous oxide, and methane from aviaries with organic laying hen husbandry. Biosystems Engineering 110, 123-133.

Dourmad JY, Ryschawy J, Trousson T, Bonneau M, Gonzalez J, Houwers HWJ, Hviid M, Zimmer C, Nguyen TLT and Morgensen L 2014. Evaluating environmental impacts of contrasting pig farming systems with life cycle assessment. Animal 8, 2027-2037.

Englmaierová M, Tůmová E, Charvátová V and Skřivan M 2014. Effects of laying hens housing system on laying performance, egg quality characteristics, and egg microbial contamination. Czech Journal of Animal Science 59, 345-352.

Fall N and Emanuelson U 2009. Milk yield, udder health and reproductive performance in Swedish organic and conventional dairy herds. Journal of Dairy Research 76, 402-410.

Leenstra F, Maurer V, Bestman M, van Sambeek F, Zeltner E, Reuvekamp B, Galea F and van Niekerk T 2012. Performance of commercial laying hen genotypes on free range and organic farms in Switzerland, France and The Netherlands. British Poultry Science 53, 282-290.

Leinonen I and Kyriazakis I 2013. Quantifying the environmental impacts of UK broiler and egg production systems. Lohmann Information 48, 45-50.

Lindgren Y, Lundeheim N, Boqvist S and Magnusson U 2013. Reproductive performance in pigs reared under organic conditions compared with conventionally reared pigs. Acta Veterinaria Scandinavica 55, 4.

McBride WD and Greene C 2009. Costs of Organic Milk Production on U.S. Dairy Farms. Applied Economic Perspectives And Policy 31, 793 - 813.

Millet S, Hesta M, Seynaeve M, Ongenae E, De Smet S, Debraekeleer J and Janssens GPJ 2004. Performance, meat and carcass traits of fattening pigs with organic versus conventional housing and nutrition. Livestock Production Science 87, 109-119.

Roesch M, Doherr MG and Blum JW 2006. Management, feeding, production, reproduction and udder health on organic and conventional Swiss dairy farms. Schweizer Archiv Fur Tierheilkunde 148, 387-395.

van Calker KJ, Berentsen PBM, de Boer IJM, Giesen GWJ and Huirne RBM 2007. Modelling worker physical health and societal sustainability at farm level: An application to conventional and organic dairy farming. Agricultural Systems 94, 205-219.

van der Werf HMG, Tzilivakis J, Lewis K and Basset-Mens C 2007. Environmental impacts of farm scenarios according to five assessment methods. Agriculture Ecosystems & Environment 118, 327-338.
