# Supplementary material for: Animal Board Invited Review: Comparing conventional and organic livestock production systems on different aspects of sustainability
Source: Animal. 2017 May 31;11(10):1839–51. doi: 10.1017/S175173111700115X (PMC5607874; doi:10.1017/S175173111700115X)
Supplement: Supplementary file 1 [file S175173111700115Xsup.zip › S175173111700115Xsup001/S175173111700115Xsup003.docx]

**Animal Board Invited review: Comparing conventional and organic livestock production systems on different aspects of sustainability**

C.P.A. van Wagenberg, Y. de Haas, H. Hogeveen, M.M. van Krimpen, M.P.M. Meuwissen, C.E. van Middelaar, T.B. Rodenburg

**Supplementary Table S3:** Reviewed studies^1^ comparing environmental impacts between conventional and organic livestock production

| Reference | Environmental impact ^2^ | Study country | Study type | # units/samples | Functional unit / system boundary | Impact conven-tional ^3^ | Impact  Organic ^2^ | Significance |
| --- | --- | --- | --- | --- | --- | --- | --- | --- |
| *Dairy* |  |  |  |  |  |  |  |  |
| Capper *et al.* (2008) | GWP, AP, EP, land use | USA | LCA based on national statistics | Average US farm data from national databases (# farms unknown) | Total milk requirement for US population at the farm gate | GWP: 121 x 10^9^ kg CO_2_eq  AP: 650 x 10^6^ kg SO_2_eq  EP: 452 x 10^9^ PO_4_eq  LU: 10.3 x 10^6^ ha | Ratios org. to convent.  GWP: 1.13  AP: 1.15  EP: 1.28  LU: 1.30 | Not tested |
| Cederberg and Mattsson (2000) | GWP, AP, EP, land use, energy use | Sweden | LCA based on farm data | 1 conventional and 1 organic farm. Farm data within the range of other Swedish farms | ton of energy corrected milk at the farm gate | GWP: 1 085 kg CO_2_eq  AP: 17.98 kg SO_2_eq  EP: 275 kg O_2_eq  LU: 1 925 m^2^  EU: 3 550 MJ | GWP: 949 kg CO_2_eq  AP: 15.81 kg SO_2_eq  EP: 300 kg O_2_eq  LU: 3 464 m^2^  EU: 2 511 MJ | Not tested |
| Del Prado *et al.* (2011) | GWP, biodiversity loss | United Kingdom | Whole farm simulation model and LCA (biod only on-farm) | Parameterized to simulate a farm in the Lancashire County (UK) | kg milk at the farm gate | GWP: 1.2 kg CO_2_eq | GWP: 1.0 kg CO_2_eq  Biod: improvement in biodiversity index of 5% compared to convent. | Not tested |
| Flysjö *et al.* (2012) | GWP, land use | Sweden | LCA based on farm data | 9 high yielding conventional farms and 6 organic farms | kg energy corrected milk at the farm gate | GWP: 1.07 kg CO_2_eq  LU: 1.5 m^2^ | GWP: 1.13 kg CO_2_eq  LU: 2.9 m^2^ | GWP not sign. diff (no p-value); difference of other impacts not statistically tested |
| Guerci *et al.* (2013) | GWP, AP, EP, land use, energy use, biodiversity loss | Denmark | LCA based on farm data | 3 conventional and 2 organic farms | kg energy corrected milk at the farm gate | GWP: 1.50 kg CO_2_eq  AP: 18.0 g SO_2_eq  EP: 8.15 g PO_4_^3-^eq  LU: 1.27 m^2^  EU: 3.78 MJ  Biod: 1.09 damage score | GWP: 1.27 kg CO_2_eq  AP: 15.7 g SO_2_eq  EP: 6.97 g PO_4_^3-^eq  LU: 1.75 m^2^  EU: 2.71 MJ  Biod: 0.26 damage score | Not tested |
| Hörtenhuber *et al.* (2010) | GWP, land use | Austria | LCA based on farm data | 4 conventional and 4 organic farms from 4 different regions | kg milk at the farm gate | GWP: 1.12 kg CO_2_eq  LU: 1.63 m^2^ | GWP: 1.08 kg CO_2_eq  LU: 1.76 m^2^ | Not tested |
| Kiefer *et al.* (2014) | GWP | Germany | LCA based on farm data | 81 dairy farms from southern Germany, 44% organic | kg milk at the farm gate | GWP: 1.45 kg CO_2_eq | GWP: 1.61 kg CO_2_eq | GWP higher in organic systems (p=0.014) |
| Kristensen *et al.* (2011) | GWP, land use | Denmark | LCA based on farm data | 67 commercial and specialized dairy farms; 35 conventional and 32 organic | kg energy corrected milk at the farm gate | GWP: 1.06 kg CO_2_eq  LU: 1.57 m^2^ | GWP: 1.10 kg CO_2_eq  LU: 2.05 m^2^ | GWP higher in organic systems before allocation (p<0.05) but not sign diff after allocation; land use higher in organic systems (p<0.001) |
| Mueller *et al.* (2014) | Biodiversity loss | Sweden | LCA based on farm data | 15 dairy farms from southern Sweden; 9 conventional and 6 organic | litre milk at the farm gate |  | Biod damage potential 43% of that of convent. | Loss of biodiversity lower in organic systems (p<0.05) |
| Refsgaard *et al.* (2012) | GWP, land use | Norway | LCA based on farm data | 341 conventional milk and beef meat systems; 40 conventional milk systems; 23 organic milk and beef meat systems | kg milk at the farm gate | GWP: 1.88 kg CO_2_eq  LU: 4.6 m^2^ | GWP: 1.65 kg CO_2_eq  LU: 3.8 m^2^ | Not tested |
| Thomassen *et al.* (2008) | GWP, AP, EP, land use, energy use | Netherlands | LCA based on farm data | 21 commercial dairy farms; 10 conventional and 11 organic | kg fat-and-protein-corrected milk at the farm gate | GWP: 1.4 kg CO_2_eq  AP: 9.5 kg SO_2_eq  EP: 0.11 kg NO_3_eq  LU: 1.3 m^2^  EU: 5.0 MJ | GWP: 1.5 kg CO_2_eq  AP: 10.8 kg SO_2_eq  EP: 0.07 kg NO_3_eq  LU: 1.8 m^2^  EU: 3.1 MJ | GWP and AP not sign diff; EP and energy use lower in organic systems (p<0.001); land use higher in organic systems (p<0.001) |
| Van der Werf *et al.* (2009) | GWP, AP, EP, land use, energy use | France | LCA based on farm data | 47 dairy farms from Bretagne, western France; 41 conventional and 6 organic | ton fat-and-protein-corrected milk at the farm gate | GWP: 1 037 kg CO_2_eq  AP: 7.6 kg SO_2_eq  EP: 7.1 kg PO_4_eq  LU: 1 374 m^2^  EU: 2.8 GJ | GWP: 1 082 kg CO_2_eq  AP: 6.8 kg SO_2_eq  EP: 5.0 kg PO_4_eq  LU: 2 085 m^2^  EU: 2.6 GJ | No sign diff. except for land use (higher in organic systems; p<0.01) |
| Williams *et al.* (2006) | GWP, AP, EP, land use, energy use | England and Wales | LCA based on national statistics and year books | Various data sources: yearbooks, Defra statistics, national databases, etc. No number of farms mentioned. | ton milk at the farm gate |  | Ratios org. to convent.  GWP: 1.2  AP: 1.6  EP: 1.6  LU: 1.7  EU: 0.6 | Not tested |
|  |  |  |  |  |  |  |  |  |
| *Beef* |  |  |  |  |  |  |  |  |
| Casey and Holden (2006) | GWP | Ireland | LCA based on farm data | 10 commercial farms; 5 conventional and 5 organic | kg live weight at the farm gate | GWP: 13.0 kg CO_2_eq | GWP: 11.1 kg CO_2_eq | GWP lower in organic systems (p<0.05) |
| Refsgaard *et al.* (2012) | GWP, land use | Norway | LCA based on farm data | 341 conventional milk and beef meat systems; 33 conventional beef cattle/suckler cow systems; 40 conventional milk systems; 23 organic milk and beef meat systems | kg meat at the farm gate | GWP: 16.90 kg CO_2_eq  LU: 29.8 m^2^ | GWP: 11.43 kg CO_2_eq  LU: 31.9 m^2^ | Not tested |
| Williams *et al.* (2006) | GWP, AP, EP, land use, energy use, pesticide use | England and Wales | LCA based on national statistics and year books | Various data sources: yearbooks, Defra statistics, national databases, etc. No number of farms mentioned. | ton fresh weight at the farm gate |  | Ratios org. to convent.  GWP: 1.2  AP: 1.5  EP: 2.1  LU: 1.8  EU: 0.7 | Not tested |
|  |  |  |  |  |  |  |  |  |
| *Pigs* |  |  |  |  |  |  |  |  |
| Basset-Mens and Van der Werf (2005) | GWP, AP, EP, land use, energy use | France | LCA based on national statistics (conventional) or modelling (organic) | For the conv. farm, data were from national statistics (# farms unknown); for the organic farm, data for technical performance were based on an optimized model. | kg pig live weight at the farm gate | GWP: 2.3 kg CO_2_eq  AP: 0.0435 kg SO_2_eq  EP: 0.0208 kg PO_4_eq  LU: 5.43 m^2^  EU: 15.9 MJ | GWP: 3.97 kg CO_2_eq  AP: 0.0372 kg SO_2_eq  EP: 0.0216 kg PO_4_eq  LU: 9.87 m^2^  EU: 22.2 MJ | GWP, EP, AP not sign diff.; LU, EU sign higher in organic (no p-value) |
| Dourmad *et al.* (2014) | GWP, AP, EP, land use, energy use | Denmark  Netherlands  Spain  France  Germany | LCA based on farm data | 5 to 10 conventional farms from each country; 5 to 10 organic farms from Germany and 5 to 10 from Denmark | kg pig live weight at the farm gate | GWP: 2.251 kg CO_2_eq  AP: 0.044 kg SO_2_eq  EP: 0.019 kg PO_4_eq  LU: 4.127 m^2^  EU: 16.22 MJ | GWP: 2.432 kg CO_2_eq  AP: 0.057 kg SO_2_eq  EP: 0.016 kg PO_4_eq  LU: 9.139 m^2^  EU: 18.08 MJ | No sign diff. except for land use (higher in organic systems; no p-value) |
| Van der Werf and Salou (2015) | GWP, EP, land use | France | LCA taken from Agribalyse (French data base) | National database (# farms unknown) | ton pig live weight at the farm gate | GWP: 2.4 t CO_2_eq  EP: 14 kg PO_4_eq  LU: 0.34 m^2^ | GWP: 3.5 t CO_2_eq  EP: 31 kg PO_4_eq  LU: 1.06 m^2^ | Not tested |
| Williams *et al.* (2006) | GWP, AP, EP, land use, energy use, pesticide use | England and Wales | LCA based on national statistics and year books | Various data sources: yearbooks, Defra statistics, national databases, etc. No number of farms mentioned. | ton fresh weight at the farm gate |  | Ratios org. to convent.  GWP: 0.9  AP: 0.3  EP: 0.6  LU: 1.7  EU: 0.9 | Not tested |
|  |  |  |  |  |  |  |  |  |
| *Broilers* |  |  |  |  |  |  |  |  |
| Boggia *et al.* (2010) | GWP, land use, energy use | Italy | LCA based on farm data | 1 conventional, 1 organic, and 1 organic plus farm in Central Italy | kg poultry meat at the farm gate | GWP: 1.6 E^-4^ normalised points  LU: 3.79 E^-3^ normalised points  EU: 2.01 E^-3^ normalised points | Organic (organic plus)  GWP: 1.22 (1.46) E^-4^ normalised points  LU: 7.17 (8.96) E^-3^ normalised points  EU: 1.73 (2.07) E^-3^ normalised points | Not tested |
| Castellini *et al.* (2012) | GWP, land use, energy use | Italy | LCA based on farm data | 2 conventional, 2 organic, and 2 organic plus farm central Italy | kg poultry meat at the farm gate | GWP: 250 disability adjusted life years  LU: 1.94 potentially disappeared species per m2 for year  EU: 1.68 MJ surplus | Organic (organic plus)  GWP: 180 (220) disability adjusted life years  LU: 3.68 (4.59) potentially disappeared species per m2 for year  EU: 1.45 (1.74) MJ surplus | Not tested |
| Leinonen and Kyriazakis (2013) | GWP, AP, EP, energy use | United Kingdom | Simulation model simulating a standard, organic and free range system | The model was parameterized based on data from the broiler industry (data assumed to be representative for UK) | ton expected edible carcass weight at the farm gate | Standard (free range)  GWP: 4.41 (5.13) t CO_2_eq  AP: 46.8 (59.7) kg SO_2_eq  EP: 20.3 (24.3) kg PO_4_eq  EU: 25.4 (25.7) GJ | GWP: 5.66 t CO_2_eq  AP: 91.6 kg SO_2_eq  EP: 48.8 kg PO_4_eq  EU: 40.3 GJ | GWP higher in organic compared with standard but no sign diff between organic and free range; AP, EP and energy use higher in organic compared with both standard and free range (p<0.05) |
| Van der Werf and Salou (2015) | GWP, EP, land use | France | LCA taken from Agribalyse (French data base) | National database (# farms unknown) | ton live weight at the farm gate | GWP: 2 t CO_2_eq  EP: 12 kg PO_4_eq  LU: 0.85 m^2^ | GWP: 2.3 t CO_2_eq  EP: 24 kg PO_4_eq  LU: 0.27 m^2^ | Not tested |
| Williams *et al.* (2006) | GWP, AP, EP, land use, energy use | England and Wales | LCA based on national statistics and year books | Various data sources: yearbooks, Defra statistics, national databases, etc. No number of farms mentioned. | ton fresh weight at the farm gate |  | Ratios organic to conventional  GWP: 1.5  AP: 1.5  EP: 1.8  LU: 2.2  EU: 1.3 | Not tested |
|  |  |  |  |  |  |  |  |  |
| *Laying hens* |  |  |  |  |  |  |  |  |
| Dekker *et al.* (2011) | GWP, AP, land use, energy use, | Netherlands | LCA based on data from national handbooks and expert consultation (barn and free range) or farm data (organic) | # farm of barn and free range system unknown, 20 organic farms | kg eggs at the farm gate | Barn (free range)  GWP: 2 685 (2 754) g CO_2_eq  AP: 63.5 (65.0) g SO_2_eq  LU: 3.75 (4.08) m^2^  EU: 23.2 (23.8) MJ | GWP: 2 533 g CO_2_eq  AP: 80.8 g SO_2_eq  LU: 6.76 m^2^  EU: 20.8 MJ | Not tested |
| Leinonen and Kyriazakis (2013) | GWP, AP, EP, energy use | United Kingdom | Simulation model of four systems (battery cage excl. in the current report) | The model was parameterized based on data from the egg industry (data assumed to be representative for UK) | ton marketable eggs at the farm gate | Barn (free range)  GWP: 3.45 (3.38) t CO_2_eq  AP: 59.4 (64.1) kg SO_2_eq  EP: 20.3 (22.0) kg PO_4_eq  EU: 22.2 (18.8) GJ | GWP: 3.42 t CO_2_eq  AP: 91.6 kg SO_2_eq  EP: 37.6 kg PO_4_eq  EU: 26.4 GJ | GWP not sign diff; AP, EP and energy use sign higher in organic system compared with barn and compared with free range systems (p<0.05) |
| Moudrý jr. *et al.* (2014) | GWP | Czech Republic | LCA based on farm data | 1 organic and 1 conventional farm (farms assumed to be representative for South Bohemia region) | one egg | GWP: 0.393 kg CO_2_eq | GWP: 0.219 kg CO_2_eq | Not tested |
| Williams *et al.* (2006) | GWP, AP, EP, land use, energy use | England and Wales | LCA based on national statistics and year books | Various data sources: yearbooks, Defra statistics, national databases, etc. No number of farms mentioned. | ton fresh weight at the farm gate |  | Ratios organic to conventional  GWP: 1.3  AP: 1.1  EP: 1.3  LU: 2.2  EU: 1.1 | Not tested |

^1^ Studies that used the same data as another study in this review were excluded to prevent double counting. Excluded studies on dairy: Halberg *et al.* (2005); Chen and Corson (2014). Excluded studies on pigs: Halberg *et al.* (2005); Basset-Mens *et al.* (2006); Basset-Mens *et al.* (2007); Van der Werf *et al.* (2007); Teixeira *et al.* (2013).

^2^ Including global warming potential (GWP), acidification potential (AP), eutrophication potential (EP); land use (LU), energy use (EU) and biodiversity loss (Biod); other environmental impacts addressed by a limited number of studies were not included in this review.

^3^ Some of the results presented in the studies were recalculated, e.g. to averages per system, for the purpose of this review.

**References**

Basset-Mens C and Van der Werf HMG 2005. Scenario-based environmental assessment of farming systems: the case of pig production in France. Agriculture, Ecosystems & Environment 105, 127-144.

Basset-Mens C, Werf dHMGv, Durand P and Leterme P 2006. Implications of Uncertainty and Variability in the Life Cycle Assessment of Pig Production Systems(7 pp). The International Journal of Life Cycle Assessment 11, 298-304.

Basset-Mens C, van der Werf HMG, Robin P, Morvan T, Hassouna M, Paillat JM and Vertès F 2007. Methods and data for the environmental inventory of contrasting pig production systems. Journal of Cleaner Production 15, 1395-1405.

Boggia A, Paolotti L and Castellini C 2010. Environmental impact evaluation of conventional, organic and organic-plus poultry production systems using life cycle assessment. World’s Poultry Science Journal 66, 95-114.

Capper JL, Castañeda-Gutiérrez E, Cady RA and Bauman DE 2008. The environmental impact of recombinant bovine somatotropin (rbST) use in dairy production. Proceedings of the National Academy of Sciences of the United States of America 105, 9668-9673.

Casey JW and Holden NM 2006. Greenhouse gas emissions from conventional, agri-environmental scheme, and organic Irish suckler-beef units. Journal of Environmental Quality 35, 231-239.

Castellini C, Boggia A, Cortina C, Dal Bosco A, Paolottib L, Novelli E and Mugnai C 2012. A multicriteria approach for measuring the sustainability of different poultry production systems. Journal of Cleaner Production 37, 192-201.

Cederberg C and Mattsson B 2000. Life cycle assessment of milk production - A comparison of conventional and organic farming. Journal of Cleaner Production 8, 49-60.

Chen X and Corson MS 2014. Influence of emission-factor uncertainty and farm-characteristic variability in LCA estimates of environmental impacts of French dairy farms. Journal of Cleaner Production 81, 150-157.

Dekker SEM, Aarnink AJA, De Boer IJM and Groot Koerkamp PWG 2011. Emissions of ammonia, nitrous oxide, and methane from aviaries with organic laying hen husbandry. Biosystems Engineering 110, 123-133.

Del Prado A, Misselbrook T, Chadwick D, Hopkins A, Dewhurst RJ, Davison P, Butlerd A, Schröder J and Scholefield D 2011. SIMS DAIRY: A modelling framework to identify sustainable dairy farms in the UK. Framework description and test for organic systems and N fertiliser optimisation. Science of the Total Environment 409, 3993-4009.

Dourmad JY, Ryschawy J, Trousson T, Bonneau M, Gonzalez J, Houwers HWJ, Hviid M, Zimmer C, Nguyen TLT and Morgensen L 2014. Evaluating environmental impacts of contrasting pig farming systems with life cycle assessment. Animal 8, 2027-2037.

Flysjö A, Cederberg C, Henriksson M and Ledgard S 2012. The interaction between milk and beef production and emissions from land use change - Critical considerations in life cycle assessment and carbon footprint studies of milk. Journal of Cleaner Production 28, 134-142.

Guerci M, Knudsen MT, Bava L, Zucali M, Schönbach P and Kristensen T 2013. Parameters affecting the environmental impact of a range of dairy farming systems in Denmark, Germany and Italy. Journal of Cleaner Production 54, 133-141.

Halberg N, Van Der Werf HMG, Basset-Mens C, Dalgaard R and De Boer IJM 2005. Environmental assessment tools for the evaluation and improvement of European livestock production systems. Livestock Production Science 96, 33-50.

Hörtenhuber S, Lindenthal T, Amon B, Markut T, Kirner L and Zollitsch W 2010. Greenhouse gas emissions from selected Austrian dairy production systems - Model calculations considering the effects of land use change. Renewable Agriculture and Food Systems 25, 316-329.

Kiefer L, Menzel F and Bahrs E 2014. The effect of feed demand on greenhouse gas emissions and farm profitability for organic and conventional dairy farms Journal of Dairy Science 97, 7564 – 7574.

Kristensen T, Mogensen L, Knudsen MT and Hermansen JE 2011. Effect of production system and farming strategy on greenhouse gas emissions from commercial dairy farms in a life cycle approach. Livestock Science 140, 136-148.

Leinonen I and Kyriazakis I 2013. Quantifying the environmental impacts of UK broiler and egg production systems. Lohmann Information 48, 45-50.

Moudrý jr. J, Jelínková Z, Kopecký M, Bernas J, Moudrý J, Konvalina P and Kalkuš V 2014. Emissions of greenhouse gases from the egg production within the conventional and organic farming system. Lucrări Ştiinţifice, Seria Agronomie 57, 17-21.

Mueller C, De Baan L and Koellner T 2014. Comparing direct land use impacts on biodiversity of conventional and organic milk - Based on a Swedish case study. International Journal of Life Cycle Assessment 19, 52-68.

Refsgaard K, Bergsdal H, Berglann H and Pettersen J 2012. Greenhouse gas emissions from life cycle assessment of Norwegian food production systems. Acta Agriculturae Scandinavica A: Animal Sciences 62, 336-346.

Teixeira R, Himeno A and Gustavus L 2013. Carbon footprint of breton pâté production: A case study. Integrated Environmental Assessment and Management 9, 645-651.

Thomassen MA, Van Calker KJ, Smits MCJ, Iepema GL and De Boer IJM 2008. Life cycle assessment of conventional and organic milk production in the Netherlands. Agricultural Systems 96, 95-107.

Van der Werf HMG and Salou T 2015. Economic value as a functional unit for environmental labelling of food and other consumer products. Journal of Cleaner Production Article in Press.

Van der Werf HMG, Kanyarushoki C and Corson MS 2009. An operational method for the evaluation of resource use and environmental impacts of dairy farms by life cycle assessment. Journal of Environmental Management 90, 3643-3652.

Van der Werf HMG, Tzilivakis J, Lewis K and Basset-Mens C 2007. Environmental impacts of farm scenarios according to five assessment methods. Agriculture, Ecosystems & Environment 118, 327-338.

Williams AG, Audsley E and Sandars DL 2006. Energy and environmental burdens of organic and non-organic agriculture and horticulture. Aspects of Applied Biology 79, 19-23.
