# Supplementary material for: Animal Board Invited Review: Comparing conventional and organic livestock production systems on different aspects of sustainability
Source: Animal. 2017 May 31;11(10):1839–51. doi: 10.1017/S175173111700115X (PMC5607874; doi:10.1017/S175173111700115X)
Supplement: Supplementary file 1 [file S175173111700115Xsup.zip › S175173111700115Xsup001/S175173111700115Xsup004.docx]

**Animal Board Invited review: Comparing conventional and organic livestock production systems on different aspects of sustainability**

C.P.A. van Wagenberg, Y. de Haas, H. Hogeveen, M.M. van Krimpen, M.P.M. Meuwissen, C.E. van Middelaar, T.B. Rodenburg

**Supplementary Table S4:** Reviewed studies comparing welfare indicators between conventional and organic livestock production

| Reference | Welfare indicator | Study country | Sample type | # units/samples: conventional (organic) | Unit | Value conven-tional | Value  organic | Signifi-cance | Explanation observed differences |
| --- | --- | --- | --- | --- | --- | --- | --- | --- | --- |
| *Dairy cows* |  |  |  |  |  |  |  |  |  |
| Abuelo *et al.* (2014) | 23 metabolites and immunological parameters | Spain | Blood metabolites | 1 (2) herds and 22 (40) cows | Concentration | Study gave a large number of p<0.05 differences. Overall, higher prevalence of subclinical ketosis on organic farms and higher levels of Serum amyloid A (an inflammatory agent) on conventional farms | | | |
| Abuelo *et al.* (2015) | 4 markers of oxidant production | Spain | Serum | 1 (2) herds and 22 (40) cows | Concentration | Study did not give blood values per system, only model estimates. | | | |
| Ahlman *et al.* (2011) | Longevity | Sweden | I&R records | 5 335 (402), herds | Days of productive life | 1,087 | 1,154 | - | Higher risk of culling on organic farms due to mastitis |
| Alvasen *et al.* (2012) | Mortality | Sweden | I&R records | 6 898 herds | % mortality | 61 | 55.9 | NS |  |
| Bennedsgaard *et al.* (2003) | Production diseases (Mastitis, retained placenta, ketosis) | Denmark | Milk, treatments | 99 (82) herds | Somatic cell count^1^  Mastitis treatments per cow per year  % calvings with retained placenta treatment  % calvings with ketosis treatment | 290-360  0.58-0.69  9.1-10.7  1.3-2.4 | 270-410  0.29-0.63  4-11.5  0.1-1.9 |  |  |
| Bidokhti *et al.* (2009) | Bovine coronavirus, bovine respiratory syncytial virus | Sweden | Blood ELISA | 20 (20); 699 samples from 624 cows | Prevalence:  BCV (%)  BRSV (%) | 96  91 | 78  72 | p<0.05  p<0.05 | Less animal trading between organic farms |
| Brenninkmeyer *et al.* (2013) | Hock lesions | Germany | Body condition | 33 (38) herds, 30 - 50 cows/herd | Prevalence | 68 | 22 | p<0.01 | Organic better cubicle design and lying comfort |
| Blanco-Penedo *et al.* (2014) | Metabolics (Co, Cu, Fe, I, Mn, Mo, Se, Zn) | Sweden | Blood | 10 (10) farms, 8 cows per farm, two samples in time | Concentration |  |  | NS |  |
| Blanco-Penedo *et al.* (2012a) | Metabolics (BHBA, NEFA, insulin, ketosis) | Sweden | Blood | 13 (13) farms, 81 samples | Concentration | Significant (P<0.05) lower BHBA and NEFA, not related to ketosis | | | No reason given, study was aimed at change of feeding legislation on organic farms |
| Cazer *et al.* (2013) | *Mycobacterium avium* (for Johne’s disease) | USA | Elisa test on Blood | 292 farms total  ~1/3 organic | Optical density | - | - | NS | Only final model was provided |
| Cicconi-Hogan *et al.* (2013a) | Mastitis (Somatic cell count) | USA | Bulk milk tank | 100 (192) | Concentration | 166 000 | 195 000 | NS | No difference after multivariate modelling |
| Cicconi-Hogan *et al.* (2013b) | Mastitis (*S. aureus*) | USA | Bulk milk tank | 100 (192) | Positive/negative | 42% of tanks | 67% of tanks | NS | No difference after multivariate modelling |
| Fall and Emanuelson (2009) | Mastitis, reproduction | Sweden | Milk production recording | 20 (20) | Somatic cell count concentration  Percentage success at first insemination, | -  - | -  - | NS  NS | No difference |
| Fall *et al.* (2008a) | Diseases (mastitis, ketosis, other) | Sweden | Veterinary treatments | 154 (156) cows within 1 split herd 12 years | Treatment | 192 | 198 | NS |  |
| Fall *et al.* (2008b) | Mastitis | Sweden | Milk production recording | 156 (154) cows within 1 split herd, 12 years | Somatic cell count  Percentage success at first insemination, | -  - | -  - | NS  NS | No difference |
| Fall *et al.* (2008b) | Reproduction | Sweden | Calving interval | 154 (156) cows within 1 split herd, 12 years | Calving-first insemination interval | 75 | 73 | NS |  |
| Fall *et al.* (2008c) | Metabolic status (NEFA, BHBA, Insulin, glucose, BCS) | Sweden | blood samples | 20 (20) | Concentration, level | - | - | NS | No difference |
| Fossler *et al.* (2004) | Salmonella | USA (4 states; MI, MN, NY, WI) | Cow faeces | 84 (26) farms, 5 visits, 22,417 samples | Prevalence:  herd  cow | 92.8  4.7 | 92.3  4.9 | NS  NS |  |
| Fossler *et al.* (2005a) | Salmonella | USA (4 states, MI, MN, NY, WI) | Cow faeces | 97 (32), 5 visits | Prevalence herd | Only regression modelling results were given. No raw or least square estimates were provided. Farm type was NS | | |  |
| Fossler *et al.* (2005b) | Salmonella | USA (4 states, MI, MN, NY, WI) | Calf faeces | 97 (32), 5 visits | Prevalence herd | Only regression modelling results were given. No raw or least square estimates were provided. Farm type was NS | | |  |
| Garmo *et al.* (2010) | Mastitis (Somatic cell count) | Norway | milk production recording | 25 (24) | Concentration  Days | -  377 | Lower  376 | p<0.05  NS | No explanation provided |
| Hardeng and Edge (2001) | Mastitis, ketosis, milk fever | Norway | Veterinary treatments database | 93 (31) | Incidence:  mastitis  Ketosis  Milk fever | 29%  7.8 %  12.3 % | 14%  2.8 %  7.3 % | p<0.01  p<0.01  p<0.01 | Recording bias, while no difference in Somatic cell count. Different types of treatments |
| Hoglund *et al.* (2010) | Helminths | Sweden | Bulk milk | 105 (105) herds | ODR *O. ostertagi*  Incidence *D. vivparus*  Incidence *F. hepatica* | 0.66  9%  6.7% | 0.82  18%  7.6% | p<0.001  NS  NS |  |
| Kuhnert *et al.* (2005) | *E*. *coli* STEC O157:H7 | Switzerland | Cow faeces | 60 (60) farms, 500 cows | Herd level STEC  O157:H7 | 100%  17% | 100 %  25% | NS  NS |  |
| Langford *et al.* (2009) | Diseases | United Kingdom | Questionnaire (farmer reported) | 40 (40) farms, 2 visits per farm | Yearly incidence of:  Culling  Endometritis  Cystic ovaries  Retained placenta  Lameness  Mastitis  Ketosis  Milk fever  Displaced abomasum | 26.3  10.8  6  10.4  31.9  41.6  2.3  14.9  1.8 | 19.6  6.1  5  7  36.5  30.1  2.1  7.8  1.1 | p<0.01  p<0.05  NS  NS  NS  NS  NS  p<0.05  NS | Different feeding regime and milk production level on organic farms. Note: these are farmer reported data and biased with treatment strategy |
| Langford *et al.* (2011) | Aggression feeding gate | United Kingdom | Animal behaviour | 20 (20) herds | Frequency | 30 | 36 | p<0.05 | Conventional better roughage quality: less aggression |
| Langford *et al.* (2011) | Lying post-feeding | United Kingdom | Animal behaviour | 20 (20) herds | Percentage of time spent lying | 43 | 38 | p<0.01 | Organic better leg health? Correlation lying and lameness |
| Loef *et al.* (2007) | Reproduction | Sweden | Breeding data from milk production recording database | 2,258 (170) | Calving interval  Calving – 1^st^ AI  Calving – last AI  AI/animal  Culling | 403  91  122  1 | 399  88  127  OR=0.8 | p=0.04  p<0.05  p<0.01  NS  p<0.001 | Least square means are provided.  No explanation provided |
| Mueller and Sauerwein (2010) | Mastitis | Germany | Bulk milk tank | 33 (35) farms | Somatic cell count  Cow prevalence elevated cow Somatic cell count | 205 790  36 % | 218 750  44% | NS  NS | Farms were equal despite that dry cow therapy was not provided on organic farms |
| Nauta *et al.* (2006) | Mastitis | The Netherlands | Milk production recording | 966 (404) | Somatic cell scores  calving interval Score | -  - | +50 000  - | p<0.05  NS | Dry cow therapy, deep litter stalls |
| Park *et al.* (2012) | Mastitis | USA | Milk bacteriology | 2 farms, before and after transition | Prevalence  At parturition  At drying off | 47  45 | 70  42 | 0.006  NS | Paper gives differences in text and table. Cannot be interpreted |
| Reksen *et al.* (1999) | Reproduction | Norway | Breeding data | 87 (29) farms, 3 years of data | Days open  Calving interval | 117  374 | 119  383 | NS  p<0.01 | The energy requirements might be managed less well on organic farms |
| Roesch *et al.* (2007) | Mastitis | Switzerland | California mastitis test on quarter samples | 60 (60) | Prevalence | 12-15% | 15-18% | p<0.02 | Dry cow management |
| Roesch *et al.* (2005) | Metabolic disorders | Switzerland | Blood | 60 (60), 1 000 cows | Concentration | No differences in blood parameters glucose, NEFA, BHBA | | |  |
| Rutherford *et al.* (2009) | Lameness | United Kingdom | Locomotion scores | 40 (40) matched farms (straw or cubicles), 2 or 3 visits | Prevalence during :  Autumn, straw  Autumn cubicles  Winter straw  Winter cubicles  Spring straw  Spring cubicles | 14.5  19.1  15.3  21  17.8  23.1 | 8.3  16  9  16  12.4  18 |  | In final model, significant difference for winter period (LSM: 14.2 organic and 19.9 for conventional farms). No explanation given. |
| Sato *et al.* (2005) | Mastitis, parasitic disease | USA | Bulk milk tank | 30 (30) farms | Somatic cell count  *O. ostertagi* | 285 000  ? | 263 000  ? | NS  p<0.05 | Grazing |
| Silverlås and Blanco-Penedo (2013) | *Cryptosporidium* | Sweden | Faeces | 13 (13) farms, 221 calves and 259 cows | Prevalence:  Calves  Cows | 52.3  3.8 | 44.7  3.1 | NS  NS |  |
| Stiglbauer *et al.* (2013) | Mastitis | USA | Bulk milk tank | 100 (192) samples | Somatic cell count Concentration | 210 000 | 221 000 | NS | No difference after multivariate modelling |
| Sundberg *et al.* (2009) | Mastitis | Sweden | milk production records over 7 years | 6 567 (471) herds | Monthly average somatic cell count, averaged per lactation:  Parity 1  Parity 2  Parity 3 | 55 093  71 641  93 963 | 57 760  76 322  99 959 | NS  NS  NS | Difference in raw data disappeared after correction for milk production level |
| Sundberg *et al.* (2009) | Reproduction | Sweden | Breeding data records of 7 years | 6 567 (471) herds | Calving interval:  Parity 1  Parity 2  Parity 3 | 409  401  397 | 415  408  402 | p<0.05  p<0.05  p<0.05 | Some other reproduction parameters were also significant. No explanation given |
| Thatcher *et al.* (2014) | Mastitis | New Zealand | Bulk milk tank | 1 experimental farm, split up in two herds with 51 (46) cows | Average somatic cell count over 5 years | 152 000 | 163 000 | p<0.05 | In first years, differences were significant, later not. Management on organic farms was adjusted |
| Thomsen *et al.* (2006) | Mortality | Denmark | I&R data | 6 839 herds of which 5 % organic | Risk of mortality (herd level; LSM) | 15.9 | 0 | p<0.001 | Study was aimed at mortality, not specifically at organic farming. No explanation given. |
| Thomsen *et al.* (2007) | Loser cows | Denmark | Cow observations | 40 random herds, 3 visits | Prevalence | No quantitative descriptive results of loser cows in relation to farm system were described. Organic farming had OR of 4.8 compared to conventional farms | | | No explanation provided |
| Vaarst and Bennedsgaard (2001) | Mastitis | Denmark | Bulk milk tank | 57 (27) samples | Somatic cell count | No averages nor a significance level were provided. Somatic cell count was higher on organic farms | | | Not given |
| Vaarst *et al.* (1998) | Lameness | Denmark | Sole disorders observations | 7 (6) farms, cow observations from claw trimmer | Percentage without disorders | 59% | 41% | NS |  |
| Valle *et al.* (2007) | Culling and diseases | Norway | Questionnaire | 159 (149) | Somatic cell count (* 1 000)  Calving interval  Culling rate  Mastitis treatment  Milk fever treat  Ketosis treatment  Retained placenta treatment | 118  390  43  31  5.4  6.3  2.8 | 126  388  37  17  4.8  3.4  1.8 | NS  NS  p<0.05  p<0.05  NS  p<0.05  p<0.05 | Higher activity of organic farmers in health handling  Because of the link to treatment, disease results are biased. |
| Weller and Cooper (1996) | Mastitis, lameness, vulval discharge, retained placenta, milk fever, ketosis | United Kingdom | Farmer recorded | 11 farmers, before and after conversion | Prevalence:  Mastitis  Lameness  Vulval discharge  Retained placenta  Milk fever  Ketosis | 40.5  27.9  8.5  3.8  4.9  0.4 | 45.8  24.5  5  3.3  4.9  0.5 | -  -  -  -  -  - | No significances are described. Difference in lameness due to high forage diet. |
|  |  |  |  |  |  |  |  |  |  |
| *Beef cattle* |  |  |  |  |  |  |  |  |  |
| Blanco-Penedo *et al.* (2012b) | Mastitis, reproductive disorders, abortion, podal disorders, milk fever, ketosis | Spain | Farmer reported veterinary treatments | 26 (24) farmers, farm visit, interview | Prevalence:  Mastitis  Reproductive disorders  Abortion  Podal disorders  Milk fever  Ketosis | 0.1%  0.4%  3.4%  0.1%  0  0 | 0,2%  3.8%  6.6%  3.2%  0.4%  0.2 | NS  p<0.05  NS  NS  NS  NS | Farmer reported data, so there is a bias in management and reporting. |
|  |  |  |  |  |  |  |  |  |  |
| *Pigs* |  |  |  |  |  |  |  |  |  |
| Eijck and Borgsteede (2005) | Coccidia infections | Netherlands | fecal | 9 (11) herds, 10 (10) samples | Prevalence | 67 | 91 | NS |  |
| Eijck and Borgsteede (2005) | Ascarid infections | Netherlands | fecal | 9 (11) herds, 10 (10) samples | Prevalence | 11 | 73 | p<0.05 | Conventional housing reduces the risk of worm infections |
| Knage-Rasmussen *et al.* (2014) | Lameness | Denmark | behaviour | 44 (9) herds, 30 samples | Prevalence | 24 | 5 | p<0.05 | Organic sows less lameness due to outdoor access and space |
| Millet *et al.* (2005) | Haptoglobin | Belgium | blood | Experiment: 8 (8) groups of 4 pigs | Log concentration | 0 | -0.6 | p<0.05 | Organic better ability to cope with stress |
| Millet *et al.* (2005) | Lactate | Belgium | blood | Experiment: 8 (8) groups of 4 pigs | Concentration | 7.5 | 5 | p<0.05 | Organic better ability to cope with stress |
|  |  |  |  |  |  |  |  |  |  |
| *Broilers* |  |  |  |  |  |  |  |  |  |
| Van Overbeke *et al.* (2006) | Newcastle Disease | Belgium | blood | 11 (9) flocks, 20 (20) samples | Mean antibody titers | 5 | 3 | NS |  |
| Van Overbeke *et al.* (2006) | Infectious Bursitis | Belgium | blood | 11 (9) flocks, 20 (20) samples | Mean antibody titers | 2 800 | 6 500 | p<0.001 | Timing of vaccination with regard to slaughter age organic |
| Van Overbeke *et al.* (2006) | Infectious Bronchitis | Belgium | blood | 11 (9) flocks, 20 (20) samples | Mean antibody titers | 5 000 | 1 000 | p<0.01 | Poorer respiratory health conventional (no clinical signs) |
| Tuyttens *et al.* (2008) | Acute Phase Proteins | Belgium | blood | 7 (7) flocks, 10 (10) samples | Concentration | 5.45 | 6.21 | p<0.01 | Organic better ability to cope with stress |
| Tuyttens *et al.* (2008) | Latency to lie | Belgium | behaviour test | 7 (7) flocks, 10 (10) samples | Latency time (s) | 256 | 547 | p<0.001 | Organic better leg health |
| Tuyttens *et al.* (2008) | Hock lesions | Belgium | body condition | 7 (7) flocks, 10 (10) samples | Condition (scale: 0=very good to 3=very bad) | 1.64 | 0.3 | p<0.001 | Organic better leg health, more active |
| Williams *et al.* (2013) | Hock lesions | United Kingdom | body condition | Experiment: 4 groups of 60 birds | Incidence after challenge | 45 | 22 | p<0.05 | Organic better ability to cope with stress |
| Williams *et al.* (2013) | Footpad lesions | United Kingdom | body condition | Experiment: 4 groups of 60 birds | Incidence after challenge | 32 | 2 | p<0.05 | Organic better ability to cope with stress |
|  |  |  |  |  |  |  |  |  |  |
| *Laying hens* |  |  |  |  |  |  |  |  |  |
| Jansson *et al.* (2010) | Worm infections | Sweden | fecal | 134 (35) flocks, 26 (26) samples | Prevalence | 2 | 75 | p<0.05 | Cage housing reduces risk of worm infections |

^1^ Provided are the minimum and maximum values for conventional and organic farms over the years. E.g., somatic cell count on conventional farms varied from 290 000 to 360 000 cells per cow per year and cell count on organic farms varied from 270 000 to 410 000 cells per cow per year.

**References**

Abuelo A, Hernandez J, Benedito JL and Castillo C 2014. A comparative study of the metabolic profile, insulin sensitivity and inflammatory response between organically and conventionally managed dairy cattle during the periparturient period. Animal 8, 1516-1525.

Abuelo A, Hernandez J, Benedito JL and Castillo C 2015. A pilot study to compare oxidative status between organically and conventionally managed dairy cattle during the transition period. Reproduction in Domestic Animals 50, 538-544.

Ahlman T, Berglund B, Rydhmer L and Strandberg E 2011. Culling reasons in organic and conventional dairy herds and genotype by environment interaction for longevity. Journal of Dairy Science 94, 1568-1575.

Alvasen K, Mork MJ, Sandgren CH, Thomsen PT and Emanuelson U 2012. Herd-level risk factors associated with cow mortality in Swedish dairy herds. Journal of Dairy Science 95, 4352-4362.

Bennedsgaard TW, Thamsborg SM, Vaarst M and Enevoldsen C 2003. Eleven years of organic dairy production in Denmark: herd health and production related to time of conversion and compared to conventional production. Livestock Production Science 80, 121-131.

Bidokhti MRM, Traven M, Fall N, Emanuelson U and Alenius S 2009. Reduced likelihood of bovine coronavirus and bovine respiratory syncytial virus infection on organic compared to conventional dairy farms. Veterinary Journal 182, 436-440.

Blanco-Penedo I, Fall N and Emanuelson U 2012a. Effects of turning to 100% organic feed on metabolic status of Swedish organic dairy cows. Livestock Science 143, 242-248.

Blanco-Penedo I, Lundh T, Holtenius K, Fall N and Emanuelson U 2014. The status of essential elements and associations with milk yield and the occurrence of mastitis in organic and conventional dairy herds. Livestock Science 168, 120-127.

Blanco-Penedo I, Lopez-Alonso M, Shore RF, Miranda M, Castillo C, Hernandez J and Benedito JL 2012b. Evaluation of organic, conventional and intensive beef farm systems: health, management and animal production. Animal 6, 1503-1511.

Brenninkmeyer C, Dippel S, Brinkmann J, March S, Winckler C and Knierim U 2013. Hock lesion epidemiology in cubicle housed dairy cows across two breeds, farming systems and countries. Preventive Veterinary Medicine 109, 236-245.

Cazer CL, Mitchell RM, Cicconi-Hogan KM, Gamroth M, Richert RM, Ruegg PL and Schukken YH 2013. Associations between Mycobacterium avium subsp. paratuberculosis antibodies in bulk tank milk, season of sampling and protocols for managing infected cows. BMC Veterinary Research 9.

Cicconi-Hogan KM, Gamroth M, Richert R, Ruegg PL, Stiglbauer KE and Schukken YH 2013a. Associations of risk factors with somatic cell count in bulk tank milk on organic and conventional dairy farms in the United States. Journal of Dairy Science 96, 3689-3702.

Cicconi-Hogan KM, Gamroth M, Richert R, Ruegg PL, Stiglbauer KE and Schukken YH 2013b. Risk factors associated with bulk tank standard plate count, bulk tank coliform count, and the presence of Staphylococcus aureus on organic and conventional dairy farms in the United States. Journal of Dairy Science 96, 7578-7590.

Eijck IAJM and Borgsteede FHM 2005. A survey of gastrointestinal pig parasites on free-range, organic and conventional pig farms in The Netherlands. Veterinary Research Communications 29, 407-414.

Fall N and Emanuelson U 2009. Milk yield, udder health and reproductive performance in Swedish organic and conventional dairy herds. Journal of Dairy Research 76, 402-410.

Fall N, Forslund K and Emanuelson U 2008a. Reproductive performance, general health, and longevity of dairy cows at a Swedish research farm with both organic and conventional production. Livestock Science 118, 11-19.

Fall N, Emanuelson U, Martinsson K and Jonsson S 2008b. Udder health at a Swedish research farm with both organic and conventional dairy cow management. Preventive Veterinary Medicine 83, 186-195.

Fall N, Grohn YT, Forslund K, Essen-Gustafsson B, Niskanen R and Emanuelson U 2008c. An observational study on early-lactation metabolic profiles in Swedish organically and conventionally managed dairy cows. Journal of Dairy Science 91, 3983-3992.

Fossler CP, Wells SJ, Kaneene JB, Ruegg PL, Warnick LD, Bender JB, Eberly LE, Godden SM and Halbert LW 2005a. Herd-level factors associated with isolation of Salmonella in a multi-state study of conventional and organic dairy farms - I. Salmonella shedding in cows. Preventive Veterinary Medicine 70, 257-277.

Fossler CP, Wells SJ, Kaneene JB, Ruegg PL, Warnick LD, Bender JB, Eberly LE, Godden SM and Halbert LW 2005b. Herd-level factors associated with isolation of Salmonella in a multi-state study of conventional and organic dairy farms - II. Salmonella shedding in calves. Preventive Veterinary Medicine 70, 279-291.

Fossler CP, Wells SJ, Kaneene JB, Ruegg PL, Warnick LD, Bender JB, Godden SM, Halbert LW, Campbell AM and Zwald AMG 2004. Prevalence of Salmonella spp on conventional and organic dairy farms. Javma-Journal of the American Veterinary Medical Association 225, 567-573.

Garmo RT, Waage S, Sviland S, Henriksen BIF, Osteras O and Reksen O 2010. Reproductive Performance, Udder Health, and Antibiotic Resistance in Mastitis Bacteria isolated from Norwegian Red cows in Conventional and Organic Farming. Acta Veterinaria Scandinavica 52.

Hardeng F and Edge VL 2001. Mastitis, ketosis, and milk fever in 31 organic and 93 conventional Norwegian dairy herds. Journal of Dairy Science 84, 2673-2679.

Hoglund J, Dahlstrom F, Engstrom A, Hessle A, Jakubek EB, Schnieder T, Strube C and Sollenberg S 2010. Antibodies to major pasture borne helminth infections in bulk-tank milk samples from organic and nearby conventional dairy herds in south-central Sweden. Veterinary Parasitology 171, 293-299.

Jansson DS, Nyman A, Vagsholm I, Christensson D, Goransson M, Fossum O and Hoglund J 2010. Ascarid infections in laying hens kept in different housing systems. Avian Pathology 39, 525-532.

Knage-Rasmussen KM, Houe H, Rousing T and Sorensen JT 2014. Herd- and sow-related risk factors for lameness in organic and conventional sow herds. Animal 8, 121-127.

Kuhnert P, Dubosson CR, Roesch M, Homfeld E, Doherr MG and Blum JW 2005. Prevalence and risk-factor analysis of Shiga toxigenic Escherichia coli in faecal samples of organically and conventionally farmed dairy cattle. Veterinary Microbiology 109, 37-45.

Langford FM, Rutherford KMD, Jack MC, Sherwood L, Lawrence AB and Haskell MJ 2009. A comparison of management practices, farmer-perceived disease incidence and winter housing on organic and non-organic dairy farms in the UK. Journal of Dairy Research 76, 6-14.

Langford FM, Rutherford KMD, Sherwood L, Jack MC, Lawrence AB and Haskell MJ 2011. Behavior of cows during and after peak feeding time on organic and conventional dairy farms in the United Kingdom. Journal of Dairy Science 94, 746-753.

Loef E, Gustafsson H and Emanuelson U 2007. Associations between herd characteristics and reproductive efficiency in dairy herds. Journal of Dairy Science 90, 4897-4907.

Millet S, Cox E, Buyse J, Goddeeris BM and Janssens GPJ 2005. Immunocompetence of fattening pigs fed organic versus conventional diets in organic versus conventional housing. Veterinary Journal 169, 293-299.

Mueller U and Sauerwein H 2010. A comparison of somatic cell count between organic and conventional dairy cow herds in West Germany stressing dry period related changes. Livestock Science 127, 30-37.

Nauta WJ, Baars T and Bovenhuis H 2006. Converting to organic dairy farming: Consequences for production, somatic cell scores and calving interval of first parity Holstein cows. Livestock Science 99, 185-195.

Park YK, Fox LK, Hancock DD, McMahan W and Park YH 2012. Prevalence and antibiotic resistance of mastitis pathogens isolated from dairy herds transitioning to organic management. Journal of Veterinary Science 13, 103-105.

Reksen O, Tverdal A and Ropstad E 1999. A comparative study of reproductive performance in organic and conventional dairy husbandry. Journal of Dairy Science 82, 2605-2610.

Roesch M, Doherr MG and Blum JW 2005. Performance of dairy cows on Swiss farms with organic and integrated production. Journal of Dairy Science 88, 2462-2475.

Roesch M, Doherr MG, Schaeren W, Schaellibaum M and Blum JW 2007. Subclinical mastitis in dairy cows in Swiss organic and conventional production systems. Journal of Dairy Research 74, 86-92.

Rutherford KMD, Langford FM, Jack MC, Sherwood L, Lawrence AB and Haskell MJ 2009. Lameness prevalence and risk factors in organic and non-organic dairy herds in the United Kingdom. Veterinary Journal 180, 95-105.

Sato K, Bartlett PC, Erskine RJ and Kaneene JB 2005. A comparison of production and management between Wisconsin organic and conventional dairy herds. Livestock Production Science 93, 105-115.

Silverlås C and Blanco-Penedo I 2013. *Cryptosporidium* spp. in calves and cows from organic and conventional dairy herds. Epidemiology and Infection 141, 529-539.

Stiglbauer KE, Cicconi-Hogan KM, Richert R, Schukken YH, Ruegg PL and Gamroth M 2013. Assessment of herd management on organic and conventional dairy farms in the United States. Journal of Dairy Science 96, 1290-1300.

Sundberg T, Berglund B, Rydhmer L and Strandberg E 2009. Fertility, somatic cell count and milk production in Swedish organic and conventional dairy herds. Livestock Science 126, 176-182.

Thatcher A, Martin N and Petrovski KR 2014. A study of subclinical mastitis in two herds, one managed organically, the other conventionally, and the effect of different management strategies. Organic Agriculture 4, 313-317.

Thomsen PT, Ostergaard S, Houe H and Sorensen JT 2007. Loser cows in Danish dairy herds: Risk factors. Preventive Veterinary Medicine 79, 136-154.

Thomsen PT, Kjeldsen AM, Sorensen JT, Houe H and Ersboll AK 2006. Herd-level risk factors for the mortality of cows in Danish dairy herds. Veterinary Record 158, 622-626.

Tuyttens F, Heyndrickx M, De Boeck M, Moreels A, Van Nuffel A, Van Poucke E, Van Coillie E, Van Dongen S and Lens L 2008. Broiler chicken health, welfare and fluctuating asymmetry in organic versus conventional production systems. Livestock Science 113, 123-132.

Vaarst M and Bennedsgaard TW 2001. Reduced medication in organic farming with emphasis on organic dairy production. Acta Veterinaria Scandinavica, 51-57.

Vaarst M, Hindhede J and Enevoldsen C 1998. Sole disorders in conventionally managed and organic dairy herds using different housing systems. Journal of Dairy Research 65, 175-186.

Valle PS, Lien G, Flaten O, Koesling M and Ebbesvik M 2007. Herd health and health management in organic versus conventional dairy herds in Norway. Livestock Science 112, 123-132.

Van Overbeke I, Duchateau L, De Zutter L, Albers G and Ducatelle R 2006. A comparison survey of organic and conventional broiler chickens for infectious agents affecting health and food safety. Avian Diseases 50, 196-200.

Weller RF and Cooper A 1996. Health status of dairy herds converting from conventional to organic dairy fanning. Veterinary Record 139, 141-142.

Williams LK, Sait LC, Trantham EK, Cogan TA and Humphrey TJ 2013. Campylobacter Infection Has Different Outcomes in Fast- and Slow-Growing Broiler Chickens. Avian Diseases 57, 238-241.
