# Supplementary material for: Animal Board Invited Review: Comparing conventional and organic livestock production systems on different aspects of sustainability
Source: Animal. 2017 May 31;11(10):1839–51. doi: 10.1017/S175173111700115X (PMC5607874; doi:10.1017/S175173111700115X)
Supplement: Supplementary file 1 [file S175173111700115Xsup.zip › S175173111700115Xsup001/S175173111700115Xsup005.docx]

**Animal Board Invited review: Comparing conventional and organic livestock production systems on different aspects of sustainability**

C.P.A. van Wagenberg, Y. de Haas, H. Hogeveen, M.M. van Krimpen, M.P.M. Meuwissen, C.E. van Middelaar, T.B. Rodenburg

**Supplement Table S5:** Reviewed studies comparing microbiological hazards in organic and conventional livestock production

| Reference | Hazard | Study country | Sample type / sample point | # units/samples: conventional (organic) | Unit | Value conven-tional | Value organic | Significance | Explanation observed differences |
| --- | --- | --- | --- | --- | --- | --- | --- | --- | --- |
| *Dairy cattle* |  |  |  |  |  |  |  |  |  |
| Bennedsgaard *et al.* (2006) | *Staphylococcus aureus* | Denmark | quarter milk / farm | 20 (18) herds, 30 cows per herd | cow prevalence (%) | 23 | 25 | p>0.05 | not mentioned |
| Bombyk *et al.* (2008) | *Staphylococcus* | USA (Minnesota) | composite quarter milk / farm | 8 (8) farms, 339 (501) cows | sample prevalence (%) | 49 | 47.7 | p>0.05 | different profiles of S types, due to pasture, fly bites, dry cow antibiotic treatment |
| Cho *et al.* (2006a) | Shiga Toxin-encoding bacteria | USA (Minnesota) | Fecal / farm | 20 (8) farms, 1 750 (458) samples | herd prevalence (%) | 66.7 | 87.5 | p=0.37 | Housing type, pasture access, feeding practices, age differences, season, culture methods |
| Cho *et al.* (2006a) | Shiga Toxin-encoding bacteria | USA (Minnesota) | fecal / farm | 20 (8) farms, 1 750 (458) samples | sample prevalence (%) | 4 | 6.6 | p=0.06 | Housing type, pasture access, feeding practices, age differences, season, culture methods |
| Cho *et al.* (2006a) | Shiga Toxigenic *Escherichia Coli* | USA (Minnesota) | fecal / farm | 20 (8) farms, 1 750 (458) samples | virulence genes prevalence (%) | - | - | p>0.05 | Housing type, pasture access, feeding practices, age differences, season, culture methods |
| Cho *et al.* (2006b) | *Escherichia Coli* O157 | USA (Minnesota) | rectal fecal / farm | 18 (8) farms, 271 (166) samples | sample prevalence (%) | 3 | 8.4 | p=0.15 | org: smaller herds size, tie stalls, lower rolling herd average, less likely affiliated with Dairy Herd Improvement Association; general: region, season, detection method |
| Coorevits *et al.* (2008) | spore forming bacteria (*Bacillus*) | Belgium | bulk milk tank / farm | 5 (5) farms | sample prevalence (%) | 56.3 | 43.7 | p<0.01 | seasonal variation, soil ingestion (less in winter or indoor), concentrated feed |
| Čuboň *et al.* (2008) | total bacteria count | Slovakia | bulk milk tank / farm | 1 (1) farm, 10 (10) samples | 1 000 CFU/ml | 51 | 86 | p=++ | not mentioned |
| Čuboň *et al.* (2008) | coliform organisms | Slovakia | bulk milk tank / farm | 1 (1) farm, 10 (10) samples | 1 000 CFU/ml | 269 | 554 | p=+ | not mentioned |
| Garmo *et al.* (2010) | *Staphylococcus aureus* | Norway | quarter milk of mastitis cows / farm | 25 (24) herds,  2 092 (1 948) samples | sample prevalence (%) | 3.3 | 3.4 | p>0.05 | not mentioned |
| Garmo *et al.* (2010) | *Streptococcus dysgalactiae* | Norway | quarter milk of mastitis cows / farm | 25 (24) herds,  2 092 (1 948) samples | sample prevalence (%) | 1.3 | 1.7 | p>0.05 | conv: higher motivation to improve udder health and more use of dry cow therapy |
| Garmo *et al.* (2010) | *Streptococcus uberis* | Norway | quarter milk of mastitis cows / farm | 25 (24) herds,  2 092 (1 948) samples | sample prevalence (%) | 1.2 | 0.6 | p>0.05 | not mentioned |
| Garmo *et al.* (2010) | other *Streptococcus* | Norway | quarter milk of mastitis cows / farm | 25 (24) herds,  2 092 (1 948) samples | sample prevalence (%) | 0.2 | 0.3 | p>0.05 | not mentioned |
| Garmo *et al.* (2010) | *Escherichia Coli* | Norway | quarter milk of mastitis cows / farm | 25 (24) herds,  2 092 (1 948) samples | sample prevalence (%) | 0 | 0.2 | p>0.05 | not mentioned |
| Garmo *et al.* (2010) | *Enterococcus* spp. | Norway | quarter milk of mastitis cows / farm | 25 (24) herds,  2 092 (1 948) samples | sample prevalence (%) | 1.4 | 0.5 | p>0.05 | not mentioned |
| Garmo *et al.* (2010) | bacteria negative | Norway | quarter milk of mastitis cows / farm | 25 (24) herds,  2 092 (1 948) samples | sample prevalence (%) | 84.5 | 82.8 | p>0.05 | not mentioned |
| Kouřimská *et al.* (2014) | coliform bacteria count | Czech Republic | bulk milk tank / farm | 473 (101) samples | CFU/ml | 480 | 450 | p=0.682 | farm size, disinfection milking equipment |
| Kouřimská *et al.* (2014) | total mesophilic bacteria count | Czech Republic | bulk milk tank / farm | 1 168 (218) samples | 1 000 CFU/ml | 19 | 28 | p<0.001 | farm size, disinfection milking equipment |
| Kuhnert *et al.* (2005) | *Shiga Toxigenic Escherichia Coli* | Switzerland | rectal fecal / farm | 60 (60) farms, 485 (481) samples | farm prevalence (%) | 100 | 100 | not significant | not mentioned |
| Kuhnert *et al.* (2005) | *STEC* O157:H7 | Switzerland | rectal fecal / farm | 60 (60) farms, 485 (481) samples | farm prevalence (%) | 17 | 25 | not significant | not mentioned |
| Miranda *et al.* (2009a) | *Escherichia coli* | Spain | pasteurized cheese / supermarket | 67 (60) cheeses, 10 (12) samples of same brand | sample prevalence (%) | 86.6 | 71.7 | p>0.05 | heat treatment and hygiene during manufacture, packaging and handling more important than type of milk for pasteurized cheese |
| Miranda *et al.* (2009a) | *Staphylococcus aureus* | Spain | pasteurized cheese / supermarket | 67 (60) cheeses, 10 (12) samples of same brand | sample prevalence (%) | 74.6 | 83.3 | p>0.05 | heat treatment and hygiene during manufacture, packaging and handling more important than type of milk for pasteurized cheese |
| Miranda *et al.* (2009a) | *Salmonella* spp. | Spain | pasteurized cheese / supermarket | 67 (60) cheeses, 10 (12) samples of same brand | sample prevalence (%) | 97 | 100 | p>0.05 | heat treatment and hygiene during manufacture, packaging and handling more important than type of milk for pasteurized cheese |
| Miranda *et al.* (2009a) | *Listeria monocytogenes* | Spain | pasteurized cheese / supermarket | 67 (60) cheeses, 10 (12) samples of same brand | sample prevalence (%) | 98.5 | 100 | p>0.05 | heat treatment and hygiene during manufacture, packaging and handling more important than type of milk for pasteurized cheese |
| Sato *et al.* (2004a) | *Campylobacter* spp. | USA (Wisconsin) | Fecal / farm | 30 (30) neighbouring farms, 2 visits per farm | farm prevalence (%) | 29.1 | 26.7 | p=0.5253 | general: location, season, transport medium, time before processing, enrichment media, isolation method |
| Sato *et al.* (2004b) | *Staphylococcus aureus* | Denmark | bulk milk tank / farm | 20 (20) farms, 2 visits per farm | farm prevalence (%) | 85 | 50 | not provided | not mentioned |
| Sato *et al.* (2004b) | *Staphylococcus aureus* | USA (Wisconsin) | bulk milk tank / farm | 30 (30) neighbouring farms, 2 visits per farm | farm prevalence (%) | 73 | 87 | not provided | conventional farms somewhat larger |
| Sato *et al.* (2005) | *Escherichia Coli* | USA (Wisconsin) | rectal fecal / farm | 30 (30) neighbouring farms, 20 samples per farm | sample prevalence (%) | 95.8 | 92.4 | p>0.05 | not mentioned |
| Silverlås and Blanco-Penedo (2013) | *Cryptosporidium* spp. | Sweden | rectal fecal / farm | 13 (13) herds, 107 (114) calves | herd prevalence calves (%) | 52.3 | 44.7 | p>0.05 | weather conditions, attitude towards biosecurity, livestock renewal strategy |
| Silverlås and Blanco-Penedo (2013) | *Cryptosporidium* spp. | Sweden | rectal fecal / farm | 13 (13) herds, 130 (129) calves | herd prevalence cows (%) | 3.8 | 3.1 | p>0.05 | weather conditions, attitude towards biosecurity, livestock renewal strategy |
| Tikofsky *et al.* (2003) | *Staphylococcus aureus* | USA (New York, Vermont) | composite quarter milk / farm | 16 (22) herds | sample prevalence (%) | 21.86 | 15.94 | p=0.161 | not mentioned |
|  |  |  |  |  |  |  |  |  |  |
| *Beef cattle* |  |  |  |  |  |  |  |  |  |
| Blanco-Penedo *et al.* (2009) | liver condemnations | Spain | Liver / slaughter house | 3 021 (244) calves | calf prevalence (%) | 16.8 | 10.7 | p=0.000 | org: abscesses: low fraction of concentrate in ration; less crowded pens. Parasites: org: hygiene level, grazing |
| Blanco-Penedo *et al.* (2009) | lung condemnations | Spain | lung / slaughter house | 3 021 (244) calves | calf prevalence (%) | 35.2 | 23.8 | p=0.000 | conv: more crowded pens; bad indoor climate |
| Blanco-Penedo *et al.* (2009) | kidney condemnations | Spain | Kidney / slaughter house | 3 021 (244) calves | calf prevalence (%) | 11.2 | 3.7 | p=0.000 | not mentioned |
| Blanco-Penedo *et al.* (2009) | digestive tract condemnations | Spain | digestive tract / slaughter house | 3 021 (244) calves | calf prevalence (%) | 8.1 | 32 | p=0.000 | org: feeding behaviour, feed supply outdoor |
| Blanco-Penedo *et al.* (2009) | heart condemnations | Spain | heart / slaughter house | 3 021 (244) calves | calf prevalence (%) | 0.5 | 0.4 | p=0.849 | not mentioned |
| Blanco-Penedo *et al.* (2009) | leg condemnations | Spain | leg / slaughter house | 3 021 (244) calves | calf prevalence (%) | 0.2 | 0.8 | p=0.039 | not mentioned |
| Guarddon *et al.* (2014) | Mesophilic aerobic bacteria | Spain | beef steaks / supermarket | 18 supermarkets, 2 organic retail stores, 30 (30) steaks | log CFU/g | 5 | 5.9 | p<0.05 | not mentioned |
| Guarddon *et al.* (2014) | Enterobacteriaceae | Spain | beef steaks / supermarket | 18 supermarkets, 2 organic retail stores, 30 (30) steaks | log CFU/g | 3 | 3.4 | p>0.05 | not mentioned |
| Miranda *et al.* (2009b) | *Escherichia coli* | ? Spain | packaged beef / supermarket | 75 (75) packages | sample prevalence (%) | 42.7 | 48 | p=0.6227 | no difference due to contamination at slaughter houses and processing and via food handlers |
| Miranda *et al.* (2009b) | *Staphylococcus aureus* | ? Spain | packaged beef / supermarket | 75 (75) packages | sample prevalence (%) | 54.7 | 50.7 | p=0.7436 | no difference due to contamination at slaughter houses and processing and via food handlers |
| Miranda *et al.* (2009b) | *Listeria monocytogenes* | ? Spain | packaged beef / supermarket | 75 (75) packages | sample prevalence (%) | 29.3 | 36 | p=0.4862 | no difference due to contamination at slaughter houses and processing and via food handlers |
| Miranda *et al.* (2009b) | *Salmonella* spp. | ? Spain | packaged beef / supermarket | 75 (75) packages | sample prevalence (%) | 0 | 0 | n.d. | no difference due to contamination at slaughter houses and processing and via food handlers |
|  |  |  |  |  |  |  |  |  |  |
| *Pigs* |  |  |  |  |  |  |  |  |  |
| Bonde and Sørensen (2012) | *Salmonella* | Denmark | fecal / farm | 11 (11) herds, 449 (534) animals | pig prevalence (%) | 2.4 | 0.2 | p=0.13 | org: infection early in life, so no more shedding just before slaughter, more resistance |
| Bonde and Sørensen (2012) | *Salmonella* | Denmark | fecal / farm | 11 (11) herds, 449 (534) animals | pig prevalence at abattoir (%) | 4.2 | 1.9 | p=0.82 | org: infection early in life, so no more shedding just before slaughter, more resistance |
| Bonde and Sørensen (2012) | *Salmonella* | Denmark | meat juice / slaughter house | 11 (11) herds, 449 (534) animals | pig prevalence meat juice (%) | 4.2 | 7.1 | p=0.88 | org: infection early in life, so no more shedding just before slaughter, more resistance |
| Guarddon *et al.* (2014) | mesophilic aerobic bacteria | Spain | Steaks / supermarket | 18 supermarkets, 2 organic retail stores, 40 (40) steaks | log CFU/g | 4.7 | 5.1 | p>0.05 | not mentioned |
| Guarddon *et al.* (2014) | Enterobacteriaceae | Spain | Steaks / supermarket | 18 supermarkets, 2 organic retail stores, 40 (40) steaks | log CFU/g | 3 | 2.8 | p>0.05 | not mentioned |
| Hellström *et al.* (2010) | *Listeria monocytogenes* | Finland | rectal swap / farm | 10 (5) farms, 21 to 26 pigs per farm | pig prevalence (%) | 0 | 3 | p<0.01 | org: large group size (more pig-pig contact), access to outdoor, coarse feed (also between farms: treatment manure, hygiene practices, drinking from through) |
| Hellström *et al.* (2010) | *Listeria monocytogenes* | Finland | intestinal tract / slaughter house | 10 (5) farms, 21 to 26 pigs per farm | intestinal tract prevalence (%) | 0 | 3 | p<0.01 | Org: lack of proper cleaning and disinfection and good operating protocols at slaughter plant; environment in cutting plant |
| Hellström *et al.* (2010) | *Listeria monocytogenes* | Finland | Tonsil / slaughter house | 10 (5) farms, 21 to 26 pigs per farm | tonsil prevalence (%) | 12 | 47 | p<0.01 | Org: lack of proper cleaning and disinfection and good operating protocols at slaughter plant; environment in cutting plant |
| Hellström *et al.* (2010) | *Listeria monocytogenes* | Finland | pluck set / slaughter house | 10 (5) farms, 21 to 26 pigs per farm | pluck set prevalence (%) | 1 | 13 | p<0.01 | Org: lack of proper cleaning and disinfection and good operating protocols at slaughter plant; environment in cutting plant |
| Hellström *et al.* (2010) | *Listeria monocytogenes* | Finland | carcass / slaughter house | 10 (5) farms, 21 to 26 pigs per farm | carcass prevalence (%) | 0 | 2 | p<0.01 | Org: lack of proper cleaning and disinfection and good operating protocols at slaughter plant; environment in cutting plant |
| Hoogenboom *et al.* (2008) | *Salmonella* | Netherlands | fecal / farm | national (30) farms, (12 pigs per farm) | pig faeces sample prevalence (%) | 0 | 27 | similar to conventional | org: positive farms were recently switched to organic (7 of 8) and the other was a stable with piglets bought elsewhere |
| Hoogenboom *et al.* (2008) | *Campylobacter* | Netherlands | fecal / farm | national (30) farms, (12 pigs per farm) | pig faeces sample prevalence (%) | 0 | 56 | similar to conventional | not mentioned |
| Laukkanen *et al.* (2008) | *Yersinia pseudotuberculosis* | Finland | rectal swap / farm | 10 (5) farms, 21 to 26 pigs/farm | pig prevalence (%) | 3 | 19 | p<0.05 | org: more contact with pest and pet animals and outside environment; between-farm: number of pigs, drinking troughs |
| Laukkanen *et al.* (2008) | *Yersinia pseudotuberculosis* | Finland | rectal swap / farm | 10 (5) farms, 21 to 26 pigs/farm | sample prevalence (%) | 3 | 19 | not mentioned | org: more contact with pest and pet animals and outside environment; between-farm: number of pigs, drinking troughs |
| Laukkanen *et al.* (2008) | *Yersinia pseudotuberculosis* | Finland | intestinal tract / slaughter house | 10 (5) farms, 21 to 26 pigs/farm, 239 (119) swaps | intestinal tract prevalence (%) | 5 | 9 | not mentioned | org: more contact with pest and pet animals and outside environment; between-farm: number of pigs, drinking troughs |
| Laukkanen *et al.* (2008) | *Yersinia pseudotuberculosis* | Finland | tonsil / slaughter house | 10 (5) farms, 21 to 26 pigs/farm, 231 (119) swaps | tonsil prevalence (%) | 3 | 24 | not mentioned | org: more contact with pest and pet animals and outside environment; between-farm: number of pigs, drinking troughs |
| Laukkanen *et al.* (2008) | *Yersinia pseudotuberculosis* | Finland | pluck set / slaughter house | 10 (5) farms, 21 to 26 pigs/farm, 234 (120) swaps | pluck set prevalence (%) | 0.4 | 4 | not mentioned | org: more contact with pest and pet animals and outside environment; between-farm: number of pigs, drinking troughs |
| Laukkanen *et al.* (2008) | *Yersinia pseudotuberculosis* | Finland | carcass swap / slaughter house | 10 (5) farms, 21 to 26 pigs/farm, 239 (120) swaps | carcass prevalence (%) | 0 | 8 | not mentioned | org: more contact with pest and pet animals and outside environment; between-farm: number of pigs, drinking troughs |
| Miranda *et al.* (2008a) | *Escherichia coli* | Spain | Loin / supermarket | 14 (3) brands, 67 (54) loins | sample prevalence (%) | 47.8 | 64.8 | p=0.0231 | use of antimicrobial agents in conventional |
| Miranda *et al.* (2008a) | *Escherichia coli* | Spain | Loin / supermarket | 14 (3) brands, 67 (54) loins | sample prevalence with load >2 log cfu/g (%) | 3 | 16.7 | p=0.0231 | use of antimicrobial agents in conventional |
| Nowak *et al.* (2006) | *Yersinia enterocolitica* | Germany | rectal swap / farm | 6 (3) farms, 210 (200) pigs | pig prevalence (%) | 29 | 18 | p=0.014 | conv: varying piglet suppliers, commercial feed and transport to slaughterhouse; different slaughterhouses (cross-contamination risk), all slaughtered early in morning (later, more risk due to higher probability intake faeces other animals) |
| Nowak *et al.* (2006) | *Yersinia enterocolitica* | Germany | tonsil / slaughter house | 6 (3) farms, 210 (200) pigs | tonsil prevalence (%) | 22 | 11 | p=0.025 | conv: varying piglet suppliers, commercial feed and transport to slaughterhouse; different slaughterhouses (cross-contamination risk), all slaughtered early in morning (later, more risk due to higher probability intake faeces other animals) |
| Nowak *et al.* (2006) | *Yersinia enterocolitica* | Germany | Caecum / slaughter house | 6 (3) farms, 210 (200) pigs | caecal prevalence (%) | 10 | 5 | p=0.243 | conv: varying piglet suppliers, commercial feed and transport to slaughterhouse; different slaughterhouses (cross-contamination risk), all slaughtered early in morning (later, more risk due to higher probability intake faeces other animals) |
| Nowak *et al.* (2006) | *Yersinia enterocolitica* | Germany | lymph nodes / slaughter house | 6 (3) farms, 210 (200) pigs | lymph nodes prevalence (%) | 7 | 2 | p=0.049 | conv: varying piglet suppliers, commercial feed and transport to slaughterhouse; different slaughterhouses (cross-contamination risk), all slaughtered early in morning (later, more risk due to higher probability intake faeces other animals) |
| Ranta *et al.* (2010) | *Listeria monocytogenes*, *Yersinia enterocolitica*, *Yersinia pseudotuberculosis* | Finland | fecal / farm | 10 (5) farms, about 25 pigs per farm | sample prevalence (%) | - | - | small conventional less than large conventional and organic | not mentioned |
| Rutjes *et al.* (2014) | Hepatitis E virus | Netherlands | Blood / farm | 24 (42) farms, 265 (417) pigs | pig sero-prevalence (%) | 72 | 89 | p=0.04 | feed supply, org: more repetitive exposure due to housing conditions e.g. more contact frequency between pigs, more exposure to manure |
| Rutjes *et al.* (2014) | Hepatitis E virus | Netherlands | Blood / farm | 24 (42) farms, 265 (417) pigs | farm prevalence (%) | 100 | 98 | not provided | feed supply, org: more repetitive exposure due to housing conditions e.g. more contact frequency between pigs, more exposure to manure |
| Rutjes *et al.* (2014) | Hepatitis E virus | Netherlands | Blood / farm | 24 (42) farms, 265 (417) pigs | per cent farms with pig seroprevalence > 95% | 40 | 60 | not provided | feed supply, org: more repetitive exposure due to housing conditions e.g. more contact frequency between pigs, more exposure to manure |
|  |  |  |  |  |  |  |  |  |  |
| *Broilers* |  |  |  |  |  |  |  |  |  |
| Alali *et al.* (2010) | *Salmonella* spp. | USA (North Carolina) | fecal droppings / farm | 4 (3) farms from 1 company, 1 house each farm, 2 flocks per house, 15 samples per flock | fecal sample prevalence (%) | 38.8 | 5.6 | p<0.0001 | conv: salmonella contaminated feed, different breeder flocks, |
| Alali *et al.* (2010) | *Salmonella* spp. | USA (North Carolina) | Feed / farm | 4 (3) farms from 1 company, 1 house each farm, 2 flocks per house, 5 samples per flock | feed sample prevalence (%) | 27.5 | 5 | p=0.007 | conv: salmonella contaminated feed, different breeder flocks, |
| Alali *et al.* (2010) | *Salmonella* spp. | USA (North Carolina) | Water / farm | 4 (3) farms from 1 company, 1 house each farm, 2 flocks per house, 5 samples per flock | water sample prevalence (%) | 0 | 0 | no difference | conv: salmonella contaminated feed, different breeder flocks, |
| Álvarez-Fernández *et al.* (2013) | Psychotrophs (indicator for keeping quality) | Spain | Carcass / supermarket | 8 retail outlets, 30 (30) carcasses | log CFU/g skin | 4.97 | 5.73 | p<0.05 | not mentioned |
| Álvarez-Fernández *et al.* (2013) | Faecal coliforms | Spain | Carcass / supermarket | 8 retail outlets, 30 (30) carcasses | log CFU/g skin | 2.95 | 2.07 | p<0.05 | not mentioned |
| Cui *et al.* (2005) | *Salmonella* spp. | USA (Maryland) | Carcass / supermarket | 3 (3) retail stores, 61 (198) carcasses | sample prevalence (%) | 44 | 61 | not provided | not mentioned |
| Cui *et al.* (2005) | *Campylobacter* spp. | USA (Maryland) | Carcass / supermarket | 3 (3) retail stores, 61 (198) carcasses | sample prevalence (%) | 74 | 76 | not provided | not mentioned |
| Guarddon *et al.* (2014) | Mesophilic aerobic bacteria | Spain | Thighs / supermarket | 18 supermarkets, 2 organic retail stores, 30 (30) thighs | log CFU/g | 5.3 | 4.7 | p>0.05 | not mentioned |
| Guarddon *et al.* (2014) | Enterobacteriaceae | Spain | Thighs / supermarket | 18 supermarkets, 2 organic retail stores, 30 (30) thighs | log CFU/g | 3.7 | 2.8 | p<0.05 | not mentioned |
| Han *et al.* (2009) | *Campylobacter* spp. | USA (Louisiana) | Carcass / supermarket | 26 (1) retail stores, 141 (53) carcasses | sample prevalence (%) | 43.3 | 43.4 | p>0.05 | geographical region, chicken producer |
| Hardy *et al.* (2013) | Aerobic bacteria | USA (Tennessee) | whole broiler carcass / supermarket | 2 (2) brands, 50 (50) carcasses | log cfu/g | - | - | one organic brand highest, other organic brand lowest, 2 conventional brands in between | not mentioned |
| Hardy *et al.* (2013) | *Campylobacter* spp. | USA (Tennessee) | whole broiler carcass / supermarket | 2 (2) brands, 50 (50) carcasses | log cfu/g | - | - | one organic brand highest, other organic brand lowest, 2 conventional brands in between | org: longer rearing period, so more time to colonize; higher vulnerability of breed; more contact with other animals and birds |
| Hardy *et al.* (2013) | *Salmonella* spp. | USA (Tennessee) | whole broiler carcass / supermarket | 2 (2) brands, 50 (50) carcasses | sample prevalence (%) | 0 | 5 | p>0.05 | not mentioned |
| Hardy *et al.* (2013) | *Staphylococcus* spp. | USA (Tennessee) | whole broiler carcass / supermarket | 2 (2) brands, 50 (50) carcasses | log cfu/g | - | - | one organic brand highest, other organic brand lowest, 2 conventional brands in between | unclear why difference |
| Heuer *et al.* (2001) | *Campylobacter* spp. | Denmark | cloacal swap / farm | 18 (12) farms, 79 (22) flocks, 10 boilers per flock | flock prevalence (%) | 36.7 | 100 | p<0.001 | org: access to soil and water in the open, high age at slaughter (slow growing breed), other breed |
| Heuer *et al.* (2001) | *Campylobacter* spp. | Denmark | cloacal swap / farm | 18 (12) farms, 79 (22) flocks, 10 boilers per flock | broiler prevalence (%) | 60 | 65 | no significant difference | not mentioned |
| Hoogenboom *et al.* (2008) | *Campylobacter* spp. | Netherlands | Faeces / farm | national average (9) farms, national average (45) samples | farm prevalence (%) | 0 | 100 | conventional much lower | not mentioned |
| Hoogenboom *et al.* (2008) | *Campylobacter* spp. | Netherlands | Faeces / farm | national average (9) farms, national average (45) samples | sample prevalence (%) | 0 | 71.1 | conventional much lower | not mentioned |
| Lestari *et al.* (2009) | *Salmonella* spp. | USA (Louisiana) | Carcass / supermarket | 26 (1) retail stores, 141 (53) carcasses | sample prevalence (%) | 22 | 20.8 | p>0.05 | larger slaughter house less contamination, test methodology, nature of sample, location in supply chain |
| Luangtongkum *et al.* (2006) | *Campylobacter* spp. | USA (Ohio) | intestinal tract / slaughter house | 8 (5) farms, 345 (355) tracts | farm prevalence (%) | 100 | 100 | not provided | not mentioned |
| Luangtongkum *et al.* (2006) | *Campylobacter* spp. | USA (Ohio) | intestinal tract / slaughter house | 9 (5) farms, 345 (355) tracts | sample prevalence (%) | 65.8 | 89.3 | p<0.05 | org: 2 to 4 weeks older birds |
| Luangtongkum *et al.* (2006) | *Campylobacter jejuni* | USA (Ohio) | intestinal tract / slaughter house | 10 (5) farms, 345 (355) tracts | sample prevalence (%) | 63.8 | 64.5 | not provided | not mentioned |
| Luangtongkum *et al.* (2006) | *Campylobacter coli* | USA (Ohio) | intestinal tract / slaughter house | 10 (5) farms, 345 (355) tracts | sample prevalence (%) | 2 | 24.8 | not provided | not mentioned |
| Mazengia *et al.* (2014) | *Salmonella* spp. | USA (Washington state) | raw chicken packages / supermarket | 1094 (228) packages | sample prevalence (%) | 10.5 | 15.4 | p=0.0394 | sample taking, handling of poultry carcasses during slaughtering |
| Miranda *et al.* (2007) | *Enterococcus* spp. | Spain | skin-on drumsticks / supermarket | 30 (5) supermarkets, 30 (30) drumsticks | log cfu/g | 2.06 | 3.18 | p=0.0002 | org: less antibiotic use |
| Miranda *et al.* (2008b) | *Escherichia coli* | Spain | skin-on drumsticks / supermarket | 12 (5) supermarkets, 61 (55) drumsticks | sample prevalence (%) | 62.3 | 81.8 | p<0.05 | not mentioned |
| Miranda *et al.* (2008b) | *Escherichia coli* | Spain | skin-on drumsticks / supermarket | 12 (5) supermarkets, 61 (55) drumsticks | positive sample log cfu/g | 1.36 | 1.82 | p=0.0001 | not mentioned |
| Miranda *et al.* (2008b) | *Staphylococcus aureus* | Spain | skin-on drumsticks / supermarket | 12 (5) supermarkets, 61 (55) drumsticks | sample prevalence (%) | 41 | 49.1 | both p<0.05 and p>0.05 mentioned | food handlers maybe more important than contamination from farm |
| Miranda *et al.* (2008b) | *Staphylococcus aureus* | Spain | skin-on drumsticks / supermarket | 12 (5) supermarkets, 61 (55) drumsticks | positive sample log cfu/g | 0.785 | 0.942 | p=0.6917 | food handlers maybe more important than contamination from farm |
| Miranda *et al.* (2008b) | *Listeria monocytogenes* | Spain | skin-on drumsticks / supermarket | 12 (5) supermarkets, 61 (55) drumsticks | sample prevalence (%) | 57.3 | 67.3 | p>0.05 | food handlers maybe more important than contamination from farm |
| Miranda *et al.* (2008b) | *Listeria monocytogenes* | Spain | skin-on drumsticks / supermarket | 12 (5) supermarkets, 61 (55) drumsticks | positive sample log cfu/g | 2.13 | 2.15 | p=0.2756 | food handlers maybe more important than contamination from farm |
| Miranda *et al.* (2008c) | Enterobacteriaceae | Spain | skin-on drumsticks / supermarket | 30 (5) supermarkets, 30 (30) drumsticks | log cfu/g | 2.66 | 3.81 | p<0.0001 | special characteristics of organic farming |
| Mollenkopf *et al.* (2014) | *Salmonella* spp. | USA (Ohio, Michigan, Pennsylvania) | chicken breast / supermarket | 27 processing plants, 17 store chains, 99 stores, 95 (40) breasts | sample prevalence (%) | 25 | 18 | no differences | origin contamination hatchery, parent stock, management slaughter/processing plant |
| Mollenkopf *et al.* (2014) | *Campylobacter* spp. | USA (Ohio, Michigan, Pennsylvania) | chicken breast / supermarket | 27 processing plants, 17 store chains, 99 stores, 95 (40) breasts | sample prevalence (%) | 13 | 5 | no differences | origin contamination hatchery, parent stock, management slaughter/processing plant |
| Pieskus *et al.* (2008) | *Salmonella* spp. | Netherlands | dust, litter, water caecum / farm | 18 (108) flocks, 771 (439) samples | flock prevalence (%) | 11 | 3.7 | not provided | org: slow growing, so at slaughter shedding below detection |
| Pieskus *et al.* (2008) | *Salmonella* spp. | Italy | dust, litter, water caecum / farm | 10 (11) flocks, 110 (100) samples | flock prevalence (%) | 20 | 18.1 | not provided | not provided |
| Rosenquist *et al.* (2013) | *Campylobacter* spp. | Denmark | carcass after chilling / processing | 228 (52) flocks, 228 (208) carcasses | carcass prevalence (%) | 19.7 | 54.2 | significant | org: earlier exposure through outdoor environment, so less shedding at slaughter |
| Rosenquist *et al.* (2013) | *Campylobacter* spp. | Denmark | carcass after chilling / processing | 228 (52) flocks, 228 (208) carcasses | mean concentration on positive carcasses (log(10) cfu/g) | 2.1 | 2 | p=0.428 | org: earlier exposure through outdoor environment, so less shedding at slaughter |
| Sapkota *et al.* (2014) | *Salmonella* spp. | USA (Mid-Atlantic states) | litter, water, feed / farm | 5 (5) farms, 2 houses each farm, 3/2/1 litter/water/feed samples per house | poultry house prevalence (%) | 30 | 80 | p=0.03 | different states, farm management, feed practices and season; org.: relatively high density |
| Sapkota *et al.* (2014) | *Salmonella* spp. | USA (Mid-Atlantic states) | litter, water, feed / farm | 5 (5) farms, 2 houses each farm, 3/2/1 litter/water/feed samples per house | farm prevalence (%) | 40 | 100 | not provided | different states, farm management, feed practices and season; org.: relatively high density |
| Sapkota *et al.* (2014) | Enterococcus spp. | USA (Mid-Atlantic states) | litter, water, feed / farm | 5 (5) farms, 2 houses each farm, 3/2/1 litter/water/feed samples per house | poultry house prevalence (%) | 100 | 100 | no difference | not mentioned |
| Van Overbeke *et al.* (2006) | *Salmonella* spp. | Belgium | hatching papers, overshoes / farm | 11 (9) farms from 1 integration | flock prevalence (%) | 0 | 0 | no significant difference | org: higher: outdoor access, less use antimicrobials; lower: older slaughter, less stress for animals, higher resistance because older at challenge |
| Van Overbeke *et al.* (2006) | *Campylobacter* spp. | Belgium | hatching papers, cecal droppings / farm | 11 (9) farms from 1 integration | flock prevalence (%) | 0 | 0 | no significant difference | org: higher: exposure soil/water in outdoor environment, longer rearing period, more susceptible breed |
| Van Overbeke *et al.* (2006) | *Salmonella* spp. | Belgium | gastrointestinal tract / slaughter house | 11 (9) farms from 1 integration, 30 (30) broilers | gastrointestinal tract prevalence (%) | 0 | 0 | no significant difference | org: higher: outdoor access, less use antimicrobials; lower: older slaughter, less stress for animals, higher resistance because older at challenge |
| Van Overbeke *et al.* (2006) | *Campylobacter* spp. | Belgium | gastrointestinal tract / slaughter house | 11 (9) farms from 1 integration, 30 (30) broilers | cecum prevalence (%) | 28 | 75 | p=0.024 | org: higher: exposure soil/water in outdoor environment, longer rearing period, more susceptible breed |
| Van Overbeke *et al.* (2006) | *Campylobacter* spp. | Belgium | gastrointestinal tract / slaughter house | 11 (9) farms from 1 integration, 30 (30) broilers | duodenum prevalence (%) | 18 | 75 | p=0.036 | org: higher: exposure soil/water in outdoor environment, longer rearing period, more susceptible breed |
|  |  |  |  |  |  |  |  |  |  |
| *Laying hens (hen)* |  |  |  |  |  |  |  |  |  |
| Álvarez-Fernández *et al.* (2012) | aerobic bacteria | Spain | egg shell / supermarket | 10 (10) boxes with 12 eggs, 40 (40) eggs | log cfu/square cm | 2.34 | 2.25 | p>0.05 | farm construction, management, handling in supply chain |
| Álvarez-Fernández *et al.* (2012) | Psychotrophs | Spain | egg shell / supermarket | 10 (10) boxes with 12 eggs, 40 (40) eggs | log cfu/square cm | 1.54 | 1.41 | p>0.05 | farm construction, management, handling in supply chain |
| Álvarez-Fernández *et al.* (2012) | Enterobacteriaceae | Spain | egg shell / supermarket | 10 (10) boxes with 12 eggs, 40 (40) eggs | log cfu/square cm | 0.91 | 0.9 | p>0.05 | farm construction, management, handling in supply chain |
| Álvarez-Fernández *et al.* (2012) | coliforms | Spain | egg shell / supermarket | 10 (10) boxes with 12 eggs, 40 (40) eggs | log cfu/square cm | 0.1 | 0.25 | p>0.05 | farm construction, management, handling in supply chain |
| Álvarez-Fernández *et al.* (2012) | *Pseudomonas* spp. | Spain | egg shell / supermarket | 10 (10) boxes with 12 eggs, 40 (40) eggs | log cfu/square cm | 1.94 | 1.49 | p>0.05 | farm construction, management, handling in supply chain |
| Álvarez-Fernández *et al.* (2012) | *Enterococcus* spp. | Spain | egg shell / supermarket | 10 (10) boxes with 12 eggs, 40 (40) eggs | log cfu/square cm | 0.13 | 0.27 | p>0.05 | farm construction, management, handling in supply chain |
| Álvarez-Fernández *et al.* (2012) | *Staphylococcus* spp. | Spain | egg shell / supermarket | 10 (10) boxes with 12 eggs, 40 (40) eggs | log cfu/square cm | 2.14 | 1.36 | p<0.05 | farm construction, management, handling in supply chain |
| Álvarez-Fernández *et al.* (2012) | Moulds and yeasts | Spain | egg shell / supermarket | 10 (10) boxes with 12 eggs, 40 (40) eggs | log cfu/square cm | 1.02 | 1.3 | p>0.05 | farm construction, management, handling in supply chain |
| De Reu *et al.* (2006) | gram-negative bacteria | ? Belgium | egg shell / farm | 2 (1) farm, 40 (40) eggs | log cfu/egg shell | 3.85 | 3.31 | p<0.001 | not mentioned |
| De Reu *et al.* (2006) | total aerobic bacteria | ? Belgium | egg shell / farm | 2 (1) farm, 40 (40) eggs | log cfu/egg shell | 5.08 | 5.46 | p<0.001 | not mentioned |
| Galiş *et al.* (2011) | total microorganisms on shell | Romania | Eggs / local market | 64 (64) eggs | cfu/g | 50.9-106 | 111.4 | not provided | org: contact with environment (laying on soil, eating insects/worms/vegetation) |
| Galiş *et al.* (2011) | total microorganisms in yolk | Romania | Eggs / local market | 64 (64) eggs | cfu/g | 7.12-15.14 | 23.83 | not provided | org: contact with environment (laying on soil, eating insects/worms/vegetation) |
| Galiş *et al.* (2011) | total microorganisms in albumen | Romania | Eggs / local market | 64 (64) eggs | cfu/g | 1.36-31.47 | 51.76 | not provided | org: contact with environment (laying on soil, eating insects/worms/vegetation) |
| Galiş *et al.* (2011) | *Salmonella* spp. | Romania | Eggs / local market | 64 (64) eggs | sample prevalence (%) | 6-19 | 20-23 | not provided | conv: stricter hygiene control compared to other systems |
| Messens *et al.* (2007) | *Salmonella enterica*, *Salmonella enteritidis* | ? Belgium | commercially available eggs / farm | not mentioned | egg shell penetration | 0 | 0 | not traceable to housing system | older hens lower penetration, moulting, feed composition, breed |
| Schwaiger *et al.* (2008) | *Salmonella* spp. | Germany | cloacal swap / farm | 10 (10) farms, 400 (399) swaps | cloacal swab prevalence (%) | 1.8 | 3.5 | not statistically significant | not mentioned |
| Schwaiger *et al.* (2008) | *Campylobacter* spp. | Germany | cloacal swap / farm | 10 (10) farms, 400 (399) swaps | cloacal swab prevalence (%) | 29 | 34.8 | marginally higher | org analysed within 72 hours, conventional in up to 5 days, which could have led to conspicuous decrease in isolation rate |
| Schwaiger *et al.* (2008) | *Escherichia coli* spp. | Germany | cloacal swap / farm | 10 (10) farms, 400 (399) swaps | cloacal swab prevalence (%) | 69 | 64.4 | no relevant difference | not mentioned |
| Schwaiger *et al.* (2008) | *Citrobacter*, *Enterobacter*, Pantoea | Germany | cloacal swap / farm | 10 (10) farms, 400 (399) swaps | cloacal swab prevalence (%) | 0 | 0 | only single cases | not mentioned |
| Schwaiger *et al.* (2010) | *Enterococcus* spp. | Germany | cloacal swap / farm | 10 (10) farms, 400 (399) swaps | isolate cloacal swab prevalence (%) | 1.2025 | 1.10776942 | not provided | conv: forget to disinfect technical equipment as ventilators, lighting; org: bacteria killed by sun outdoor, lower stocking density slows bird-bird spread |
| Schwaiger *et al.* (2010) | *Listeria* spp. | Germany | cloacal swap / farm | 10 (10) farms, 400 (399) swaps | isolate cloacal swab prevalence (%) | 1.75 | 1.25313283 | not provided | conv: forget to disinfect technical equipment as ventilators, lighting; org: bacteria killed by sun outdoor, lower stocking density slows bird-bird spread |
| Schwaiger *et al.* (2010) | *Enterococcus* spp. | Germany | egg content / farm | 10 (10) farms, 40 (40) eggs | isolate egg content prevalence (%) | 27.5 | 20 | not provided | direct contact with dust, soil and faeces in house, cross-contamination at packaging |
| Schwaiger *et al.* (2010) | *Enterococcus* spp. | Germany | egg shell / farm | 10 (10) farms, 40 (40) eggs | isolate eggshell prevalence (%) | 60 | 60 | not provided | direct contact with dust, soil and faeces in house, cross-contamination at packaging |
| Schwaiger *et al.* (2010) | *Listeria* spp. | Germany | egg content / farm | 10 (10) farms, 40 (40) eggs | isolate egg content prevalence (%) | 2.5 | 0 | not provided | not mentioned |
| Schwaiger *et al.* (2010) | *Listeria* spp. | Germany | egg shell / farm | 10 (10) farms, 40 (40) eggs | isolate eggshell prevalence (%) | 0 | 0 | not provided | not mentioned |

**References**

Alali WQ, Thakur S, Berghaus RD, Martin MP and Gebreyes WA 2010. Prevalence and distribution of Salmonella in organic and conventional broiler poultry farms. Foodborne Pathogens & Disease 7, 1363-1371.

Álvarez-Fernández E, Domínguez-Rodríguez J, Capita R and Alonso-Calleja C 2012. Influence of housing systems on microbial load and antimicrobial resistance patterns of *Escherichia coli* isolates from eggs produced for human consumption. Journal of Food Protection 75, 847-853.

Álvarez-Fernández E, Cancelo A, Diaz-Vega C, Capita R and Alonso-Calleja C 2013. Antimicrobial resistance in *E. coli* isolates from conventionally and organically reared poultry: A comparison of agar disc diffusion and Sensi Test Gram-negative methods. Food Control 30, 227-234.

Bennedsgaard TW, Thamsborg SM, Aarestrup FM, Enevoldsen C, Vaarst M and Christoffersen AB 2006. Resistance to penicillin of Staphylococcus aureus isolates from cows with high somatic cell counts in organic and conventional dairy herds in Denmark. Acta Veterinaria Scandinavica 48.

Blanco-Penedo I, López-Alonso M, Shore RF, Miranda M, Castillo C, Hernández J and Benedito JL 2009. Evaluation of food safety and quality in organic beef cattle in NW Spain; a comparison with intensive and conventional systems. Agronomy Research 7, 585-591.

Bombyk RAM, Bykowski AL, Draper CE, Savelkoul EJ, Sullivan LR and Wyckoff TJO 2008. Comparison of types and antimicrobial susceptibility of Staphylococcus from conventional and organic dairies in west-central Minnesota, USA. Journal of Applied Microbiology 104, 1726-1731.

Bonde M and Sørensen JT 2012. Faecal Salmonella shedding in fattening pigs in relation to the presence of Salmonella antibodies in three pig production systems. Livestock Science 150, 236-239.

Cho S, Diez-Gonzalez F, Fossler CP, Wells SJ, Hedberg CW, Kaneene JB, Ruegg PL, Warnick LD and Bender JB 2006a. Prevalence of shiga toxin-encoding bacteria and shiga toxin-producing Escherichia coli isolates from dairy farms and county fairs. Veterinary Microbiology 118, 289-298.

Cho SB, Bender JB, Diez-Gonzalez F, Fossler CP, Hedberg CW, Kaneene JB, Ruegg PL, Warnick LD and Wells SJ 2006b. Prevalence and characterization of Escherichia coli O157 isolates from Minnesota dairy farms and county fairs. Journal of Food Protection 69, 252-259.

Coorevits A, De Jonghe V, Vandroemme J, Reekmans R, Heyrman J, Messens W, De Vos P and Heyndrickx M 2008. Comparative analysis of the diversity of aerobic spore-forming bacteria in raw milk from organic and conventional dairy farms. Systematic & Applied Microbiology 31, 126-140.

Čuboň J, Foltys V, Haščík P, Kačániová M, Ubrežiová I and Kráčmar S 2008. The raw milk quality from organic and conventional agriculture. Acta Universitatis Agriculturae et Silviculturae Mendelianae Brunensis 56, 25-30.

Cui SH, Ge BL, Zheng J and Meng JH 2005. Prevalence and antimicrobial resistance of Campylobacter spp. and Salmonella serovars in organic chickens from Maryland retail stores. Applied and Environmental Microbiology 71, 4108-4111.

De Reu K, Grijspeerdt K, Heyndrickx M, Uyttendaele M, Debevere J and Herman L 2006. Bacterial shell contamination in the egg collection chains of different housing systems for laying hens. British Poultry Science 47, 163-172.

Galiş AM, Van I, Ilie LI and Tudor L 2011. Study concerning the microbiological quality of hen eggs obtained in different breeding and exploitation systems. Lucrari Stiintifice Medicina Veterinara 44, 223-231.

Garmo RT, Waage S, Sviland S, Henriksen BIF, Osteras O and Reksen O 2010. Reproductive Performance, Udder Health, and Antibiotic Resistance in Mastitis Bacteria isolated from Norwegian Red cows in Conventional and Organic Farming. Acta Veterinaria Scandinavica 52.

Guarddon M, Miranda JM, Rodriguez JA, Vazquez BI, Cepeda A and Franco CM 2014. Quantitative detection of tetracycline-resistant microorganisms in conventional and organic beef, pork and chicken meat. CyTA Journal of Food 12, 383-388.

Han FF, Lestari SI, Pu SH and Ge BL 2009. Prevalence and antimicrobial resistance among Campylobacter spp. in Louisiana retail chickens after the enrofloxacin ban. Foodborne Pathogens and Disease 6, 163-171.

Hardy B, Crilly N, Pendleton S, Andino A, Wallis A, Zhang N and Hanning I 2013. Impact of rearing conditions on the microbiological quality of raw retail poultry meat. Journal of Food Science 78, M1232-1235.

Hellström S, Laukkanen R, Siekkinen KM, Ranta J, Maijala R and Korkeala H 2010. Listeria monocytogenes contamination in pork can originate from farms. Journal of Food Protection 73, 641-648.

Heuer OE, Pedersen K, Andersen JS and Madsen M 2001. Prevalence and antimicrobial susceptibility of thermophilic Campylobacter in organic and conventional broiler flocks. Letters in Applied Microbiology 33, 269-274.

Hoogenboom LAP, Bokhorst JG, Northolt MD, Van de Vijver LPL, Broex NJG, Mevius DJ, Meijs JAC and Van der Roest J 2008. Contaminants and microorganisms in Dutch organic food products: a comparison with conventional products. Food Additives and Contaminants Part a-Chemistry Analysis Control Exposure & Risk Assessment 25, 1195-1207.

Kouřimská L, Legarová V, Panovská Z and Pánek J 2014. Quality of Cows' Milk from Organic and Conventional Farming. Czech Journal of Food Sciences 32, 398-405.

Kuhnert P, Dubosson CR, Roesch M, Homfeld E, Doherr MG and Blum JW 2005. Prevalence and risk-factor analysis of Shiga toxigenic Escherichia coli in faecal samples of organically and conventionally farmed dairy cattle. Veterinary Microbiology 109, 37-45.

Laukkanen R, Martínez PO, Siekkinen KM, Ranta J, Maijala R and Korkeala H 2008. Transmission of *Yersinia pseudotuberculosis* in the pork production chain from farm to slaughterhouse. Applied and Environmental Microbiology 74, 5444-5450.

Lestari SI, Han FF, Wang F and Ge BL 2009. Prevalence and antimicrobial resistance of Salmonella serovars in conventional and organic chickens from Louisiana retail stores. Journal of Food Protection 72, 1165-1172.

Luangtongkum T, Morishita TY, Ison AJ, Huang SX, McDermott PF and Zhang QJ 2006. Effect of conventional and organic production practices on the prevalence and antimicrobial resistance of Campylobacter spp. in poultry. Applied and Environmental Microbiology 72, 3600-3607.

Mazengia E, Samadpour M, Hill HW, Greeson K, Tenney K, Liao G, Huang X and Meschke JS 2014. Prevalence, concentrations, and antibiotic sensitivities of Salmonella serovars in poultry from retail establishments in Seattle, Washington. Journal of Food Protection 77, 885-893.

Messens W, Grijspeerdt K, De Reu K, De Ketelaere B, Mertens K, Bamelis F, Kemps B, De Baerdemaeker J, Decuypere E and Herman L 2007. Eggshell penetration of various types of hens' eggs by Salmonella enterica serovar Enteritidis. Journal of Food Protection 70, 623-628.

Miranda JM, Vázquez BI, Fente CA, Barros-Velázquez J, Cepeda A and Abuin CMF 2008a. Antimicrobial resistance in Escherichia coli strains isolated from organic and conventional pork meat: a comparative survey. European Food Research and Technology 226, 371-375.

Miranda JM, Vázquez BI, Fente CA, Calo-Mata P, Cepeda A and Franco CM 2008b. Comparison of Antimicrobial Resistance in *Escherichia coli*, *Staphylococcus aureus*, and *Listeria monocytogenes* Strains Isolated from Organic and Conventional Poultry Meat. Journal of Food Protection 71, 2537-2542.

Miranda JM, Mondragon A, Vázquez BI, Fente CA, Cepeda A and Franco CM 2009a. Microbiological quality and antimicrobial resistance of Escherichia coli and Staphylococcus aureus isolated from conventional and organic "Arzua-Ulloa'' cheese. Cyta-Journal of Food 7, 103-110.

Miranda JM, Mondragon A, Vázquez BI, Fente CA, Cepeda A and Franco CM 2009b. Influence of farming methods on microbiological contamination and prevalence of resistance to antimicrobial drugs in isolates from beef. Meat Science 82, 284-288.

Miranda JM, Guarddon M, Mondragon A, Vázquez BI, Fente CA, Cepeda A and Franco CM 2007. Antimicrobial resistance in Enterococcus spp. strains isolated from organic chicken, conventional chicken, and turkey meat: A comparative survey. Journal of Food Protection 70, 1021-1024.

Miranda JM, Guarddon M, Vázquez BI, Fente CA, Barros-Velázquez J, Cepeda A and Franco CM 2008c. Antimicrobial resistance in Enterobacteriaceae strains isolated from organic chicken, conventional chicken and conventional turkey meat: A comparative survey. Food Control 19, 412-416.

Mollenkopf DF, Cenera JK, Bryant EM, King CA, Kashoma I, Kumar A, Funk JA, Rajashekara G and Wittum TE 2014. Organic or antibiotic-free labeling does not impact the recovery of enteric pathogens and antimicrobial-resistant Escherichia coli from fresh retail chicken. Foodborne Pathogens and Disease 11, 920-929.

Nowak B, Von Mueffling T, Caspari K and Hartung J 2006. Validation of a method for the detection of virulent Yersinia enterocolitica and their distribution in slaughter pigs from conventional and alternative housing systems. Veterinary Microbiology 117, 219-228.

Pieskus J, Kazeniauskas E, Butrimaite-Ambrozeviciene C, Stanevicius Z and Mauricas M 2008. Salmonella incidence in broiler and laying hens with the different housing systems. Journal of Poultry Science 45, 227-231.

Ranta J, Siekkinen KM, Nuotio L, Laukkanen R, Hellström S, Korkeala H and Maijala R 2010. Causal hidden variable model of pathogenic contamination from pig to pork. Statistical Modelling 10, 69-87.

Rosenquist H, Boysen L, Krogh AL, Jensen AN and Nauta M 2013. Campylobacter contamination and the relative risk of illness from organic broiler meat in comparison with conventional broiler meat. International Journal of Food Microbiology 162, 226-230.

Rutjes SA, Bouwknegt M, van der Giessen JW, De Roda Husman AM and Reusken CB 2014. Seroprevalence of hepatitis E virus in pigs from different farming systems in The Netherlands. Journal of Food Protection 77, 640-642.

Sapkota AR, Kinney EL, George A, Hulet RM, Cruz-Cano R, Schwab KJ, Zhang GY and Joseph SW 2014. Lower prevalence of antibiotic-resistant Salmonella on large-scale US conventional poultry farms that transitioned to organic practices. Science of the Total Environment 476, 387-392.

Sato K, Bartlett PC and Saeed MA 2005. Antimicrobial susceptibility of *Escherichia coli* isolates from dairy farms using organic versus conventional production methods. Javma-Journal of the American Veterinary Medical Association 226, 589-594.

Sato K, Bartlett PC, Kaneene JB and Downes FP 2004a. Comparison of prevalence and antimicrobial susceptibilities of *Campylobacter* spp. isolates from organic and conventional dairy herds in Wisconsin. Applied and Environmental Microbiology 70, 1442-1447.

Sato K, Bennedsgaard TW, Bartlett PC, Erskine RJ and Kaneene JB 2004b. Comparison of antimicrobial susceptibility of *Staphylococcus aureus* isolated from bulk tank milk in organic and conventional dairy herds in the midwestern United States and Denmark. Journal of Food Protection 67, 1104-1110.

Schwaiger K, Schmied EMV and Bauer J 2008. Comparative analysis of antibiotic resistance characteristics of Gram-negative bacteria isolated from laying hens and eggs in conventional and organic keeping systems in Bavaria, Germany. Zoonoses and Public Health 55, 331-341.

Schwaiger K, Schmied EMV and Bauer J 2010. Comparative analysis on antibiotic resistance characteristics of Listeria spp. and Enterococcus spp. isolated from laying hens and eggs in conventional and organic keeping systems in Bavaria, Germany. Zoonoses and Public Health 57, 171-180.

Silverlås C and Blanco-Penedo I 2013. *Cryptosporidium* spp. in calves and cows from organic and conventional dairy herds. Epidemiology and Infection 141, 529-539.

Tikofsky LL, Barlow JW, Santisteban C and Schukken YH 2003. A comparison of antimicrobial susceptibility patterns for Staphylococcus aureus in organic and conventional dairy herds. Microbial Drug Resistance-Mechanisms Epidemiology and Disease 9, S39-S45.

Van Overbeke I, Duchateau L, De Zutter L, Albers G and Ducatelle R 2006. A comparison survey of organic and conventional broiler chickens for infectious agents affecting health and food safety. Avian Diseases 50, 196-200.
