# Supplementary material for: Animal Board Invited Review: Comparing conventional and organic livestock production systems on different aspects of sustainability
Source: Animal. 2017 May 31;11(10):1839–51. doi: 10.1017/S175173111700115X (PMC5607874; doi:10.1017/S175173111700115X)
Supplement: Supplementary file 1 [file S175173111700115Xsup.zip › S175173111700115Xsup001/S175173111700115Xsup006.docx]

**Animal Board Invited review: Comparing conventional and organic livestock production systems on different aspects of sustainability**

C.P.A. van Wagenberg, Y. de Haas, H. Hogeveen, M.M. van Krimpen, M.P.M. Meuwissen, C.E. van Middelaar, T.B. Rodenburg

**Supplementary Table S6:** Reviewed studies comparing antibiotic resistance in organic and conventional livestock production

| Reference | Bacteria investigated | Antibiotic panel | Study country | Sample point | Sample type | # units/samples: conventional (organic) | Significantly higher ADR or MDR | Explanation observed differences |
| --- | --- | --- | --- | --- | --- | --- | --- | --- |
| *Dairy cattle* |  |  |  |  |  |  |  |  |
| Bennedsgaard *et al.* (2006) | *Staphylococcus aureus* | Penicillin | Denmark | farm | quarter milk | 20 (18) farms, 493 (391) cows | no difference between prevalence | not mentioned |
| Berge *et al.* (2010) | *Escherichia coli* | Amikacin, amoxicillin–clavulanic acid, ampicillin, cefazolin, ceftiofur, chloramphenicol, gentamicin, nalidixic acid, streptomycin, sulfisoxazole, tetracycline, trimethoprim-sulfamethoxazole | USA (California, Oregon, Washington) | farm | fecal | 11 (7) farms, 607 (345) isolates | MDR conventional odds ratio 2.58 (p=0.02) | use of antimicrobials, genetically linked resistance to more antimicrobials |
| Bombyk *et al.* (2008) | *Staphylococcus* | Erythromycin, penicillin, pirlimycin, tetracycline | USA (Minnesota) | farm | milk from teat | 8 (8) farms, 339 (501) cows | conventional: less susceptible for pirlimycin, tetracycline (p<0.044) | mechanisms behind difference remains unclear (management practices) |
| Cho *et al.* (2006) | *Escherichia coli* O157 | Amikacin, amoxicillin–clavulanic acid, ampicillin, cefazolin, cefoxitin, ceftiofur, cephalothin, chloramphenicol, enrofloxacin, gentamicin, imipenem, orbifloxacin, spectinomycin, sulfadimethoxine, tetracycline, ticarcillin, ticarcillin–clavulanic acid, trimethoprim-sulfamethoxazole | USA (Minnesota) | farm | fecal | 18 (8) farms, 271 (166) fecal samples | no differences in resistance profiles of isolates | use of antimicrobials |
| Cho *et al.* (2007) | Shiga Toxigenic *Escherichia coli* | Amikacin, amoxicillin–clavulanic acid, ampicillin, cefazolin, cefoxitin, ceftiofur, cephalothin, chloramphenicol, enrofloxacin, gentamicin, imipenem, orbifloxacin, spectinomycin, sulfadimethoxine, tetracycline, ticarcillin, ticarcillin–clavulanic acid, trimethoprim-sulfamethoxazole | USA (Minnesota) | farm | rectal fecal, milk filter | 20 (8) farms, 29 (23) isolates | conventional (spectomycin) (p<0.05)  MDR no difference | unable to compare use of antimicrobials due to too few isolates |
| Cicconi-Hogan *et al.* (2014) | coagulase-negative staphylococci | Methicillin | USA (New York, Wisconsin, Oregon) | farm | bulk milk tank | 100 (192) farms, 100 (192) samples | no difference in farm prevalence | use of antimicrobials |
| Dolejska *et al.* (2011) | ESBL-producing *Escherichia coli* |  | Czech Republic | farm | rectal swap, milk filter | 1 (1) farms, 309 (154) rectal swaps, 2 (2) milk filters | Conventional: prevalence rectal swaps 39% (<1%) conventional 1 positive milk filter (0) | use of antimicrobials, farm management practices |
| Garmo *et al.* (2010) | *Staphylococcus aureus*, coagulase-negative staphylococci | Penicillin | Norway | farm | quarter milk | 25 (24) herds, 523 (487) cows | no difference between prevalence | late indoor season higher prevalence |
| Halbert *et al.* (2006a) | *Campylobacter* spp. | Azithromycin, chloramphenicol, ciprofloxacin, clindamycin, erythromycin, gentamicin, nalidixic acid, tetracycline | USA (Michigan, Minnesota, New York, Wisconsin) | farm | fecal, bulk milk tank, milk line, water source, feed bunks, housing | total 128 farms, 912 (304) isolates | conventional: more tetracycline resistant isolates (p<0.01) | no clear relation between use of antimicrobials and resistance patterns, contact with wildlife |
| Halbert *et al.* (2006b) | *Campylobacter* spp. | Amoxicillin-clavulanic acid, ampicillin, azithromycin, ceftiofur, ceftriaxone, cephalothin, chloramphenicol, ciprofloxacin, clindamycin, erythromycin, florfenicol, gentamicin, kanamycin, nalidixic acid, streptomycin, sulfamethoxazole, tetracycline, trimethoprim-sulfamethoxazole | USA (Michigan, Minnesota, New York, Wisconsin) | farm | fecal, bulk milk tank, milk line, water source, feed bunks, housing | total 128 farms,  1 570 (460) isolates | conventional: more tetracycline resistant isolates (p=0.007) | use of antimicrobials |
| Johnston (2002) | bacteria | Penicillin g | USA (Minnesota) | farm | fecal | 5 (5) farms, 30 (30) samples, 90 (90) isolates | no difference in minimum inhibitory concentration (p=0.147) | no difference, because different bacterial isolates and large standard deviation in MIC |
| McKinney *et al.* (2010) | genes (tet(O), tet(W), sul (I), sul(II)) | Tetracycline, sulfonamide | USA (west) | farm | manure lagoon | 2 (1) farms, 63 (87) samples | conventional: 4 concentration in water solubles higher (p<0.0212), 3 concentrations in settles solids higher (p<0.0236), sul(II) no difference | use of antimicrobials |
| Miranda *et al.* (2009a) | *Escherichia coli*, *Staphylococcus aureus* | Ampicillin, aztreonam, cephalotin, chloramphenicol, cyprofloxacin, doxycycline, fosfomycin, gentamicin, nitrofurantoin, streptomycin, sulfisoxazole | Spain | retail | Arzua-Ulloa pasteurized milk-cheese | 67 (60) samples | conventional: *E. coli*: ampicillin, streptomycin (p<0.05); *S. aureus*: cephalotin, fosfomycin, gentamicin, streptomycin (p<0.05)  organic: *E. coli*: doxycycline (p<0.05); *S. aureus*: ampicillin, doxycycline, sulfisoxazole (p<0.05)  MDR: no difference in resistance patterns | use of antimicrobials, contamination by environment and meat handlers |
| Ray *et al.* (2006) | *Salmonella* spp. | Amoxicillin-clavulanic acid, ampicillin, ceftriaxone, ceftiofur, cephalothin, chloramphenicol, ciprofloxacin, gentamicin, kanamycin, nalidixic acid, streptomycin, sulfamethoxazole, tetracycline, trimethoprim-sulfamethoxazole | USA (Michigan, Minnesota, New York, Wisconsin) | farm | bulk milk tank, fecal, floors, feed bunk, manure storage, bird droppings | 69 (26) farms | conventional: at least 1 streptomycin resistant isolate (odds ratio 7.5, p<0.05), conventional more isolates resistant to streptomycin (OR 5.4) and sulfamethoxazole (OR 4.2) (p<0.05) | use of antimicrobials, previous use before conversion to organic, cross-resistance, biocide use, movement of animals, people, vehicles, wildlife between herds; herd size |
| Roesch *et al.* (2006) | *Staphylococcus aureus*, nonaureus staphylococcus spp., *Streptococcus uberis*, *Streptococcus dysgalactiae* | Amoxicillin-clavulanic acid (2:1), ceftiofur, chloramphenicol, clindamycin, enrofloxacin, eryhtromycin, gentamicin, oxacillin, penicillin, quinupristin-dalfopristin, tertracycline, vancomycin | Switzerland | farm | quarter milk | 60 (60) farms, 487 (483) cows | no difference between prevalence  MDR no difference | no explanation why no difference |
| Sato *et al.* (2004a) | *Campylobacter* | Cyprofloxacin, gentamicin, eryhtromycin, tertracylcine | USA (Wisconsin) | farm | fecal | 30 (30) farms, 300 (300) samples | No evidence for difference in resistance | no evidence for use of antimicrobials as a reason |
| Sato *et al.* (2004b) | *Staphylococcus aureus* | Bacitracin, cephapirin, chloramphenicol, ciprofloxacin, erythromycin, gentamicin, kanamycin, oxacillin, penicillin, streptomycin, sulphamethoxazole, quinupristin/dalfopristin, tetracycline, trimethoprim, vancomycin | USA (Wisconsin) | farm | bulk milk tank | USA: 30 (30) neighbouring farms, 152 (179) isolates | conventional: higher probability reduced susceptibility ciprofloxacin (OR=3.33, p<0.05) | use of antimicrobials, conventional: relatively small farm size, many organic farms in neighbourhood could have changed their philosophy regarding antimicrobials use |
| Sato *et al.* (2004b) | *Staphylococcus aureus* | Avilamycin, bacitracin, cephapirin, chloramphenicol, ciprofloxacin, erythromycin, gentamicin, kanamycin, oxacillin, penicillin, streptomycin, sulphamethoxazole, quinupristin/dalfopristin, tetracycline, trimethoprim, vancomycin | Denmark | farm | bulk milk tank | Denmark 20 (20) farms, 77 (75) isolates | organic: higher probability reduced susceptibility avilamycin (OR=0.15, p<0.05) | use of antimicrobials |
| Sato *et al.* (2005) | *Escherichia coli* | Ampicillin, amoxicillin–clavulanic acid, cephalotin, cefoxitin, ceftiofur, ceftriaxone, streptomycin, kanamycin, gentamicin, apramycin, amikacin, chloramphenicol, tetracycline, sulfamethoxazole, trimethoprim-sulfamethoxazole, nalidixic acid, cyprofloxacin | USA (Wisconsin) | farm | rectal fecal | 30 (30) farms, 595 (596) samples | conventional: ampicillin (p<0.001), streptomycin (p=0.002), kanamycin (p<0.001), gentamicin (p=0.008), chloramphenicol (p=0.003), tetracycline (p<0.001), sulfamethoxazole (p=0.021)  MDR cows no difference (p=0.434)  MDR calves conventional (p<0.001) | use of antimicrobials, preservation of resistant strains for many years |
| Tikofsky *et al.* (2003) | *Staphylococcus aureus* | Ampicillin, cephalothin, erythromycin, novobiocin, oxacillin, penicillin, penicillin-novobiocin, pirlimycin, tetracycline, vancomycin | USA (New York, Vermont) | farm | milk from teat | 16 (22) farms, 117 (144) isolates | conventional: lower susceptibility ampicillin (p=0.0007), penicillin (p=0.0106), tetracycline (p=0.00003) | use of antimicrobials (little selection pressure), mechanisms of resistance in pathogens, population of pathogens, susceptibility of a strain |
| Walk *et al.* (2007) | *Escherichia coli* | Ampicillin, amoxicillin-clavulanic acid, cephalothin, cefoxitin, ceftiofur, ceftriaxone, streptomycin, kanamycin, gentamicin, apramycin, amikacin, tetracycline, sulfamethoxazole, trimethoprim-sulfamethoxazole, nalidixic acid, ciprofloxacin | USA (Wisconsin) | farm | fecal | 30 (30) matched farms, 300 (300) samples | MDR conventional: higher | use of antimicrobials |
|  |  |  |  |  |  |  |  |  |
| *Beef cattle* |  |  |  |  |  |  |  |  |
| Guarddon *et al.* (2014) | mesophillic aerobic bacteria, *Enterobacteriaceae* | Tetracycline | Spain | retail | chicken thighs | 30 (30) beef steaks | no difference in total tetracycline-resistant bacteria counts and bacteria harbouring tet(A), tet(B) or tet(A)+tet(B) | use of antimicrobials, higher than expected in organic production |
| Miranda *et al.* (2009b) | *Escherichia coli* | Ampicillin, aztreonam, cephalotin, chloramphenicol, doxycycline, ciprofloxacin, fosfomycin, gentamycin, nitrofurantoin, streptomycin, sulfisoxazole | ? Spain | retail | pre-packaged beef steaks | 75 (75) beef steaks | conventional: ampicillin 44.8% (36.6%) (p=0.0028), doxycycline 28.7% (17.2%) (p=0.0049), gentamycin 2.3% (1.1%) (p=0.0278), sulfisoxazole 62.1% (41.9%) (p=0.034)  organic ciprofloxacin 7.5% (1.1%) (p=0.0382) | use of antimicrobials |
| Miranda *et al.* (2009b) | *Staphylococcus aureus* | Chloramphenicol, clindamycin, ciprofloxacin, doxycycline, erythromycin, gentamycin, penicillin, oxacillin, nitrofurantoin, rifampin, sulfisoxazole | ? Spain | retail | pre-packaged beef steaks | 75 (75) beef steaks | conventional: ciprofloxacin 20.8% (10.7%) (p=0.0014), doxycycline 16.7% (4.8%) (p=0.0093)  organic: gentamycin 7.1% (0.0%) (p=0.0237) | use of antimicrobials |
| Miranda *et al.* (2009b) | *Listeria monocytogenes* | Cephalotin, chloramphenicol, doxycycline, enrofloxacin, erythromycin, gentamycin, rifampin, sulfisoxazole, vancomycin | ? Spain | retail | pre-packaged beef steaks | 75 (75) beef steaks | no difference | external factors as environment or (meat) handlers in chain |
|  |  |  |  |  |  |  |  |  |
| *Pigs* |  |  |  |  |  |  |  |  |
| Garcia-Migura *et al.* (2005) | vancomycin-resistant *Enterococcus faecium* | Nitrofurantoin, penicillin, tetracycline, erythromycin, ciprofloxacin, gentamicin, streptomycin, kanamycin, quinupristin-dalfospristin, vancomycin, teicoplanin, chloramphenicol, florfenicol, bacitracin, flavomycin, salinomycin | England, Wales | farm | fecal | 7 (5) farms | MDR: traits did not appear to be specific to individual farms or sample types | use of antimicrobials, insufficient cleaning and disinfection could have allowed for persistence of VREF, new contaminated stocks, environment (domestic or wild animals, feed, litter, water) |
| Guarddon *et al.* (2014) | mesophillic aerobic bacteria, *Enterobacteriaceae* | Tetracycline | Spain | retail | pork steaks | 40 (40) samples | no difference in total tetracycline-resistant bacteria counts  conventional total count of bacteria harbouring tet(B) 3.2 log CFU/g (2.7) (p<0.05) | use of antimicrobials, higher than expected in organic production |
| Hoogenboom *et al.* (2008) | *Escherichia Coli, Enterococcus faecium, Campylobacter* spp. | Amoxicillin, cefotaxim, ciprofloxacin, chloramphenicol, gentamicin, neomycin, tertracycline, sulfamethooxazole, trimethoprim, nalidixic acid, florfenicol, linezolid, doxycycline, erythromycin, vancomycin, flavomycin, salinomycin, synercid, streptomycin, metronidazole, trimethoprim-sulfamethoxazole | Netherlands | farm | fecal | national data (31) farms, (155) samples | conventional: much higher incidence of antibiotic resistant bacteria | use of antimicrobials |
| Miranda *et al.* (2008a) | *Escherichia coli* | Ampicillin, cephalotin, chloramphenicol, doxycycline, enrofloxacin, gentamicin, nitrofurantoin, streptomycin, sulfisoxazole | Spain | retail | loin meat | 67 (54) samples | conventional: ampicillin (p<0.0001), oxycycline (p<0.0001), sulfisoxazole (p<0.0001)  MDR conventional: resistance to ≥ two agents higher (p<0.0001) | use of antimicrobials |
| Nulsen *et al.* (2008) | *Escherichia coli* | Ampicillin, gentamicin, streptomycin, tetracycline, ciprofl oxacin, cotrimoxazole, neomycin | New Zealand (North Island) | farm | fecal | 3 (1) farms | ampicillin conventional 3%, organic 0%; ciprfloxacin conventional 0%, organic 0%; co-trimoxazole conventional 11%, organic 0%; Gentamicin conventional 1%, organic 0%; neomycin conventional 1%, organic 1%; streptomiycin conventional 25%, organic 3%; tetracycline conventional 60%, organic 5% | use of antimicrobials, introduction of breeding stock with antimicrobial use history |
| Nulsen *et al.* (2008) | *Enterococcus* spp. | Ampicillin, gentamicin, streptomycin, tetracycline, vancomycin, erythromycin, virginiamycin | New Zealand (North Island) | farm | fecal | 3 (1) farms | ampicillin conventional 0%, organic 0%; erythromycin conventional 69%, organic 1%; Gentamicin conventional 0%, organic 0%; streptomiycin conventional 54%, organic 0%; tetracycline conventional 67%, organic 5%; vancomycin conventional 0%, organic 0%; virginiamycin conventional 50%, organic 0% | use of antimicrobials, introduction of breeding stock with antimicrobial use history |
|  |  |  |  |  |  |  |  |  |
| *Broilers* |  |  |  |  |  |  |  |  |
| Alali *et al.* (2010) | *Salmonella* | Ampicillin, amoxicillin/clavulanic acid, amikacin, cefoxitin, ceftriaxone, ceftiofur, cephalothin, chloramphenicol, ciprofloxacin, gentamicin, kanamycin, nalidixic acid, streptomycin, sulfamethoxazole, tetracycline, trimethoprim/sulfamethoxazole | USA (North Carolina) | farm | fecal, feed, water | 4 (3) farms from same company, 240 (180) fecal samples, 80 (60) feed samples, 80 (60) water samples | conventional: Cefoxitin 55.2% of isolates resistant (8.3%) (p=0.004), Ceftiofur 53.5% (8.3%) (p=0.004), Streptomycin 91.4% (58.3%) (p=0.01), Sulfisoxazole 25.0% (1.72%) (p=0.014)  organic: tetracycline 33.3% (6.9%) (p=0.025)  MDR conventional ≥ two antibiotics 62% (41% organic), single antibiotic 36.2% (33.3%), pan susceptible 1.7% (25%) | use of antimicrobials |
| Álvarez-Fernández *et al.* (2013) | *Escherichia coli* | Gentamicin, ampicillin, amoxicillin-clavulanic acid, piperacillin-tazobactam, cefotaxime, sulphamethoxazole-trimethoprim, chloramphenicol, tetracycline, nalidixic acid, ciprofloxacin, fosfomycin, nitrofurantoin | Spain | retail | chicken carcasses | 30 (30) carcasses | conventional: resistance prevalence gentamicin 40.0% (org. 0.0%), ampicillin 100% (53.3%), amoxicillin-clavulanic acid 73.3% (20.0%), nalidixic acid 100.0% (40.0%)  MDR conventional: average number of resistances per strain 5.20 (2.53) (p<0.05)  ciprofloxacin 73.3% (26.7%) | use of antimicrobials, co-selection for resistance, exchange resistance genes between bacteria |
| Cohen Stuart *et al.* (2012) | ESBL producing bacteria |  | Netherlands | retail | chicken breast | 12 stores, 60 (38) samples | conventional: prevalence 100% (84% organic) (p<0.001); mean load 80 (20) (p=0.001); Co-resistance rate tetracycline 73% (46%) (p<0.001) | use of antimicrobials, colonized 1-day-old chicks, cross-contamination from conventional to organic during rearing or slaughter, or from environment (soil, surface water) |
| Cui *et al.* (2005) | *Campylobacter* spp. | Chloramphnicol, ciprofloxacin, erythrmycin, tertracycline | USA (Maryland) | retail | chicken carcasses | 3 (3) stores, 61 (198) carcasses | conventional: ciprofloxacin 20% (5%) (p<0.05) | not mentioned |
| Cui *et al.* (2005) | *Salmonella* spp. | Amikacin, amoxicillin-clavulanic acid, ampicillin, apramycin, ceftiofur, ceftriaxone, cephalothin, chloramphenicol, ciprofloxacin, florfenicol, gentamicin, kanamycin, nalidixic acid, streptomycin, sulfamethoxazole, tetracycline, trimethoprim-sulfamethoxazole | USA (Maryland) | retail | chicken carcasses | 3 (3) stores, 61 (198) carcasses | MDR conventional: S. Typhimurium 100% isolates resistant 5-7 antibiotics, organic 79% isolates susceptible to all antibiotics | not mentioned |
| Garcia-Migura *et al.* (2005) | vancomycin-resistant *Enterococcus faecium* | Nitrofurantoin, penicillin, tetracycline, erythromycin, ciprofloxacin, gentamicin, streptomycin, kanamycin, quinupristin-dalfospristin, vancomycin, teicoplanin, chloramphenicol, florfenicol, bacitracin, flavomycin, salinomycin | England, Wales | farm | fecal | 6 (7) farms | MDR: traits did not appear to be specific to individual farms or sample types | use of antimicrobials, insufficient cleaning and disinfection could have allowed for persistence of VREF, new contaminated stocks, environment (domestic or wild animals, feed, litter, water) |
| Guarddon *et al.* (2014) | mesophilic aerobic bacteria, *Enterobacteriaceae* | Tetracycline | Spain | retail | chicken thighs | 30 (30) thighs | no difference in total tetracycline-resistant bacteria count  conventional: total count of bacteria harbouring tet(B) 2.8 log CFU/g (1.8) p<0.05) and tet(A)+tet(B) 3.3 log CFU/g (2.8) | use of antimicrobials |
| Han *et al.* (2009) | *Campylobacter* spp. | Ciprofloxacin, erythromycin, gentamicin, tetracycline | USA (Louisiana) | retail | chicken carcasses | 26 (1) stores, 141 (53) carcasses | conventional: ciprofloxacin (8.5% (0.0%) (p<0.05), erythromycin 23.9% (10.4%) (p<0.05) | use of antimicrobial, geographic region, chicken producer, environment |
| Heuer *et al.* (2001) | *Campylobacter* spp. | Tetracycline, ampicillin, erythromycin, enrofloxacin, streptomycin | Denmark | processing | cloacal swap | 79 (22) flocks, 790 (220) samples | antibiotic resistance scarce among isolates from all rearing systems | not established |
| Heuer *et al.* (2002) | vancomycin-resistant Enterococci | Vancomycin | Denmark | processing | cloacal swap | 24 (12) farms, 140 (22) flocks | conventional: 74.3% flock prevalence (9.1%) (p<0.0001) | use of antimicrobials, persistence in environment |
| Hoogenboom *et al.* (2008) | *Escherichia Coli, Enterococcus faecium, Campylobacter* spp. | Amoxicillin, cefotaxim, ciprofloxacin, chloramphenicol, gentamicin, neomycin, tertracycline, sulfamethooxazole, trimethoprim, nalidixic acid, florfenicol, linezolid, doxycycline, erythromycin, vancomycin, flavomycin, salinomycin, synercid, streptomycin, metronidazole, trimethoprim-sulfamethoxazole | Netherlands | farm | fecal | national data (9) farms, (45) samples | conventional: much higher incidence of antibiotic resistant E. coli and E. faecium  no difference Campylobacter | absence of selection pressure in organic animals (no use of antimicrobials) |
| Kola *et al.* (2012) | ESBL |  | Germany | retail | chicken breast and leg | 9 supermarkets, (4 organic food stores), 1 butcher | no difference | use of antimicrobials, colonized 1-day-old chicks, cross-contamination from conventional to organic during rearing or slaughter, or from environment |
| Lestari *et al.* (2009) | *Salmonella* spp. | Amikacin, amoxicillin–clavulanic acid, ampicillin, cefoxitin, ceftiofur, ceftriaxone, chloramphenicol, ciprofloxacin, gentamicin, kanamycin, nalidixic acid, streptomycin, sulfisoxazole, tetracycline, trimethoprim-sulfamethoxazole | USA (Louisiana) | retail | chicken carcasses | 26 (1) stores, 141 (53) carcasses | conventional: amoxicillin-clavulanic acid 19.4% (9.1%) (p<0.05), cefoxitin 19.4% (9.1%) (p<0.05)  organic: streptomycin 66.7% (46.2%) (p<0.05), tetracycline 63.6% (41.9%) (p<0.05)  MDR conventional 48.2% (33.3%) isolates susceptible to all antibiotics | transfer of resistant genes to other serovars |
| Luangtongkum *et al.* (2006) | *Campylobacter* spp. | Ampicillin, tetracycline, gentamicin, kanamycin, clindamycin, erythromycin, ciprofloxacin, norfloxacin, nalidixic acid | USA (Ohio) | processing | intestinal tracts | 10 (5) farms, 345 (355) tracts, 167 (165) isolates | conventional: tetracycline, ciprofloxacin, norfloxacin, nalidixic acid (p<0.05)  organic: erythromycin (p<0.05)  MDR no difference (p>0.05) | use of antimicrobials, transmission of resistant isolates without selection pressure |
| Mazengia *et al.* (2014) | *Salmonella* spp. | Amoxicillin–clavulanic acid, ampicillin, cefoxitin, ceftriaxone, chloramphenicol, ciprofloxacin, gentamicin, kanamycin, nalidixic acid, tetracycline, trimethoprim-sulfamethoxazole | USA (Washington state) | retail | raw chicken packages | total 18 stores,  1 094 (228) packages | conventional: significantly higher resistant rates than organic  MDR conventional: all isolates resistant to ≥ two antibiotics from conventional | antibiotic treatment of animals |
| Miranda *et al.* (2007) | *Enterococcus* spp. | Ampicillin, chloramphenicol, doxycycline, ciprofloxacin, erythromycin, gentamicin, nitrofurantoin, vancomycin | Spain | retail | skin-on drum stick | 30 (30) samples | conventional: higher resistance ampicillin (p=0.0067), chloramphenicol (p=0.0154), doxycycline (p=0.0277), ciprofloxacin (p=0.0024), erythromycin (p=0.0028), vancomycin (p=0.0241)  MDR conventional 33.3% (11.67%) (p=0.0021) | use of antimicrobials |
| Miranda *et al.* (2008b) | *Escherichia coli* | Ampicillin, cephalothin, chloramphenicol, doxycycline, ciprofloxacin, fosfomycin, gentamicin, nitrofurantoin, streptomycin, sulfisoxazole | Spain | retail | drum sticks | 61 (55) drum sticks | conventional: ampicillin 53.9% (21.9%) (p<0.0001), cephalothin 34.8% (4.8%) (p<0.0001), ciprofloxacin 27.8% (9.5%) (p=0.0026), doxycycline 47.8% (25.7%) (p<0.0001), gentamicin 9.6% (1%) (p<0.0001), streptomycin 46.1% (23.8%) (p<0.0001), sulfisoxazole 36.5% (21.9%) (p=0.0021)  conventional MDR 76.5% (34.3%) (p<0.0001) | use of antimicrobials |
| Miranda *et al.* (2008b) | *Staphylococcus aureus* | Chloramphenicol, doxycycline, ciprofloxacin, clindamycin, erythromycin, gentamicin, nitrofurantoin, oxacillin, sulfisoxazole | Spain | retail | drum sticks | 61 (55) drum sticks | conventional: Doxycycline 58.4% (34.1%) (p=0.0001); organic: Clindamycin 83.5% (67.3%) (p=0.0239)  MDR no difference (p=0.0826) | not mentioned |
| Miranda *et al.* (2008b) | *Listeria monocytogenes* | Chloramphenicol, doxycycline, erythromycin, gentamicin, sulfisoxazole, vancomycin | Spain | retail | drum sticks | 61 (55) drum sticks | conventional: doxycycline 18.8% (2.6%) (p=0.0446)  MDR no difference (p=0.2409) | not mentioned |
| Miranda *et al.* (2008c) | Enterobacteriaceae | Ampicillin, cephalothin, chloramphenicol, doxycycline, ciprofloxacin, gentamicin, nitrofurantoin, sulfisoxazole | Spain | retail | skin-on drum stick | 30 (30) samples | conventional: higher resistance ampicillin (p=0.0001), chloramphenicol (p=0.0004), doxycycline (p=0.0013), ciprofloxacin (p=0.0034), gentamicin (p=0.0295) and sulfisoxazole (p=0.0442)  MDR conventional 63.3% (organic 41.7%) (p=0.0197) | use of antimicrobials |
| Mollenkopf *et al.* (2014) | bla(CMY-2) *Salmonella* spp. |  | USA (Ohio, Michigan, Pennsylvania) | retail | pre-packaged chicken breasts | total 99 stores, 95 (40) packages | no difference | not mentioned |
| Mollenkopf *et al.* (2014) | bla(CMY-2), bla(CTX-M), quinolone-resistant determining regions *E. coli* |  | USA (Ohio, Michigan, Pennsylvania) | retail | pre-packaged chicken breasts | total 99 stores, 95 (40) packages | conventional: QRDR 18% (0% organic), bla(CMY-2), bla(CTX-M) no difference | not mentioned |
| Mollenkopf *et al.* (2014) | *Campylobacter* spp. | Cirpofloxacin, clindamycin, erythromycin, florfenicol, gentamicin, naladixic acid, telithromycin, tertracycline | USA (Ohio, Michigan, Pennsylvania) | retail | pre-packaged chicken breasts | total 99 stores, 95 (40) packages | no difference in proportion with increased resistance | not mentioned |
| Mollenkopf *et al.* (2014) | *Salmonella* spp. | Amoxicillin/clavulanic acid, ampicillin, azithromycin, ceftoxitin, ceftiofur, ceftriaxone, chloramphenicol, ciprofloxacin, gentamicin, kanamycin, naladixic acid, streptomycin, sulfisoxazole, tertracycline, trimethoprim-sulfamethoxazole | USA (Ohio, Michigan, Pennsylvania) | retail | pre-packaged chicken breasts | total 99 stores, 95 (40) packages | no difference in proportion with increased resistance | not mentioned |
| Mollenkopf *et al.* (2014) | *Escherichia coli* | Amoxicillin/clavulanic acid, ampicillin, azithromycin, ceftoxitin, ceftiofur, ceftriaxone, chloramphenicol, ciprofloxacin, gentamicin, kanamycin, naladixic acid, streptomycin, sulfisoxazole, tertracycline, trimethoprim-sulfamethoxazole | USA (Ohio, Michigan, Pennsylvania) | retail | pre-packaged chicken breasts | total 99 stores, 95 (40) packages | no difference in proportion with increased resistance | not mentioned |
| Sapkota *et al.* (2014) | *Salmonella* Kentucky | Amikacin, amoxicillin-clavulanate, ampicillin, cefoxitin, ceftiofur, ceftriaxone, chloramphenicol, cyprofloxacin, gentamicin, kanamycin, nalidixic acid, streptomycin, sulfisoxazole, tetracycline, sulfamethoxazole | USA (Mid-Atlantic) | farm | litter, water, feed | 5 (5) farms, 10 (10) houses, 30 (30) litter samples, 20 (20) water samples, 10 (10) feed samples | conventional isolates: amoxicillin–clavulanate (p=0.049), ampicillin (p=0.042), cefoxitin (p=0.042), ceftiofur (p=0.043), ceftriaxone (p=0.042)  MDR conventional 44% (6% organic) (p=0.015) | antibiotic selective pressure, multiple and complex factors in environment (e.g. horizontal gene transfer, changed bacterial physiology) |
| Sapkota *et al.* (2011) | *Enterococcus faecalis* | Chloramphenicol, ciprofloxacin, daptomycin, erythromycin, flavomycin, gentamicin, kanamycin, lincomycin, linezolid, nitrofurantoin, penicillin, streptomycin, quinupristin/dalfopristin, tetracycline, tigecycline, tylosin, vancomycin | USA (Mid-Atlantic) | farm | litter, water, feed | 5 (5) farms, 10 (10) houses, 30 (30) litter samples, 20 (20) water samples, 10 (10) feed samples, 133 (126) isolates | conventional: Erythromycin (p=0.004), tigecycline (p=0.004)  MDR conventional 42% (10%) (p=0.02) | use of antimicrobials, hatcheries/parent stocks use antibiotics, antibiotic-resistant bacteria contaminated feed and water |
| Sapkota *et al.* (2011) | *Enterococcus faecium* | Chloramphenicol, ciprofloxacin, daptomycin, erythromycin, flavomycin, gentamicin, kanamycin, lincomycin, linezolid, nitrofurantoin, penicillin, streptomycin, quinupristin/dalfopristin, tetracycline, tigecycline, tylosin, vancomycin | USA (Mid-Atlantic) | farm | litter, water, feed | 5 (5) farms, 10 (10) houses, 30 (30) litter samples, 20 (20) water samples, 10 (10) feed samples, 133 (126) isolates | conventional: ciprofloxacin (p=0.01), gentamicin (p=0.047), nitrofurantoin (p=0.02), penicillin (p<0.001), tetracycline (p<0.001)  MDR conventional 84% (17%) (p<0.001) | use of antimicrobials, hatcheries/parent stocks use antibiotics, antibiotic-resistant bacteria contaminated feed and water |
|  |  |  |  |  |  |  |  |  |
| *Laying hens* |  |  |  |  |  |  |  |  |
| Álvarez-Fernández *et al.* (2012) | *Escherichia coli* | Gentamicin, ampicillin-sulbactam, amoxicillin–clavulanic acid, piperacillin-tazobactam, cefotaxime, sulfamethoxazole-trimethoprim, chloramphenicol, ciprofloxacin, nalidixic acid, tetracycline, nitrofurantoin, phosphomycin | Spain | retail | eggs shell | different supermarkets, total 50 samples of 12 eggs, 20 (20) isolates | conventional: amoxicillin–clavulanic acid 90% (20%) (p<0.05), sulfamethoxazole-trimethoprim 85% (15%) (p<0.05), tetracycline 95% (0%) (p<0.05)  organic: phosphomycin 50% (0%) (p<0.05)  MDR conventional: resistant ≥ 2 antimicrobials 95% (30%) (p<0.05) | use of antimicrobials, animal crowding, poor sanitation |
| Schwaiger *et al.* (2008) | *Escherichia coli* | Amoxicillin/clavulanic acid, ampicillin, mezlocillin, oxazillin, piperacillin, cefaclor, cefepime, cefotaxime, cefoxitin, ceftazidime, ceftiofur, cefuroxime, imipenem, meropenem, chloramphenicol, florfenicol, ciprofloxacin, enrofloxacin, amikacin, apramycin, gentamicin, netilmicin, streptomycin, tobramycin, sulphamethoxazole/trimethoprim, doxycycline, colistin | Germany | farm | cloacal swap | 10 (10) farms, 276 (257) isolates | conventional: resistant to amoxicillin/clavulanic acid 11.2% (3.5%) (p<0.05), ampicillin 21.4% (9.3%) (p<0.05), cefaclor 19.6% (4.3%) (p<0.05), cefuroxime 2.6% (0.0%) (p<0.05), mezlocillin 16.7% (7.8%) (p<0.05), neomycin 5.8% (0.4%) (p<0.05), piperacillin 15.9% (2.7%) (p<0.05)  organic: gentamicin 8.6% (1.5%) (p<0.05)  MDR conventional: more double resistant isolates 10.1 (5.1 organic) (p<0.05), less susceptible to all agents 44.9% (60.7% organic) p<0.05 | use of antimicrobials, long duration of resistant population |
| Schwaiger *et al.* (2008) | *Campylobacter coli* | Amoxicillin/clavulanic acid, ampicillin, mezlocillin, oxazillin, piperacillin+tazobactam, cefuroxime, imipenem, chloramphenicol, florfenicol, ciprofloxacin, enrofloxacin, moxifloxacin, gentamicin, kanamycin, neomycin, streptomycin high level, erythromycin, tylosin, clindamycin, linezolid, sulphamethoxazole/trimethoprim, doxycycline, fosfomycin, nitrofurantoin | Germany | farm | cloacal swap | 10 (10) farms, 18 (25) isolates | no difference  MDR no statistical analysis due to low power | natural selection of resistant isolates, cross-contamination with other animals and humans, other selectors as heavy metals |
| Schwaiger *et al.* (2008) | *Campylobacter jejuni* | Amoxicillin/clavulanic acid, ampicillin, mezlocillin, oxazillin, piperacillin+tazobactam, cefuroxime, imipenem, chloramphenicol, florfenicol, ciprofloxacin, enrofloxacin, moxifloxacin, gentamicin, kanamycin, neomycin, streptomycin high level, erythromycin, tylosin, clindamycin, linezolid, sulphamethoxazole/trimethoprim, doxycycline, fosfomycin, nitrofurantoin | Germany | farm | cloacal swap | 10 (10) farms, 99 (118) isolates | conventional: resistant to amoxicillin/clavulanic acid 14.1% (4.2%) (p<0.05), imipenem 19.2% (8.5%) (p<0.05)  organic: fosfomycin 22.9% (11.1%) (p<0.05)  MDR no difference | natural selection of resistant isolates, cross-contamination with other animals and humans, other selectors as heavy metals |
| Schwaiger *et al.* (2010) | *Listeria* spp. | Amoxicillin/clavulanic acid, ampicillin, mezlocillin, oxazillin, imipenem, chloramphenicol, florfenicol, ciprofloxacin, enrofloxacin, moxifloxacin, teicoplanin, vancomycin, gentamicin high level, kanamycin, neomycin, streptomycin high level, erythromycin, tylosin, clindamycin, linezolid, quinupristin/dalfopristin, doxycycline, fosfomycin, nitrofurantoin, rifampicin | Germany | farm | cloacal swap | 10 (10) farms | no difference | not mentioned |
| Schwaiger *et al.* (2010) | *Enterococcus* spp. | Amoxicillin/clavulanic acid, ampicillin, mezlocillin, oxazillin, imipenem, chloramphenicol, florfenicol, ciprofloxacin, enrofloxacin, moxifloxacin, teicoplanin, vancomycin, gentamicin high level, kanamycin, neomycin, streptomycin high level, erythromycin, tylosin, clindamycin, linezolid, quinupristin/dalfopristin, doxycycline, fosfomycin, nitrofurantoin, rifampicin | Germany | farm | cloacal swap | 10 (10) farms, 99 (118) isolates | conventional: resistance rates higher (p<0.05) | use of antimicrobials, coexistence of resistance to antimicrobials and heavy metals on same plasmid, resistance transfer within or between species |

**References**

Alali WQ, Thakur S, Berghaus RD, Martin MP and Gebreyes WA 2010. Prevalence and distribution of Salmonella in organic and conventional broiler poultry farms. Foodborne Pathogens & Disease 7, 1363-1371.

Álvarez-Fernández E, Domínguez-Rodríguez J, Capita R and Alonso-Calleja C 2012. Influence of housing systems on microbial load and antimicrobial resistance patterns of *Escherichia coli* isolates from eggs produced for human consumption. Journal of Food Protection 75, 847-853.

Álvarez-Fernández E, Cancelo A, Diaz-Vega C, Capita R and Alonso-Calleja C 2013. Antimicrobial resistance in *E. coli* isolates from conventionally and organically reared poultry: A comparison of agar disc diffusion and Sensi Test Gram-negative methods. Food Control 30, 227-234.

Bennedsgaard TW, Thamsborg SM, Aarestrup FM, Enevoldsen C, Vaarst M and Christoffersen AB 2006. Resistance to penicillin of Staphylococcus aureus isolates from cows with high somatic cell counts in organic and conventional dairy herds in Denmark. Acta Veterinaria Scandinavica 48.

Berge AC, Hancock DD, Sischo WM and Besser TE 2010. Geographic, farm, and animal factors associated with multiple antimicrobial resistance in fecal *Escherichia coli* isolates from cattle in the western United States. Journal of the American Veterinary Medical Association 236, 1338-1344.

Bombyk RAM, Bykowski AL, Draper CE, Savelkoul EJ, Sullivan LR and Wyckoff TJO 2008. Comparison of types and antimicrobial susceptibility of Staphylococcus from conventional and organic dairies in west-central Minnesota, USA. Journal of Applied Microbiology 104, 1726-1731.

Cho SB, Bender JB, Diez-Gonzalez F, Fossler CP, Hedberg CW, Kaneene JB, Ruegg PL, Warnick LD and Wells SJ 2006. Prevalence and characterization of Escherichia coli O157 isolates from Minnesota dairy farms and county fairs. Journal of Food Protection 69, 252-259.

Cho SB, Fossler CP, Diez-Gonzalez F, Wells SJ, Hedberg CW, Kaneene JB, Ruegg PL, Warnick LD and Bender JB 2007. Antimicrobial susceptibility of Shiga toxin-producing *Escherichia coli* isolated from organic dairy farms, conventional dairy farms, and county fairs in Minnesota. Foodborne Pathogens and Disease 4, 178-186.

Cicconi-Hogan KM, Belomestnykh N, Gamroth M, Ruegg RL, Tikofsky L and Schukken YH 2014. Short communication: Prevalence of methicillin resistance in coagulase-negative staphylococci and *Staphylococcus aureus* isolated from bulk milk on organic and conventional dairy farms in the United States. Journal of Dairy Science 97, 2959-2964.

Cohen Stuart J, Van den Munckhof T, Voets G, Scharringa J, Fluit A and Leverstein-Van Hall M 2012. Comparison of ESBL contamination in organic and conventional retail chicken meat. International Journal of Food Microbiology 154, 212-214.

Cui SH, Ge BL, Zheng J and Meng JH 2005. Prevalence and antimicrobial resistance of Campylobacter spp. and Salmonella serovars in organic chickens from Maryland retail stores. Applied and Environmental Microbiology 71, 4108-4111.

Dolejska M, Jurcickova Z, Literak I, Pokludova L, Bures J, Hera A, Kohoutova L, Smola J and Cizek A 2011. IncN plasmids carrying bla CTX-M-1 in Escherichia coli isolates on a dairy farm. Veterinary Microbiology 149, 513-516.

Garcia-Migura L, Pleydell E, Barnes S, Davies RH and Liebana E 2005. Characterization of vancomycin-resistant Enterococcus faecium isolates from broiler poultry and pig farms in England and Wales. Journal of Clinical Microbiology 43, 3283-3289.

Garmo RT, Waage S, Sviland S, Henriksen BIF, Osteras O and Reksen O 2010. Reproductive Performance, Udder Health, and Antibiotic Resistance in Mastitis Bacteria isolated from Norwegian Red cows in Conventional and Organic Farming. Acta Veterinaria Scandinavica 52.

Guarddon M, Miranda JM, Rodriguez JA, Vazquez BI, Cepeda A and Franco CM 2014. Quantitative detection of tetracycline-resistant microorganisms in conventional and organic beef, pork and chicken meat. CyTA Journal of Food 12, 383-388.

Halbert LW, Kaneene JB, Ruegg PL, Warnick LD, Wells SJ, Mansfield LS, Fossler CP, Campbell AM and Geiger-Zwald AM 2006a. Evaluation of antimicrobial susceptibility patterns in Campylobacter spp isolated from dairy cattle and farms managed organically and conventionally in the midwestern and northeastern United States. Journal of the American Veterinary Medical Association 228, 1074-1081.

Halbert LW, Kaneene JB, Linz J, Mansfield LS, Wilson D, Ruegg PL, Warnick LD, Wells SJ, Fossler CP, Campbell AM and Geiger-Zwald AM 2006b. Genetic mechanisms contributing to reduced tetracycline susceptibility of Campylobacter isolated from organic and conventional dairy farms in the midwestern and northeastern United States. Journal of Food Protection 69, 482-488.

Han FF, Lestari SI, Pu SH and Ge BL 2009. Prevalence and antimicrobial resistance among Campylobacter spp. in Louisiana retail chickens after the enrofloxacin ban. Foodborne Pathogens and Disease 6, 163-171.

Heuer OE, Pedersen K, Andersen JS and Madsen M 2001. Prevalence and antimicrobial susceptibility of thermophilic Campylobacter in organic and conventional broiler flocks. Letters in Applied Microbiology 33, 269-274.

Heuer OE, Pedersen K, Andersen JS and Madsen M 2002. Vancomycin-resistant enterococci (VRE) in broiler flocks 5 years after the avoparcin ban. Microbial Drug Resistance-Mechanisms Epidemiology and Disease 8, 133-138.

Hoogenboom LAP, Bokhorst JG, Northolt MD, Van de Vijver LPL, Broex NJG, Mevius DJ, Meijs JAC and Van der Roest J 2008. Contaminants and microorganisms in Dutch organic food products: a comparison with conventional products. Food Additives and Contaminants Part a-Chemistry Analysis Control Exposure & Risk Assessment 25, 1195-1207.

Johnston JR 2002. A comparison of antibiotic resistance in bacteria isolated from conventionally versus organically raised livestock. BIOS (Ocean Grove) 73, 47-51.

Kola A, Kohler C, Pfeifer Y, Schwab F, Kuehn K, Schulz K, Balau V, Breitbach K, Bast A, Witte W, Gastmeier P and Steinmetz I 2012. High prevalence of extended-spectrum-beta-lactamase-producing Enterobacteriaceae in organic and conventional retail chicken meat, Germany. Journal of Antimicrobial Chemotherapy 67, 2631-2634.

Lestari SI, Han FF, Wang F and Ge BL 2009. Prevalence and antimicrobial resistance of Salmonella serovars in conventional and organic chickens from Louisiana retail stores. Journal of Food Protection 72, 1165-1172.

Luangtongkum T, Morishita TY, Ison AJ, Huang SX, McDermott PF and Zhang QJ 2006. Effect of conventional and organic production practices on the prevalence and antimicrobial resistance of Campylobacter spp. in poultry. Applied and Environmental Microbiology 72, 3600-3607.

Mazengia E, Samadpour M, Hill HW, Greeson K, Tenney K, Liao G, Huang X and Meschke JS 2014. Prevalence, concentrations, and antibiotic sensitivities of Salmonella serovars in poultry from retail establishments in Seattle, Washington. Journal of Food Protection 77, 885-893.

McKinney CW, Loftin KA, Meyer MT, Davis JG and Pruden A 2010. tet and sul Antibiotic Resistance Genes in Livestock Lagoons of Various Operation Type, Configuration, and Antibiotic Occurrence. Environmental Science & Technology 44, 6102-6109.

Miranda JM, Vázquez BI, Fente CA, Barros-Velázquez J, Cepeda A and Abuin CMF 2008a. Antimicrobial resistance in Escherichia coli strains isolated from organic and conventional pork meat: a comparative survey. European Food Research and Technology 226, 371-375.

Miranda JM, Vázquez BI, Fente CA, Calo-Mata P, Cepeda A and Franco CM 2008b. Comparison of Antimicrobial Resistance in *Escherichia coli*, *Staphylococcus aureus*, and *Listeria monocytogenes* Strains Isolated from Organic and Conventional Poultry Meat. Journal of Food Protection 71, 2537-2542.

Miranda JM, Mondragon A, Vázquez BI, Fente CA, Cepeda A and Franco CM 2009a. Microbiological quality and antimicrobial resistance of Escherichia coli and Staphylococcus aureus isolated from conventional and organic "Arzua-Ulloa'' cheese. Cyta-Journal of Food 7, 103-110.

Miranda JM, Mondragon A, Vázquez BI, Fente CA, Cepeda A and Franco CM 2009b. Influence of farming methods on microbiological contamination and prevalence of resistance to antimicrobial drugs in isolates from beef. Meat Science 82, 284-288.

Miranda JM, Guarddon M, Mondragon A, Vázquez BI, Fente CA, Cepeda A and Franco CM 2007. Antimicrobial resistance in Enterococcus spp. strains isolated from organic chicken, conventional chicken, and turkey meat: A comparative survey. Journal of Food Protection 70, 1021-1024.

Miranda JM, Guarddon M, Vázquez BI, Fente CA, Barros-Velázquez J, Cepeda A and Franco CM 2008c. Antimicrobial resistance in Enterobacteriaceae strains isolated from organic chicken, conventional chicken and conventional turkey meat: A comparative survey. Food Control 19, 412-416.

Mollenkopf DF, Cenera JK, Bryant EM, King CA, Kashoma I, Kumar A, Funk JA, Rajashekara G and Wittum TE 2014. Organic or antibiotic-free labeling does not impact the recovery of enteric pathogens and antimicrobial-resistant Escherichia coli from fresh retail chicken. Foodborne Pathogens and Disease 11, 920-929.

Nulsen MF, Mor MB and Lawton DEB 2008. Antibiotic resistance among indicator bacteria isolated from healthy pigs in New Zealand. New Zealand Veterinary Journal 56, 29-35.

Ray KA, Warnick LD, Mitchell RM, Kaneene JB, Ruegg PL, Wells SJ, Fossler CP, Halbert LW and May K 2006. Antimicrobial susceptibility of Salmonella from organic and conventional dairy farms. Journal of Dairy Science 89, 2038-2050.

Roesch M, Doherr MG and Blum JW 2006. Management, feeding, production, reproduction and udder health on organic and conventional Swiss dairy farms. Schweizer Archiv Fur Tierheilkunde 148, 387-395.

Sapkota AR, Hulet RM, Zhang GY, McDermott P, Kinney EL, Schwab KJ and Joseph SW 2011. Lower Prevalence of Antibiotic-Resistant Enterococci on US Conventional Poultry Farms that Transitioned to Organic Practices. Environmental Health Perspectives 119, 1622-1628.

Sapkota AR, Kinney EL, George A, Hulet RM, Cruz-Cano R, Schwab KJ, Zhang GY and Joseph SW 2014. Lower prevalence of antibiotic-resistant Salmonella on large-scale US conventional poultry farms that transitioned to organic practices. Science of the Total Environment 476, 387-392.

Sato K, Bartlett PC and Saeed MA 2005. Antimicrobial susceptibility of *Escherichia coli* isolates from dairy farms using organic versus conventional production methods. Javma-Journal of the American Veterinary Medical Association 226, 589-594.

Sato K, Bartlett PC, Kaneene JB and Downes FP 2004a. Comparison of prevalence and antimicrobial susceptibilities of *Campylobacter* spp. isolates from organic and conventional dairy herds in Wisconsin. Applied and Environmental Microbiology 70, 1442-1447.

Sato K, Bennedsgaard TW, Bartlett PC, Erskine RJ and Kaneene JB 2004b. Comparison of antimicrobial susceptibility of *Staphylococcus aureus* isolated from bulk tank milk in organic and conventional dairy herds in the midwestern United States and Denmark. Journal of Food Protection 67, 1104-1110.

Schwaiger K, Schmied EMV and Bauer J 2008. Comparative analysis of antibiotic resistance characteristics of Gram-negative bacteria isolated from laying hens and eggs in conventional and organic keeping systems in Bavaria, Germany. Zoonoses and Public Health 55, 331-341.

Schwaiger K, Schmied EMV and Bauer J 2010. Comparative analysis on antibiotic resistance characteristics of Listeria spp. and Enterococcus spp. isolated from laying hens and eggs in conventional and organic keeping systems in Bavaria, Germany. Zoonoses and Public Health 57, 171-180.

Tikofsky LL, Barlow JW, Santisteban C and Schukken YH 2003. A comparison of antimicrobial susceptibility patterns for Staphylococcus aureus in organic and conventional dairy herds. Microbial Drug Resistance-Mechanisms Epidemiology and Disease 9, S39-S45.

Walk ST, Mladonicky JM, Middleton JA, Heidt AJ, Cunningham JR, Bartlett P, Sato K and Whittam TS 2007. Influence of antibiotic selection on genetic composition of Escherichia coli populations from conventional and organic dairy farms. Applied and Environmental Microbiology 73, 5982-5989.
