# Supplementary material for: Animal Board Invited Review: Comparing conventional and organic livestock production systems on different aspects of sustainability
Source: Animal. 2017 May 31;11(10):1839–51. doi: 10.1017/S175173111700115X (PMC5607874; doi:10.1017/S175173111700115X)
Supplement: Supplementary file 1 [file S175173111700115Xsup.zip › S175173111700115Xsup001/S175173111700115Xsup007.docx]

**Animal Board Invited review: Comparing conventional and organic livestock production systems on different aspects of sustainability**

C.P.A. van Wagenberg, Y. de Haas, H. Hogeveen, M.M. van Krimpen, M.P.M. Meuwissen, C.E. van Middelaar, T.B. Rodenburg

**Supplementary Table S7:** Reviewed studies comparing chemical hazards (residues, toxins, heavy metals) in organic and conventional livestock production

| Reference | Hazard investigated | Study country | Sample point | Sample type | # units/samples: conventional (organic) | | System with lower value (other system value) | | Explanation observed differences | |
| --- | --- | --- | --- | --- | --- | --- | --- | --- | --- | --- |
| *Dairy cattle* |  |  |  |  |  | |  | |  | |
| Almeida-González *et al.* (2012) | organochlorine pesticides (OC) | Spain (Canary Islands) | retail | cheese | 54 (7) brands | | organic: lower levels | | conventional: previous use of pesticides (lindane, cyclodienes) | |
| Gabryszuk *et al.* (2008) | aluminium, lead, arsenic, mercury, cadmium | Poland | farm | milk, hair | 2 (2) farms, 30 (20) cows | | no consistent difference | | amount of grazing (contaminated soil intake, concentration in plants) | |
| Luzardo *et al.* (2012) | organochlorine pesticides (OC) | Spain (Canary Islands) | retail | milk | 16 (10) brands, 96 (60) samples | | organic: total OC 14.49 ng/g fat (27.43) (p=0.003) | | conventional: previous use of pesticides (lindane, endosulfane) | |
| Olsson *et al.* (2001) | cadmium | Sweden | slaughter plant | liver, kidney, muscle, mammary tissue | 1 research station farm, 38 (29) cows | | organic: kidney 330 μg/kg (410) (p<0.05), liver 33 (44) (p<0.05), mammary tissue 0.38 (0.59) (p<0.05)  no differences in muscle | | organic: no phosphate fertilizer on field resulted in decreased levels of Cd in roughage; age of cow (Cd accumulates in time); month in milk production (higher metabolic activity during milking results in faster Cd uptake) | |
| Pattono *et al.* (2011) | Ochratoxin A | ? Italy | retail | milk | 20 (63, of which 15 goat and 9 sheep) samples | | conventional: 0.0% positive samples (4.8%) (p value not provided) | | organic: higher risk of toxin-producing fungi in feed; lower total energy level in feed results in lower protozoa density, which impairs the degradation of ochratoxin A to less toxic ochratoxin α | |
| Rey-Crespo *et al.* (2013) | arsenic, mercury, cadmium, lead, | Spain | farm | milk tank | 10 (22) farms | | no difference | | degree of soil ingestion during grazing | |
| Skaug (1999) | Ochratoxin A | Norway | retail | milk | 40 (47) samples | | no difference | | organic: restricted use fungicides increases risk of fungal infection and ochratoxin A in feed stuffs, longer outdoor period decrease risk of inhaling dust and fungal spores; general: direct contamination of milk under poor hygiene conditions | |
| Tomza-Marciniak *et al.* (2011) | cadmium, lead, arsenic | Poland | farm | blood | 21 (20) farms, 21 (20) samples | | organic: lead 0.007 μg/ml (0.017) (p=0.0017), arsenic 0.002 μg/ml (0.005) (p=0.0026);  no difference in cadmium (p=0.0580) | | organic: less exposed | |
| Tsakiris *et al.* (2015) | DDT | Greece | retail | milk | 154 (42) samples | | No differences in detected or summed residue (p>0.05) concentrations, with few exceptions | | persistence in environment, packaging in plastic showed higher amounts than Tetrapack® bottles, milk processing procedures | |
|  |  |  |  |  |  | |  | |  | |
| *Beef cattle* |  |  |  |  |  |  | |  | |  |
| Blanco-Penedo *et al.* (2009) | arsenic, cadmium, mercury, lead | Spain | slaughter plant | liver, kidney | total 9 farms, 120 calves | | no consistent difference in concentrations | | risk higher at low proportion of concentrate in ration and high levels of grazing (soil ingestion) | |
|  |  |  |  |  |  | |  | |  | |
| *Pigs* |  |  |  |  |  | |  | |  | |
| Lindén *et al.* (2001) | cadmium | Sweden | farm, slaughter plant | liver, kidney, manure | 1 experimental farm, conventional 40 pigs, organic 37/38 (kidney/liver), manure 10 both, soil 10 both | | conventional: kidney 84.0 μg/kg wet weight (96.1) (p<0.005), manure 223 μg/kg dry weight (266) (p<0.02)  no difference in liver | | contaminated soil ingestion, contaminated feed intake (Cadmium concentration in organic feed was lower than in conventional feed), availability in food stuffs | |
| Pozzo *et al.* (2010) | Ochratoxin A | Italy | farm | feed, blood | 11 (4) farms, 22 (8) feed samples, 205 (80) blood samples | | conventional: in blood 0.16 ng/ml (1.32) (p<0.001); in feed 0.61 μg/kg (2.68) (p<0.05) | | organic: only higher because higher concentration in feed due to higher concentration in feed stuffs | |
|  |  |  |  |  |  | |  | |  | |
| *Broilers* |  |  |  |  |  | |  | |  | |
| Nachman *et al.* (2013) | arsenic | USA | retail | chicken breast | 69 (37) breasts | | organic: inorganic arsenic concentrations 0.6 μg/kg (1.8) (p<0.05), roxarsone detected in 0% of samples (50%) (p<0.05);  no difference in total arsenic | | use of drug roxarsone, drinking water | |
| Schiavone *et al.* (2008) | Ochratoxin A | Italy | farm | blood, feed | broiler: 3 (2) farms, 6 (2) feed samples, 30 (13) blood samples | | no difference between systems and animal type | | organic: higher risk of toxin-producing fungi in feed | |
|  |  |  |  |  |  | |  | |  | |
| *Laying hens* |  |  |  |  |  | |  | |  | |
| Luzardo *et al.* (2013) | organochlorine pesticides (OC) | Spain (Canary Islands) | retail | eggs | 12 (12) packages with 6 eggs | | no difference | | organic: outdoor access (eating soil and soil's creatures) | |
| Luzardo *et al.* (2013) | polycyclic aromatic hydrocarbons (PAH) | Spain (Canary Islands) | retail | eggs | 12 (12) packages with 6 eggs | | Organic: 31.29 ng/g (65.95) (p=0.0007) | | Contamination in feed | |
| Schiavone *et al.* (2008) | Ochratoxin A | Italy | farm | blood, feed | laying hen: 3 (2) farms, 6 (6) feed samples, 25 (26) blood samples | | no difference between systems and animal type | | organic: higher risk of toxin-producing fungi in feed | |

**References**

Almeida-González M, Luzardo OP, Zumbado M, Rodríguez-Hernández Á, Ruiz-Suárez N, Sangil M, Camacho M, Henríquez-Hernández LA and Boada LD 2012. Levels of organochlorine contaminants in organic and conventional cheeses and their impact on the health of consumers: An independent study in the Canary Islands (Spain). Food and Chemical Toxicology 50, 4325-4332.

Blanco-Penedo I, Benedito JL, Shore RF, Miranda M, Vaquero MG and Lopez-Alonso M 2009. Influence of farm type (organic, conventional and intensive) on toxic metal accumulation in calves in NW Spain. Agronomy Research 7, 578-584.

Gabryszuk M, Sloniewski K and Sakowski T 2008. Macro- and microelements in milk and hair of cows from conventional vs. organic farms. Animal Science Papers and Reports 26, 199-209.

Lindén A, Andersson K and Oskarsson A 2001. Cadmium in organic and conventional pig production. Archives of Environmental Contamination & Toxicology 40, 425-431.

Luzardo OP, Almeida-Gonzalez M, Henriquez-Hernandez LA, Zumbado M, Alvarez-Leon EE and Boada LD 2012. Polychlorobiphenyls and organochlorine pesticides in conventional and organic brands of milk: Occurrence and dietary intake in the population of the Canary Islands (Spain). Chemosphere 88, 307-315.

Luzardo OP, Rodriguez-Hernandez A, Quesada-Tacoronte Y, Ruiz-Suarez N, Almeida-Gonzalez M, Henriquez-Hernandez LA, Zumbado M and Boada LD 2013. Influence of the method of production of eggs on the daily intake of polycyclic aromatic hydrocarbons and organochlorine contaminants: an independent study in the Canary Islands (Spain). Food & Chemical Toxicology 60, 455-462.

Nachman KE, Baron PA, Raber G, Francesconi KA, Navas-Acien A and Love DC 2013. Roxarsone, inorganic arsenic, and other arsenic species in chicken: a U.S.-based market basket sample. Environmental Health Perspectives 121, 818-824.

Olsson IM, Jonsson S and Oskarsson A 2001. Cadmium and zinc in kidney, liver, muscle and mammary tissue from dairy cows in conventional and organic farming. Journal of Environmental Monitoring 3, 531-538.

Pattono D, Gallo PF and Civera T 2011. Detection and quantification of Ochratoxin A in milk produced in organic farms. Food Chemistry 127, 374-377.

Pozzo L, Cavallarin L, Nucera D and Sara Antoniazzi AS 2010. A survey of ochratoxin A contamination in feeds and sera from organic and standard swine farms in northwest Italy. Journal of the Science of Food and Agriculture 90, 1467-1472.

Rey-Crespo F, Miranda M and López-Alonso M 2013. Essential trace and toxic element concentrations in organic and conventional milk in NW Spain. Food and Chemical Toxicology 55, 513-518.

Schiavone A, Cavallero C, Girotto L, Pozzo L, Antoniazzi S and Cavallarin L 2008. A survey on the occurrence of ochratoxin A in feeds and sera collected in conventional and organic poultry farms in Northern Italy. Italian Journal of Animal Science 7, 495-503.

Skaug MA 1999. Analysis of Norwegian milk and infant formulas for ochratoxin A. Food Additives and Contaminants 16, 75-78.

Tomza-Marciniak A, Pilarczyk B, Ba̧kowska M, Pilarczyk R and Wójcik J 2011. Heavy metals and other elements in serum of cattle from organic and conventional farms. Biological Trace Element Research 143, 863-870.

Tsakiris IN, Goumenou M, Tzatzarakis MN, Alegakis AK, Tsitsimpikou C, Ozcagli E, Vynias D and Tsatsakis AM 2015. Risk assessment for children exposed to DDT residues in various milk types from the Greek market. Food and Chemical Toxicology 75, 156-165.
