# Supplementary material for: Animal Board Invited Review: Comparing conventional and organic livestock production systems on different aspects of sustainability
Source: Animal. 2017 May 31;11(10):1839–51. doi: 10.1017/S175173111700115X (PMC5607874; doi:10.1017/S175173111700115X)
Supplement: Supplementary file 1 [file S175173111700115Xsup.zip › S175173111700115Xsup001/S175173111700115Xsup008.docx]

**Animal Board Invited review: Comparing conventional and organic livestock production systems on different aspects of sustainability**

C.P.A. van Wagenberg, Y. de Haas, H. Hogeveen, M.M. van Krimpen, M.P.M. Meuwissen, C.E. van Middelaar, T.B. Rodenburg

**Supplementary Table S8:** Reviewed studies comparing product quality aspects related to public health (essential elements, fatty acids, vitamins and cholesterol) in organic and conventional livestock production

| Reference | Hazard investigated | Study country | Sample point | Sample type | # units/samples: conventional (organic) | Significantly lower | Explanation observed differences |
| --- | --- | --- | --- | --- | --- | --- | --- |
| *Dairy cattle* |  |  |  |  |  |  |  |
| Adler *et al.* (2013) | selenium | Norway | farm | bulk milk tank | 14 (14) paired farms, 84 (84) samples | conventional: Se concentration (p=0.009) | concentration in concentrate feed |
| Adler *et al.* (2013) | fat-soluble vitamins (α-Tocophreol, β-Carotene, Retinol) | Norway | farm | bulk milk tank | 14 (14) paired farms, 84 (84) samples | no difference (p>0.081) | - |
| Adler *et al.* (2013) | fatty acids | Norway | farm | bulk milk tank | 14 (14) paired farms, 84 (84) samples | conventional: proportion health-beneficial n-3 fatty acids (p<0.001), proportion unhealthy total saturated fatty acids (p=0.001) | n-3 FA: higher intake fish meal; saturated fatty acids: lower energy status of cows |
| Bloksma *et al.* (2008) | omega-3 fatty acids | Netherlands | farm | bulk milk tank | 5 (5) neighbouring farms, 10 (10) samples | conventional: 4.9 mg/g fat (10.6) (p<0.001) | organic: more grass and red clover silage, hay and less concentrate and maize silage |
| Bloksma *et al.* (2008) | CLA | Netherlands | farm | bulk milk tank | 5 (5) neighbouring farms, 10 (10) samples | no difference | organic: more grass and red clover silage, hay and less concentrate and maize silage |
| Butler *et al.* (2011) | CLA, alpha linoleic acid, alpha tocopherol, carotenoids | Italy, Sweden, Denmark, UK | farm | milk | conventional ≤3 farms, organic ≤2 farms per country | conventional: up to 2.5 fold lower | amount of fresh forage, breed |
| Butler *et al.* (2009) | CLA | Wales | farm | bulk milk tank | 5 (5) farms, 16 (20) samples | conventional: total CLA 7.46 mg/g fat (13.33) (p<0.001) +seven isomers lower than organic (p<0.01) | fresh forage intake |
| Gabryszuk *et al.* (2008) | Essential elements Ca, K, Mg, Na, P, S, B, Ba, Co, Cr, Cu, Fe, Ge, I, Li, Mn, Mo, Ni, Se, Si, Sn, Sr, V, Zn | Poland | farm | milk, hair | 2 (2) farms, 30 (20) cows | highest concentrations I, Mn, Sr, V, Zn in milk on conventional intensively producing farm, those of Li, Si, Sn, Ba, Ge on both organic farms. Highest concentrations B, Ba, Co, Fe, Ge, Li in cow hair on organic farm, those of Cr, I, Mo, Se, So, Sr, V, Zn on conventional farm with extensive production. | amount of grazing (control of uptake of sufficient mineral elements) |
| O'Donnell *et al.* (2010) | fatty acids | USA (48 states) | retail | milk | 111 (99) samples, rbST-free 82 | differences minor, not of physiological importance  conventional: saturated fatty acids 62.8% (65.9%) (p<0.001), CLA 0.57% (0.70%) (p<0.001);  organic: monounsaturated fatty acids 26.8% (29.7%) (p<0.001), polyunsaturated fatty acids 4.3% (4.8%) (p<0.001), trans 18:1 fatty acids 2.8% (3.1%) (p<0.001) | dietary components and formulations, rather than management practices |
| Olsson *et al.* (2001) | zinc | Sweden | slaughter plant | liver, kidney, muscle, mammary tissue | 1 research station farm, 38 (29) cows | organic: kidney 19 mg/kg (20) (p<0.05)  conventional: muscle 57 (67) (p<0.05)  no difference liver, mammary tissue | production related |
| Popović-Vranješ *et al.* (2011) | vitamins A, C and α-tocopherol | Serbia | farm | milk | 60 (30) samples | no difference | amount of grazing and fresh grass |
| Popović-Vranješ *et al.* (2011) | fatty acids | Serbia | farm | milk | 60 (30) samples | conventional: polyunsaturated fatty acids 3.13% (3.57% (p<0.01), omega-3 fatty acids 0.53% (0.91%) (p<0.01);  organic: monounsaturated fatty acids 29.25% (30.76%) (p<0.05);  saturated fatty acids, omega-6 fatty acids no difference | amount of grazing and fresh grass |
| Rey-Crespo *et al.* (2013) | Essential elements Co, Cr, Cu, Fe, I, Mn, Mo, Ni, Se, Zn | Spain | farm | milk tank | 10 (22) farms | organic: Cu 41.0 μg/l (51.3) (significant), Zn 3326 (3639) (significant), Se 9.4 (15.3) (significant) | conventional: Cu, Zn, Se supplemented in feed; organic: for I depend more on grazing and more nitrogen fixing crops in field, that lower milk-I concentration through inhibition of the sodium-iodine symporter of the mammary gland; organic: Fe with more soil ingestion |
|  |  |  |  |  |  |  |  |
| *Laying hens* |  |  |  |  |  |  |  |
| Matt *et al.* (2009) | vitamins | ? Estonia | farm | egg yolks | 1 (1) farm, 20 (20) eggs | negligible differences.  conventional: vitamin E β 0.25 mg/100 g yolk (0.36) (p<0.002);  organic: vitamin A 0.46 mg/100g yolk (0.57) (p<0.00006), vitamin D3 0.008 (0.014) (p=0.0006), vitamin E α 6.20 (14.90) (p<0.00001), vitamin E γ 0.22 (0.62) (p=0.0002) | genetics, egg production rate, diet composition |
| Matt *et al.* (2009) | fatty acids | ? Estonia | farm | egg yolks | 1 (1) farm, 20 (20) eggs | no differences | diet composition |
| Matt *et al.* (2009) | cholesterol | ? Estonia | farm | egg yolks | 1 (1) farm, 20 (20) eggs | conventional: 341 mg/100 g (489) (p not provided) | breed, age of hen, management, nutrition |

**References**

Adler SA, Jensen SK, Govasmark E and Steinshamn H 2013. Effect of short-term versus long-term grassland management and seasonal variation in organic and conventional dairy farming on the composition of bulk tank milk. Journal of Dairy Science 96, 5793-5810.

Bloksma J, Adriaansen-Tennekes R, Huber M, Van de Vijver LPL, Baars T and De Wit J 2008. Comparison of organic and conventional raw milk quality in the Netherlands. Biological Agriculture & Horticulture 26, 69-83.

Butler G, Collomb M, Rehberger B, Sanderson R, Eyre M and Leifert C 2009. Conjugated linoleic acid isomer concentrations in milk from high- and low-input management dairy systems. Journal of the Science of Food and Agriculture 89, 697-705.

Butler G, Nielsen JH, Larsen MK, Rehberger B, Stergiadis S, Canever A and Leifert C 2011. The effects of dairy management and processing on quality characteristics of milk and dairy products. (Special Issue: Improving production efficiency, quality and safety in organic and 'low-input' food supply chains.). NJAS Wageningen Journal of Life Sciences 58, 97-102.

Gabryszuk M, Sloniewski K and Sakowski T 2008. Macro- and microelements in milk and hair of cows from conventional vs. organic farms. Animal Science Papers and Reports 26, 199-209.

Matt D, Veromann E and Luik A 2009. Effect of housing systems on biochemical composition of chicken eggs. Agronomy Research 7, 662-667.

O'Donnell AM, Spatny KP, Vicini JL and Bauman DE 2010. Survey of the fatty acid composition of retail milk differing in label claims based on production management practices. Journal of Dairy Science 93, 1918-1925.

Olsson IM, Jonsson S and Oskarsson A 2001. Cadmium and zinc in kidney, liver, muscle and mammary tissue from dairy cows in conventional and organic farming. Journal of Environmental Monitoring 3, 531-538.

Popović-Vranješ A, Savić M, Pejanović R, Jovanović S and Krajinović G 2011. The Effect of Organic Milk Production on Certain Milk Quality Parameters. Acta Veterinaria (Belgrade) 61, 415-421.

Rey-Crespo F, Miranda M and López-Alonso M 2013. Essential trace and toxic element concentrations in organic and conventional milk in NW Spain. Food and Chemical Toxicology 55, 513-518.
